# Rational Design of Phe‐BODIPY Amino Acids as Fluorogenic Building Blocks for Peptide‐Based Detection of Urinary Tract *Candida* Infections

**DOI:** 10.1002/anie.202117218

**Published:** 2022-02-26

**Authors:** Lorena Mendive‐Tapia, David Mendive‐Tapia, Can Zhao, Doireann Gordon, Sam Benson, Michael J. Bromley, Wei Wang, Jun Wu, Adelina Kopp, Lutz Ackermann, Marc Vendrell

**Affiliations:** ^1^ Centre for Inflammation Research The University of Edinburgh EH16 4TJ Edinburgh UK; ^2^ Department Theoretische Chemie Physikalisch-Chemisches Institut Universität Heidelberg 69120 Heidelberg Germany; ^3^ Manchester Fungal Infection Group Division of Evolution Infection and Genomics University of Manchester M139NT Manchester UK; ^4^ Institut für Organische und Biomolekulare Chemie Georg-August-Universität 37077 Göttingen Germany

**Keywords:** Amino Acids, BODIPY, Candida, Fluorescence, Probes, TD-DFT, Excited state, Conical intersection

## Abstract

Fungal infections caused by Candida species are among the most prevalent in hospitalized patients. However, current methods for the detection of Candida fungal cells in clinical samples rely on time‐consuming assays that hamper rapid and reliable diagnosis. Herein, we describe the rational development of new Phe‐BODIPY amino acids as small fluorogenic building blocks and their application to generate fluorescent antimicrobial peptides for rapid labelling of Candida cells in urine. We have used computational methods to analyse the fluorogenic behaviour of BODIPY‐substituted aromatic amino acids and performed bioactivity and confocal microscopy experiments in different strains to confirm the utility and versatility of peptides incorporating Phe‐BODIPYs. Finally, we have designed a simple and sensitive fluorescence‐based assay for the detection of Candida albicans in human urine samples.

## Graphical abstract

The rational design and synthesis of the first fluorogenic Phe‐based BODIPY amino acids, and their application to produce peptide‐based agents for rapid and wash‐free labelling of fungal cells is described. This technology has been exploited to develop a sensitive and low‐cost fluorescence‐based assay for the detection of *Candida albicans* in human urine using simple benchtop spectrophotometers.

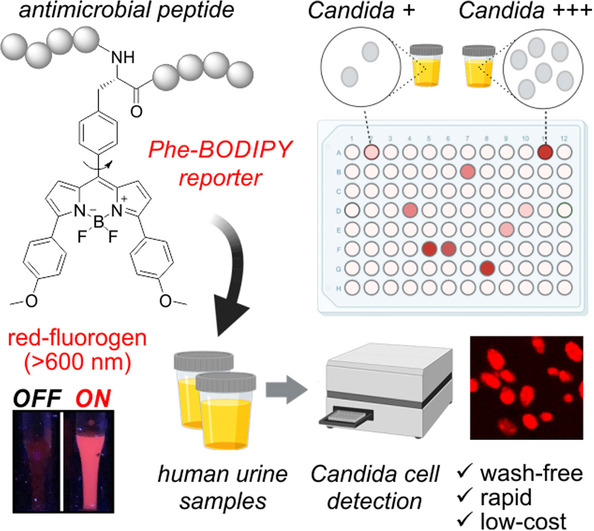

## Introduction

The diagnostic of invasive fungal infections remains a major concern in public health. Over the last decade, the emergence of drug‐resistant fungi have remarkably increased the incidence of hospital‐acquired fungal infections.^[1]^ Among all, infections caused by fungal pathogens of the *Candida* species are among the most frequently diagnosed in hospitalized patients,^[2]^ inducing superficial (e.g., thrush) or disseminated systemic infections (e.g., septicaemia).^[3]^ Conventional assays for the detection of *Candida* cells rely on histopathology, polymerase‐chain reactions and cell cultures, which can take several days because of time‐consuming and multi‐step protocols.^[4]^ As a result, the prescription of adequate treatments is often delayed and can lead to undesired side effects, including multi‐drug resistance due to overuse of empirical antibiotics.

These limitations have prompted the development of simpler and faster approaches for the detection of pathogens in clinical samples.^[5]^ In this context, urine samples are attractive clinical specimens due to their accessibility and direct relevance to urinary tract (UT) infections. The presence of *Candida* species in urine (i.e., Candiduria) is a common nosocomial infection accounting for 5–10 % of all positive urine cultures in hospitals and tertiary care facilities.^[6]^ Despite *Candida* being the most prevalent fungi in patients suffering from UT infections,[^2b^, ^7^] there are no clinically‐approved tests for direct detection of Candiduria.^[8]^

Optical imaging is a powerful tool to investigate essential biological processes in cells.^[9]^ Hence, recent advances in urinalysis have prompted the design of assays using fluorescence readouts for the detection of bacteria and other disease biomarkers.^[10]^ These assays make use of fluorescently‐labelled molecular reporters whose emission is enhanced upon recognition of a specific analyte (e.g., lipids, enzymes).^[11]^ The emergence of fluorescent amino acids (FlAAs) for the construction of peptide‐based imaging reagents has accelerated the chemical design of molecular constructs targeting pathogenic cells.^[12]^ Our group has contributed to the optimisation of metal‐catalysed synthetic methodologies for FlAAs^[13]^ as well as to their application to biological imaging.^[14]^ In particular, the development of FlAAs with fluorogenic character has proven successful to obtain high signal‐to‐noise ratios in bioimaging experiments.^[15]^ In this work, we have rationally designed new fluorogenic amino acids based on the phenylalanine (Phe) core and exploited them to synthesize peptide‐based agents for rapid and direct identification of *Candida* fungal cells in human urine samples using benchtop spectrophotometers.

## Results and Discussion

### Synthesis and Characterisation of BODIPY FlAAs

**Figure 1:**
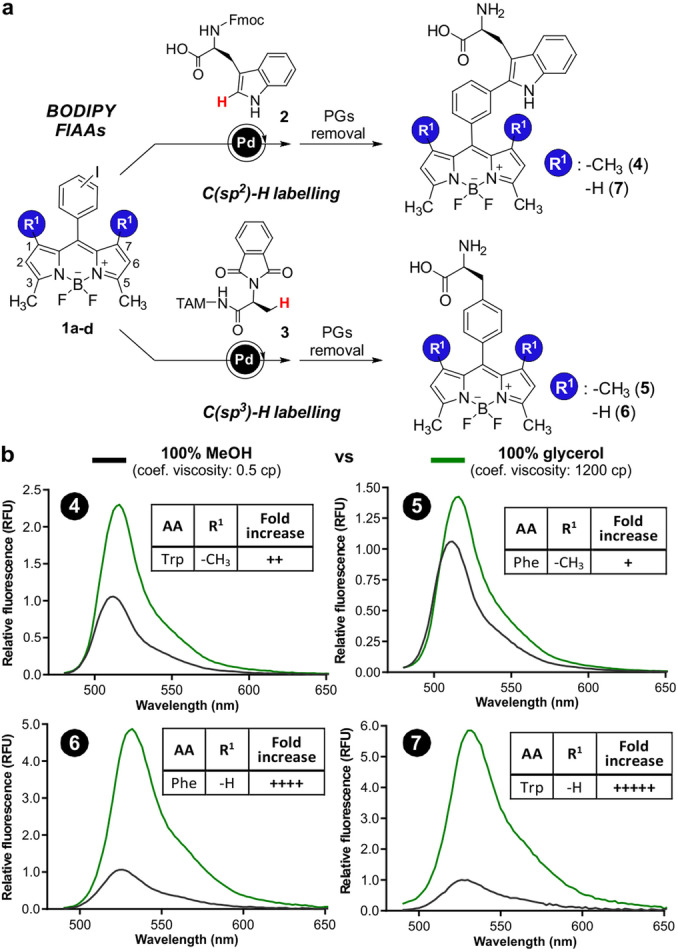
a) Synthetic scheme for the preparation of Trp and Phe‐BODIPY amino acids **4**–**7**. b) Fluorogenic response of amino acids **4**–**7** (20 μM) in MeOH (black) and glycerol (green). *λ*_exc_: 450 nm (for **4** and **5**) and 460 nm (for **6** and **7**) (*n*=3).

The borondipyrromethene (BODIPY) scaffold is an attractive fluorophore for the development of imaging probes due to its excellent photophysical properties.^[16]^ Current approaches for synthesizing BODIPY‐peptide conjugates include classical CuAAC^[17]^ and SPAAC^[18]^ methods using pre‐functionalised intermediates, total solid‐phase approaches for in situ dipyrrin construction on N‐terminal/Lys sites^[19]^ and the use of BODIPY‐bearing FlAAs,^[12a]^ with the latter enabling site‐selective fluorophore incorporation without disrupting the native biomolecular properties of peptides. Among the different BODIPY structures reported for the construction of FlAAs, the fluorogenic Trp‐BODIPY (**4**, Figure 1a) stands out for its utility in wash‐free imaging of biological systems.^[20]^ However, Trp‐BODIPY emits in the green region of the visible spectrum (≈520 nm), which can limit intensity‐based measurements in clinical specimens with some autofluorescence (e.g., urine). Furthermore, the paucity of Trp residues in short bioactive peptides limits its direct replacement in labelling experiments. Alternatively, Phe is a common residue in peptides, including antimicrobial peptides where it plays key roles in cell membrane interaction and subsequent disruption.^[21]^ With this in mind, we decided to explore the design of smaller BODIPY FlAAs based on the more abundant aromatic Phe and with potential to be readily converted into red‐emitting reporters (>600 nm), where the background fluorescence of urine is low.

First, we prepared the Phe‐BODIPY amino acid **5** (Figure 1a) resembling the structure of Trp‐BODIPY (**4**). In this synthesis, we used Pd‐catalysed C(sp^3^)−H arylation^[22]^ to couple a TAM‐protected alanine surrogate and a tetramethyl iodinated BODIPY scaffold. After isolation of the free amino acid **5**, we examined its response in environments with markedly different viscosity (i.e., glycerol vs methanol, with 1200 and 0.5 cp, respectively) and compared it to the Trp‐BODIPY counterpart **4**, obtained using previously reported methods.[^13d^, ^20b^] Interestingly, the simple replacement of Trp by Phe resulted in reduced fluorogenicity, limiting the utility of compound **5** for wash‐free imaging of cells (Figure 1b). In order to improve the environmental sensitivity of Phe‐BODIPY amino acids, we envisaged that the removal of the methyl groups in the positions 1 and 7 of the BODIPY structure could rescue its fluorogenicity without altering the amino acid core or the synthetic approach. As such, we used the same synthetic strategy to obtain the Phe‐BODIPY amino acid **6** (Figure 1a) from the dimethylated BODIPY precursor. In parallel, we also synthesized the dimethylated Trp‐BODIPY (**7**, Figure 1a) to analyse the effect of substitution in Trp‐based FlAAs. Notably, the Phe‐BODIPY **6** showed strong fluorogenic character, with turn‐on fluorescence responses similar to those obtained with Trp‐BODIPY while reducing the overall size of the FlAAs.

### Spectroscopic and Computational Studies

**Figure 2:**
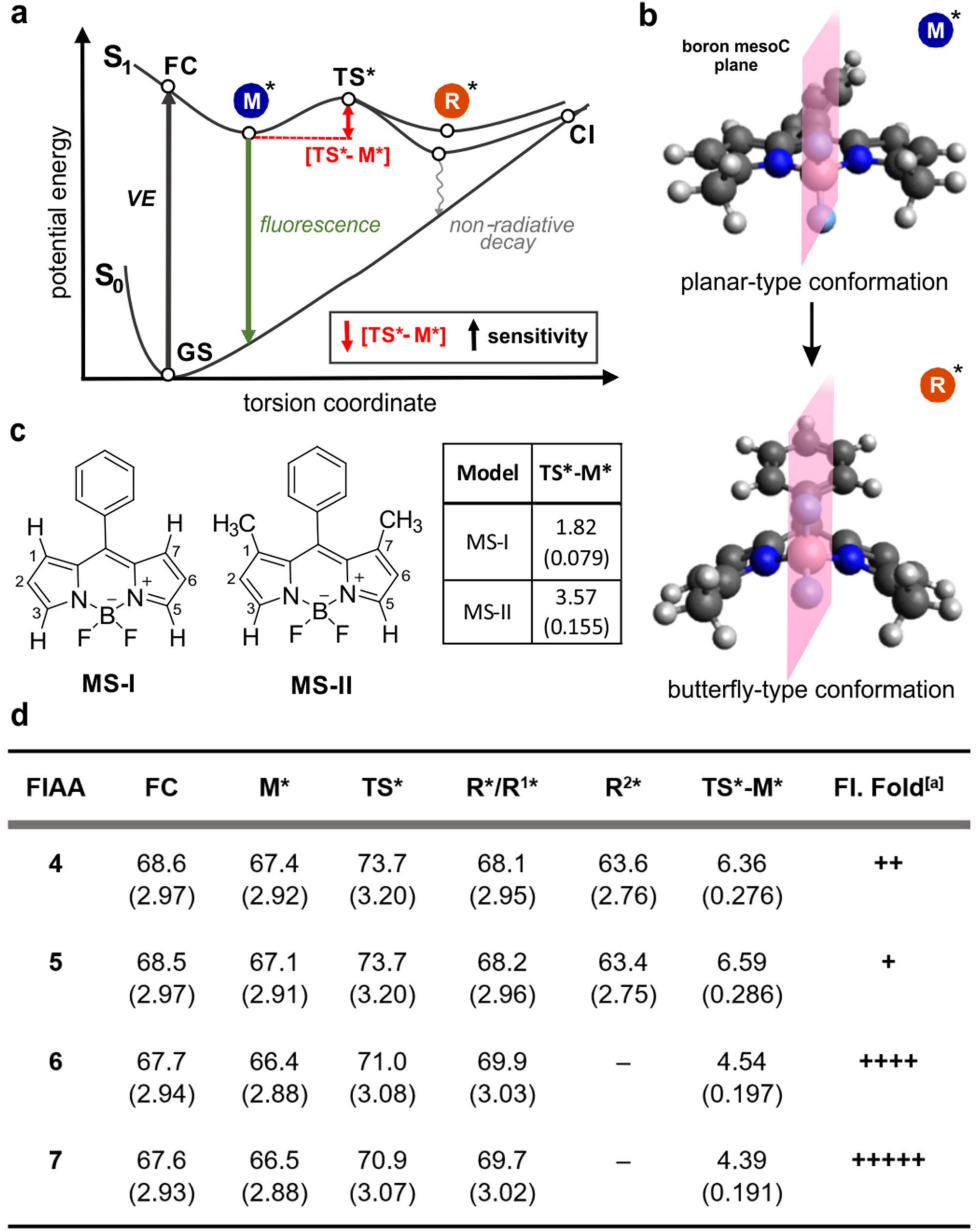
a) Proposed mechanism for the S_1_ excited state reactivity of BODIPY FlAAs. b) Representative example of the optimised excited state geometries for M* and R* minima conformations of BODIPY FlAAs. c) Chemical structure of the BODIPY model systems MS‐I and MS‐II and associated TS*‐M* energy barriers. d) FC, M*, TS* and R* values for amino acids **4**–**7**. For compounds **6** and **7**, the R* conformation is a minimum, while in compounds **4** and **5** this is a transition state (*Cs* symmetry, R^1^*) whose imaginary normal mode of vibration breaks the symmetry into two lower energy conformations (R^2^*).^[a]^ Experimental fold increase observed in Figure 1b. Energy values in kcal mol^−1^ or in eV (brackets).

In order to understand the mechanism behind the behaviour of the different BODIPY‐based amino acids, we investigated the relationship between compounds **4**‐**7** and their fluorogenicity by computational methods. Based on previous studies with other BODIPY dyes,^[23]^ we examined the potential energy surfaces (PESs) of the ground (S_0_) and first excited (S_1_) singlet states alongside the phenyl group torsion coordinates. As shown in Figure 2, upon vertical excitation (VE) from S_0_ to S_1_ at the Frank–Condon (FC) region, the ground state (GS) geometry of the excited state relaxes into the minimum energy conformation (M*). The transition state (TS*) describes the rotation of the phenyl group and controls the accessibility to a second set of conformations (R*/R^1^‐R^2^*) and a near S_1_/S_0_ conical intersection (CI) crossing. As a result, the relaxation of the excited fluorophore can follow two possible pathways: 1) through the CI and with internal energy conversion known as non‐radiative decay,^[24]^ or 2) through radiative decay as fluorescence if the TS energy barrier is large enough to prevent accessing the CI crossing.^[25]^ According to the transition state theory by Grote–Hynes,^[26]^ environmental sensitivity is directly related to the crossing of a low‐frequency barrier. Specifically, PESs with low barriers are associated with environments where solvent frictional forces dominate.^[27]^ In BODIPY fluorophores where accessibility to a CI and consequent non‐radiative decay is controlled by a transition state on the first excited state, the corresponding barrier would define their fluorogenic character.^[28]^ Therefore, we hypothesized that the fluorogenicity of BODIPY FlAAs could be linked to their relative excited energy barriers, that is, the S_1_ energy difference between the TS* and the M* conformations (i.e., TS*‐M*) (Figure 2a). To evaluate this, we optimized the relevant stationary points on the PESs (e.g., FC, M*, TS* and R*) in full dimensionality, rather than simply scanning along the torsion coordinate, using time‐dependent density functional theory (TD‐DFT) with the M06‐2X hybrid exchange‐correlation functional.

First, we analysed the excited state TS* barriers for a series of model systems analogous to the amino acids **4**‐**7** (Figure 2d). We observed a reduction in the energy barrier of the dimethylated Phe‐BODIPY **6** when compared to the tetramethylated Phe‐BODIPY **5** (4.54 kcal mol^−1^ vs. 6.59 kcal mol^−1^, respectively), which accounts for the increased environment sensitivity of the former. A similar trend was observed for the Trp‐BODIPY compounds **4** and **7 (**6.36 kcal mol^−1^ vs. 4.39 kcal mol^−1^), with the dimethylated analogue showing a lower energy barrier and stronger fluorogenicity. In turn, Trp‐BODIPYs **4** and **7** showed lower energy barriers than Phe‐BODIPYs **5** and **6**, potentially due to a positive charge transfer effect from the indole group. In order to gain further insight as to the ordering of the TS barriers and the effects of methyl groups, we also analysed two additional model systems where a phenyl BODIPY structure would bear either hydrogens (MS‐I) or methyl groups (MS‐II) in the positions 1 and 7 (Figure 2c). The non‐substituted MS‐I showed a very small energy barrier value (1.82 kcal mol^−1^) and imaginary frequency at the transition state (64.69 i cm^−1^), which suggest a flat curvature with strong environmental sensitivity. On the other hand, MS‐II displayed an increased energy barrier (3.57 kcal mol^−1^), likely due to steric hindrance of the methyl groups (Figure 2c and Supporting Information Table S1). The methylation in the positions 3 and 5 of the Phe‐BODIPY amino acid **6** also induced a more pronounced increment in the barrier (4.54 kcal mol^−1^). Finally, the tetramethylated Phe‐BODIPY amino acid **5** induced an almost additive effect culminating in the largest barrier (6.59 kcal mol^−1^) observed among all compounds. These barriers were also computed employing the polarizable continuum model (PCM) solvation scheme for solvents with different dielectric constants with similar results (Supporting Information Tables S2, S3). The optimisation of the geometries also confirmed that, upon excitation, Trp‐BODIPY and Phe‐BODIPY amino acids transit from a planar conformation to a butterfly‐type conformation, with substantial phenyl rotation and bending of the BODIPY core on the boron‐mesoC vertical plane (Figure 2b). Overall, these observations corroborate that the lack of methyl groups in the positions 1 and 7 of Phe‐BODIPY FlAAs facilitate the transition to the butterfly minimum R*, which results in increased environmental sensitivity due to the small TS* barrier and flat curvature of the PES.

**Figure 3:**
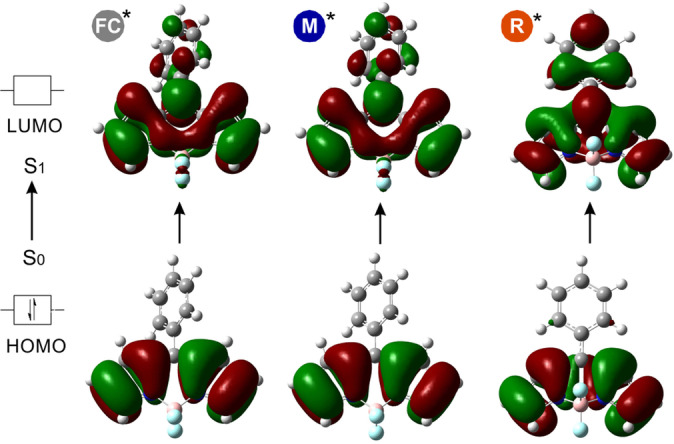
Dominant NTOs upon S_0_→S_1_ transition of MS‐I in the geometries of FC, M* and R*. The corresponding NTO eigenvalues are ≥0.99, signifying that the excitation can be well described in terms of a dominant excitation pair.

Next, we studied whether intramolecular charge transfer (ICT) could be associated with the optical properties of the BODIPY FlAAs. Considering that natural transition orbitals (NTO) offer a good qualitative description of the change in the electronic character upon electronic excitation,^[29]^ we compared these for the FC, M* and R* geometries of MS‐I upon excitation from S_0_ to S_1_ (Figure 3). The respective HOMOs and LUMOs indicated the presence of a π–π* transition with ICT from the BODIPY core to the benzene group. This is more pronounced on the R* geometry due to the higher conjugation between donor and acceptor moieties, which are aligned in the same plane. This analysis indicates that the section of the PES on the M* side of the TS* has little charge transfer character with respect to the ground state, while this is substantial in the section on the R* side. Therefore, electron‐donating or electron‐withdrawing substituents should have a more significant effect on the latter region by respectively stabilizing or destabilizing the PES with respect to the FC region. In agreement with similar analysis performed at the ADC level of theory,^[23]^ these results explain why some substituents stabilize the TS* as the phenyl and BODIPY groups are partially overlapping in this stationary point.

Altogether, our computational studies suggest that the fluorogenic character of Phe‐BODIPY amino acids results from substitution patterns that can be mechanistically rationalized through the analysis of transition state barriers and NTOs at the TD‐DFT level of theory.

### Synthesis and Evaluation of Fluorogenic Peptides Targeting Candida Species

**Figure 4:**
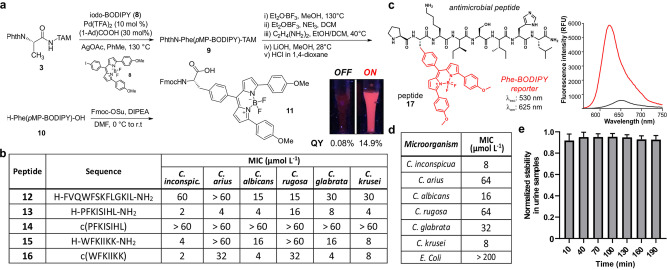
a) Synthetic procedure for the preparation Fmoc‐Phe(*p*MP‐BODIPY)‐OH (**11**). Inset: pictures of solutions of probe **17** (30 μM) under excitation with a 365 nm UV‐lamp in PBS (left) and in liposomes (right). b) Minimum inhibitory concentration (MIC) values of peptides **12**–**16** against different *Candida* strains (5×10^5^ cells mL^−1^) in 20 % Vogel's medium after 48 h incubation at 37 °C. MIC was determined by brightfield microscopy from three independent experiments. c) Chemical structure of peptide **17** and fluorogenic response (25 μM) in PBS (black) and in aqueous suspensions containing liposomes (red). *λ*_exc_: 530 nm (*n*=3). d) MIC values of peptide **17** against different *Candida* strains and bacteria (5×10^5^ cells mL^−1^) in 10 % liquid LB medium after 48 h incubation at 37 °C. MIC was determined by brightfield microscopy from three independent experiments (*n*=3). e) Normalised fluorescence of probe **17** (30 μM) when incubated in diluted urine samples under continuous light irradiation. *λ*_exc_: 530 nm. Data presented as mean±SD (*n*=3).

The activity of antimicrobial peptides as key components of the innate immune system makes them attractive templates for translational applications.^[30]^ Given the compact structure and fluorogenic character of Phe‐BODIPY amino acids, we examined their utility to generate fluorogenic peptides for the detection of *Candida* fungal cells in clinical samples. To overcome any potential interference from the autofluorescence of biological samples (e.g., urine), we fine‐tuned the chemical structure of Phe‐BODIPY **6** by replacing the two methyl substituents with extended *p*‐methoxyphenyl (*p*MP) groups while leaving all the other positions untouched (Figure 4a). Thus, we prepared the Phe‐BODIPY amino acid **10** via the coupling of TAM‐protected alanine surrogate **3** and the diaryl iodinated BODIPY derivative **8** through Pd‐catalysed C(sp^3^)‐H arylation, followed by *C‐* and *N‐*terminal protecting group removal. The resulting Phe‐(*p*MP)BODIPY amino acid **10** retained excellent fluorogenicity with maximum emission wavelengths around 620 nm (Figure S10), which favour good signal‐to‐noise ratios without the need for washing steps. In order to facilitate the integration of Phe‐(*p*MP)BODIPY as a new building block for solid‐phase peptide synthesis (SPPS), we also optimised the synthesis of its Fmoc‐protected amino acid surrogate (compound **11**, Figure 4a). Antimicrobial peptides are known to interact with molecular components of the cell membrane and accumulate in lipophilic intracellular compartments. As expected, compound **11** exhibited remarkable fluorogenic behaviour, remaining silent in aqueous media (QY<0.001) and showing two orders of magnitude higher quantum yields (QY=0.15) in lipophilic microenvironments.

To the best of our knowledge, compound **11** represents the first example of a Phe‐based BODIPY amino acid for SPPS with strong emission in the red region of the visible spectrum. Prior to the incorporation of Phe‐(*p*MP)BODIPY amino acid **11** into peptides that could detect *Candida* fungal cells, we prepared a small collection of Phe‐containing antimicrobial peptides as fungal‐targeting scaffolds that could later accommodate BODIPY‐based aromatic FlAAs (Figure 4b). We selected peptide analogues derived from the natural occurring temporin L (*Rana temporaria*, peptide **12**),^[31]^ jelleine‐I (*Apis mellifera*, peptide **13**)^[32]^ and Aurein1.2 (*Litoria aurea* or *Litoria raniformis*, peptide **15**).^[33]^ This small library was completed with the preparation of the unexplored cyclic versions of peptides **13** and **15**, affording peptides **14** and **16**, respectively. The corresponding unlabelled antifungal peptides **12**‐**16** were synthesized using conventional procedures in SPPS^[34]^ and isolated in excellent purities (>95 %) (full synthetic and characterisation details in the Supporting Information). We examined the activity of peptides **12**‐**16** in 6 different *Candida* strains (*C. krusei, C. albicans, C. glabrata, C. rugosa, C. auris* and *C. inconspicua*) that are representative of fungal infections found in hospitalised patients. In these assays, we observed that the jelleine‐I octapeptide **13** showed the highest biological activity in most *Candida* strains, with working concentrations in the low micromolar range. The mode of action of jelleine‐I is dependent on its tendency to aggregate as of β‐sheet conformations in fungal lipid bilayers.^[35]^ Interestingly, the cyclisation of the linear peptide **13** to afford the peptide **14** led to a complete loss of activity, likely due to a reduced tendency to form β‐strands and accumulate in the lipid bilayers. Thus, we synthesized the corresponding analogue of the linear peptide **13** (Figure 4c, peptide **17**), where we simply replaced Phe from the native sequence with the Phe‐(*p*MP)BODIPY amino acid (Figure S11). The amino acid **11** proved stable to standard reaction conditions for SPPS (e.g., piperidine:DMF for Fmoc removal, DIC/COMU and OxymaPure for amino acid couplings) without observing degradation of the Phe‐(*p*MP)‐BODIPY scaffold, which indicates the compatibility of Phe‐BODIPY amino acids for the synthesis of fluorescent peptides. Upon building of the peptide sequence, we performed the final cleavage from the Sieber amide resin using mild acidic conditions (<5 % TFA in DCM) to preserve the integrity of the BODIPY core and obtained the final peptide **17** after HPLC purification (26 % overall yield, 99 % purity). As anticipated, the peptide **17** showed good fluorogenic behaviour with strong emission around 620 nm (Figure 4c and Figure S12). We next analysed the biological profile of peptide **17** in fungal cells and its selectivity over bacteria. The bioactivity of peptide **17** resembled that of the unlabelled peptide **13** with low micromolar activity in different *Candida* strains (*C. inconspicua, C. albicans, C. krusei*) and minimal bioactivity in bacterial cells (Figure 4d). Furthermore, we evaluated the photo‐ and chemical stability of the peptide **17** in human urine upon continuous light irradiation. In these assays, we ran both fluorescence and HPLC analysis to confirm that the peptide **17** was fully stable for the clinical evaluation of urine samples using fluorescence‐based assays (Figure 4e and Figure S13).

### Fluorescence Imaging of Candida Cells and Detection in Urine Samples

**Figure 5:**
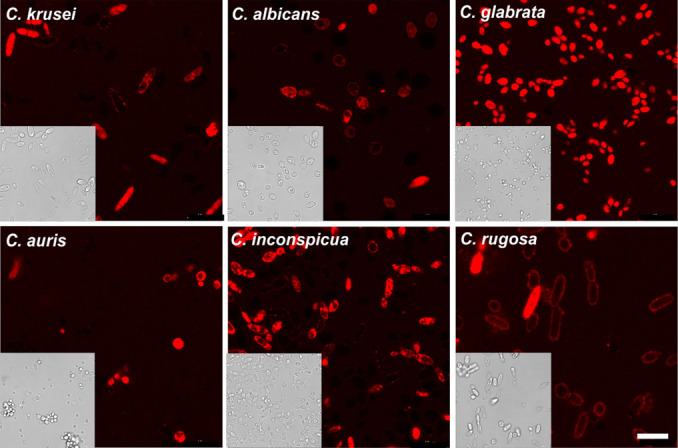
Fluorescence live‐cell confocal microscopy of different *Candida* strains after incubation with peptide **17**. Brightfield (insets) and fluorescence images (5×10^5^ cells mL^−1^) were obtained after 1 h incubation with peptide **17** (10 μM) in PBS at 37 °C without any washings. *λ*_exc_: 575 nm, *λ*_em_: 600–650 nm. Scale bar: 10 μm.

Next, we performed confocal microscopy experiments to evaluate the potential application of the peptide **17** for labelling live cultures of *Candida* fungal cells. We cultured cells of the 6 different *Candida* strains and incubated them with the same concentration of peptide **17** (10 μM) under physiological conditions at 37 °C. Brightfield and fluorescence microscopy images were taken after 1 h incubation without any washing steps (Figure 5). Under these conditions, we observed that all *Candida* strains were brightly labelled and that minimal background signals were detected from peptide **17**. Moreover, we observed that peptide **17** showed brighter readouts in *C. glabrata*, C*. inconspicua* and *C. krusei*, in agreement with the results obtained in bioactivity assays (Figure 5 and Figure S14). We also evaluated peptide **17** selectivity towards *Candida* cells by comparing its labelling with other bacterial and fungal strains commonly found in clinical samples (*E.coli* and *A. fumigatus*, respectively), which showed very weak labelling with peptide **17** (Figure S15).

**Figure 6:**
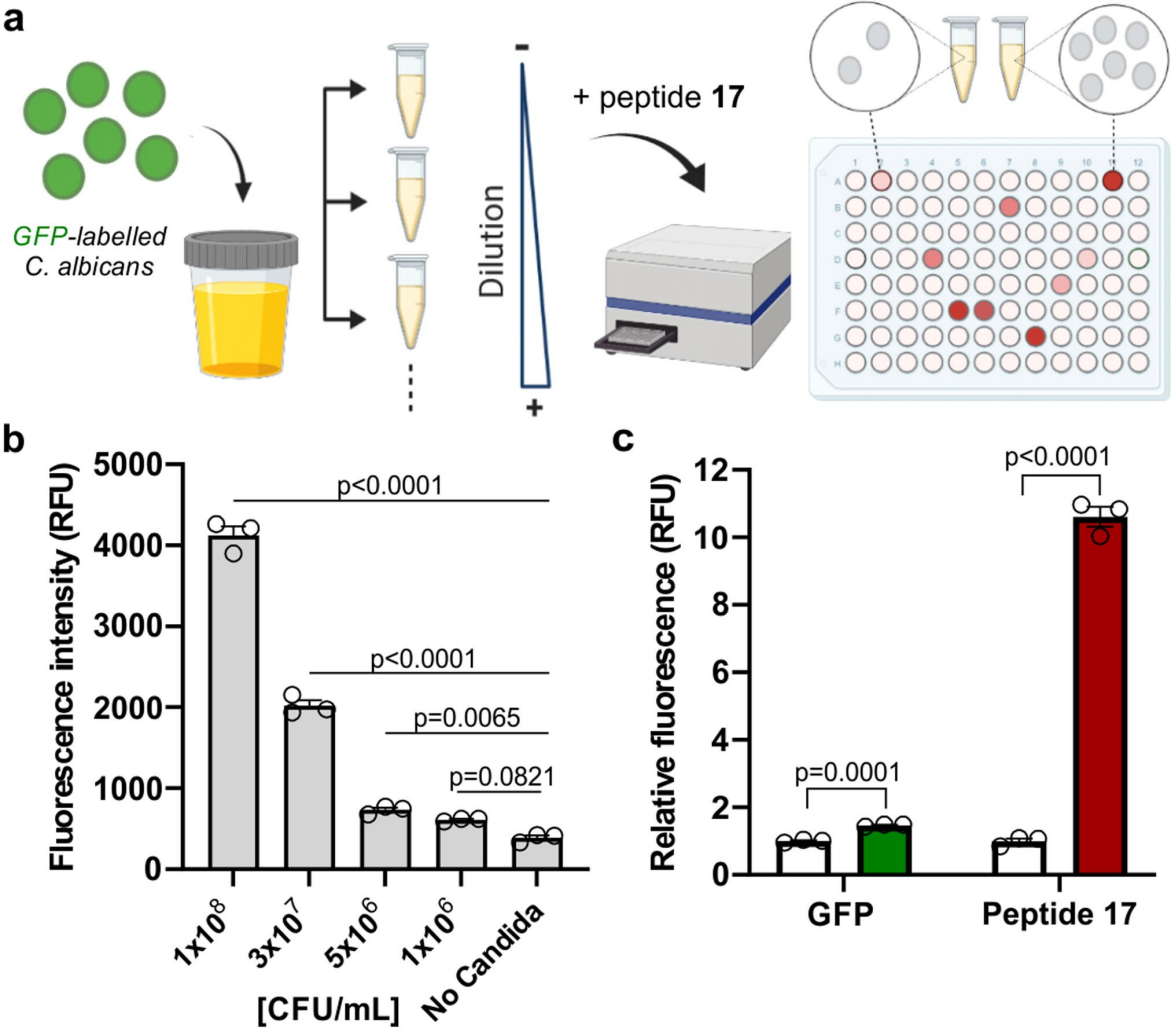
a) Schematic representation of the quantification of *Candida* content in urine samples by fluorescence emission upon incubation with peptide **17** (10 μM) using a benchtop spectrophotometer. b) Fluorescence intensity of peptide **17** (10 μM) upon incubation in urine samples with *C. albicans* ranging from 10^6^ to 10^8^ CFU mL^−1^. Data presented as mean±SEM (*n*=3). P values obtained from ONE‐ANOVA tests with multiple comparisons. c) Relative fluorescence intensity of GFP (green) and peptide **17** (red) upon incubation in urine samples with *C. albicans* (10^8^ CFU mL^−1^) or urine alone (white). *λ*_exc_: 450 nm (GFP) and 530 nm (peptide **17**), *λ*_em_: 515 nm (GFP) and 642 nm (peptide **17**). Data presented as mean±SEM (*n*=3). P values were obtained from unpaired two‐tailed t tests. Representative bar plots from 2 independent experiments with 3 technical replicates.

Finally, we assessed whether the red‐emitting fluorogenic peptide **17** could be applied for quantitative measurements of UT fungal infections in urine samples. *C. albicans* is the most prevalent strain in UT fungal infections,^[36]^ hence we decided to measure fluorescence readouts of human urine containing green fluorescent protein (GFP)‐tagged *C. albicans* cells and to compare them to those obtained after incubation with the peptide **17**. Briefly, GFP‐expressing fungal cells were diluted in urine at serial concentrations from 10^6^ to 10^8^ colony‐forming units (CFU) per mL, and then plated in 384‐well plates before incubation with probe **17** at 37 °C (Figure 6). After 1 h incubation, we directly recorded the fluorescence emission from all wells at 515 nm (GFP) and 642 nm (peptide **17**) in a benchtop spectrophotometer, without any processing or washing steps (Figure 6b and Figure S17). We plotted standard calibration curves for the two fluorescence readouts and determined the corresponding limits of detection (LoD). Notably, the peptide **17** exhibited a remarkable fluorescence response towards *C. albicans* cells with a LoD of 1.8×10^6^±0.6 CFU mL^−1^, which is an order of magnitude lower than the one obtained with the GFP readout (2.6×10^7^±0.4 CFU mL^−1^). Thus, the fluorogenic response of peptide **17** exhibited over 10‐fold fluorescence increase in contrast with the GFP signal, which only showed around 1.5‐fold (Figure 6c). Finally, we also synthetized the same peptide sequence bearing the green fluorescent Trp‐BODIPY **4** (peptide **18**, full synthetic details in the Supporting Information) as a negative control. As expected, peptide **18** showed a smaller 2‐fold turn‐on effect in *Candida* suspensions (Figures S18 and S19). To further evaluate the compatibility of peptide **17** with urine samples, we also confirmed the lack of potential cross‐reactivity of peptide **17** against abundant biomolecules found in urine (e.g., creatinine, urea, amino acids) (Figure S20). These results assert the utility of fluorogenic antimicrobial peptides for the rapid and affordable detection of fungal pathogens in clinical specimens and corroborates the potential of peptide **17** as a probe for the detection of *Candida* cells in human urine samples.

## Conclusion

We have combined spectroscopic and computational methods to rationally design the first fluorogenic Phe‐BODIPY amino acids, expanding the current toolbox of environmentally‐sensitive fluorescent amino acids. Our study indicates that both the substitution pattern and charge‐transfer characteristics of the BODIPY core are pivotal to fine‐tune the fluorogenic character of Phe‐BODIPY FlAAs. As a result, we prepared the Phe‐(*p*MP)BODIPY **11** as a novel red‐emitting building block to generate fluorescent peptides using conventional SPPS. Upon identification of antifungal peptides with affinity for *Candida* cells, we prepared peptide **17** as a fluorogenic probe for rapid and wash‐free detection of *Candida* fungal cells in human urine samples. Peptide **17** shows good selectivity over bacterial cells and high chemical stability, providing a simple, sensitive and low‐cost methodology for *Candiduria* detection. The versatility, compact size and fluorogenicity of Phe‐BODIPYs will enable the preparation of other fluorescent peptides bearing the commonly found Phe residue. Furthermore, we have identified structure–activity relationships in the Phe‐BODIPY core to modulate the energy barriers leading to non‐radiative decay, which will accelerate the future design of BODIPY building blocks with optimal fluorogenic properties.

## Conflict of interest

The authors declare no conflict of interest.

1

## Supporting information

As a service to our authors and readers, this journal provides supporting information supplied by the authors. Such materials are peer reviewed and may be re‐organized for online delivery, but are not copy‐edited or typeset. Technical support issues arising from supporting information (other than missing files) should be addressed to the authors.

Supporting Information

## Data Availability

The data that support the findings of this study are available from the corresponding author upon reasonable request.
