# Supplementary material for: Rational Design of Phe‐BODIPY Amino Acids as Fluorogenic Building Blocks for Peptide‐Based Detection of Urinary Tract Candida Infections
Source: Angew Chem Int Ed Engl. 2022 Feb 26;61(17):e202117218. doi: 10.1002/anie.202117218 (PMC9305947; doi:10.1002/anie.202117218)
Supplement: Supplementary file 1 — Supporting Information [file ANIE-61-0-s001.pdf]

Angewandte Chemie
Eine Zeitschrift der Gesellschaft Deutscher Chemiker

Supporting Information

Rational Design of Phe-BODIPY Amino Acids as Fluorogenic Building Blocks for Peptide-Based Detection of Urinary Tract *Candida* Infections

L. Mendive-Tapia, D. Mendive-Tapia, C. Zhao, D. Gordon, S. Benson, M. J. Bromley, W. Wang, J. Wu, A. Kopp, L. Ackermann, M. Vendrell**

Supplementary Information

Table of Contents

1. Chemical synthesis..2
2. Computational details..10
3. Experimental protocols for spectroscopical and biological assays..11
4. Supplementary figures and tables..14
5. Supplementary references..40

1. Chemical synthesis

General experimental information.

Compounds **1b-d**, **3**, **8** and **9** were synthesized according to modified literature procedures.[1] Other chemicals were obtained from commercial sources and were used without further purification. Thin-layer chromatography was conducted on Merck silica gel 60 F254 sheets and visualized by UV (254 nm and 365 nm). Reactions were monitored by HPLC-MS analysis using a HPLC Waters Alliance HT comprising a Kinetex C18 column (5 µm, 100 Å, 150 x 4.6 mm), a diode array and a MS detector configured with an electrospray ionization source (micromass ZQ4000). A: H_2O (0.1% HCOOH) and B: CH_3CN (0.1% HCOOH) were used as eluents in a gradient from 0-100% B over 8 minutes. Data acquisition was performed with MassLynx software. NMR spectra were recorded on a 500 MHz spectrometer. Chemical shifts (δ) are reported in ppm. Multiplicities are referred by the following abbreviations: s = singlet, d = doublet, t = triplet, dd = double doublet, dt = double triplet and m = multiplet. HRMS (ESI positive) were obtained with a Bruker ESI Micro-TOF mass spectrometer. MALDI analysis was performed on a Bruker UltrafleXtreme MALDI TOF-TOF mass spectrometer. All microwave reactions were carried out in 10 mL sealed glass tubes in a focused mono-mode microwave reactor (Biotage) featured with a surface sensor for internal temperature determination. Cooling was provided by compressed air ventilating the microwave chamber during the reaction. When stated, the final crude was purified via flash column chromatography CombiFlash ISCO RF provided with dual UV detection.

Experimental procedures and characterisation data.

4,4-Difluoro-8-(3-iodophenyl)-3,5-dimethyl -4-bora-*3a,4a*-diaza-*s*-indacene (1a).

3-iodobenzaldehyde (500 mg, 2.2 mmol) was dissolved in anhydrous DCM (50 mL) under N_2. Then, 2-methyl-1*H*-pyrrole (416 µL, 4.7 mmol) and three drops of TFA were added and the reaction was stirred overnight at r.t in N_2 atmosphere. The complete consumption of the aldehyde was checked by TLC. DDQ (489 mg, 2.2 mmol) dissolved in DCM (20 mL) was added dropwise (10-15 min) to the reaction mixture and the reaction was stirred for 15 min at r.t. Finally, TEA (6.3 mL, 45 mmol) and $BF_3{\cdot}OEt_2$ (3.7 mL, 30 mmol) were added, and the mixture stirred overnight at r.t. Workup was done by diluting with DCM (20 mL) and washing with H_2O (2 x 50 mL). The organic layers were combined, dried over sodium sulphate, filtered, and concentrated under vacuum. The crude was purified *via* flash column chromatography

using a EtOAc/hexane gradient on silica gel. The desired compound **1a** was isolated as a red solid (118 mg, 13%).

Characterisation data: ^{1}H NMR (500 MHz, $CDCl_3$): δ = 7.90 – 7.82 (m, 2H), 7.46 (dt, J = 7.8, 1.3 Hz, 1H), 7.22 (t, J = 7.7 Hz, 1H), 6.68 (d, J = 4.2 Hz, 2H), 6.28 (d, J = 4.1 Hz, 2H), 2.65 (s, 6H) ppm. **^{13}C NMR** (126 MHz, $CDCl_3$): δ =158.4, 140.3, 139.0, 138.8, 136.2, 134.4, 130.3, 129.9, 129.7, 119.8, 93.8, 15.1, 1.2 ppm. **HRMS** (ESI+) m/z calcd. for $C_{17}H_{15}N_2BF_2I$ $[M+H]^+$: 423.0336, found, 423.0330.

H-Trp(tetramethyl-BODIPY)-OH (4).

Fmoc-Trp(tetramethyl-BODIPY)-OH (50 mg, 0.066 mmol) was treated with a 20% piperidine/ACN solution (0.4 mL) for 10 min at r.t. The resulting residue was concentrated *in vacuo* and washed with cold Et_2O (5 x 10 mL) to obtain compound **4** as a red solid (31 mg, 90%).

Characterisation data: ^{1}H NMR (500 MHz, DMSO-d_6): δ = 11.30 (s, 1H), 8.03 (d, J = 7.9 Hz, 1H), 7.66 (m, 3H), 7.38 – 7.29 (m, 2H), 7.11 (t, J = 7.5 Hz, 1H), 7.00 (t, J = 7.5 Hz, 1H), 6.19 (d, J = 3.4 Hz, 2H), 3.53 (m, 2H), 3.11 – 3.03 (m, 1H), 2.46 (s, 6H), 1.46 (s, 6H) ppm (traces of piperidine were detected). **^{13}C NMR** (126 MHz, $CDCl_3$): δ = 154.9, 154.8, 143.1, 143.0, 141.6, 136.1, 134.4, 133.9, 133.8, 130.7, 129.6, 128.8, 128.7, 128.0, 126.6, 126.4, 126.3, 121.8, 121.3, 119.0, 118.8, 111.1, 108.8, 55.3, 27.6, 14.2 ppm. **HRMS** (ESI+) m/z calcd. for $C_{30}H_{28}BF_2N_4O_2$ $[M-H]^-$: 525.2279, found, 525.2277.

H-Phe(tetramethyl-BODIPY)-OH (5).

Prepared according to procedure reported elsewhere.[1a]

Characterisation data: ^{1}H NMR (600 MHz, CD_3OD): δ = 7.48 (d, J = 8.1 Hz, 2H), 7.27 (d, J = 8.1 Hz, 2H), 6.04 (s, 2H), 3.85 (dd, J = 7.6, 5.1 Hz, 1H), 3.33 (dd, J = 14.4, 5.1 Hz, 1H), 3.16 (dd, J = 14.4, 7.6 Hz, 1H), 2.47 (s, 6H), 1.42 (s, 6H). **^{13}C NMR** (126 MHz, CD_3OD): δ = 173.2, 156.5, 144.6, 143.1, 138.6, 135.0, 132.5, 131.3, 129.6, 122.1, 57.1, 38.1, 14.9, 14.6 ppm. **HRMS** (ESI+) m/z calcd. for $C_{20}H_{25}BF_2N_3O_2$ $[M+H]^+$: 412.2006, found, 412.2004.

H-Phe(dimethyl-BODIPY)-OH (6).

Prepared according to procedure reported for H-Phe(tetramethyl-BODIPY)-OH (**5**).[1a]

Characterisation data: ^{1}H NMR (300 MHz, CD_3OD): δ = 7.61 – 7.40 (m, 4H), 6.92 – 6.74 (m, 2H), 6.46 – 6.28 (m, 2H), 3.89 (dd, J = 8.3, 4.7 Hz, 1H), 3.42 (dd, J = 14.4, 4.7 Hz, 1H), 3.18 (dd, J = 14.5, 8.3 Hz, 1H), 2.61 (s, 6H). **^{13}C NMR** (75 MHz, CD_3OD): δ = 173.5, 158.8,

143.9, 139.8, 136.5, 135.7, 134.3, 132.1, 131.6, 131.3, 130.5, 120.6, 120.5, 57.4, 38.1, 14.8 ppm. **HRMS** (ESI+) m/z calcd. for $C_{20}H_{21}BF_2N_3O_2$ [M+H]$^+$: 384.1693; found, 384.1695.

Fmoc-Trp(dimethyl-BODIPY)-OH (Fmoc-7).

Compound **1a** (1.5 eq., 59 mg, 0.141 mmol), Fmoc-Trp-OH (**2**) (40 mg, 0.094 mmol), $AgBF_4$ (18 mg, 0.094 mmol), TFA (7.2 µL, 0.094 mmol) and $Pd(OAc)_2$ (1.1 mg, 0.005 mmol) were placed in a microwave reactor vessel in 720 µL DMF. The mixture was heated under microwave irradiation at 80°C for 20 min. The crude was diluted with EtOAc and the resulting suspension was filtered through Celite and concentrated under vacuum. The resulting crude was purified by flash column chromatography using and EtOAc/hexane gradient on silica gel. The expected adduct was isolated as a dark red solid (44 mg, 65%).

Characterisation data: ^{1}**H NMR** (500 MHz, $CDCl_3$): δ = 8.20 (s, 1H), 7.73 (dd, J = 7.6, 3.1 Hz, 2H), 7.68 (d, J = 8.0 Hz, 1H), 7.63 (t, J = 3.3 Hz, 2H), 7.49 – 7.31 (m, 7H), 7.26 – 7.20 (m, 3H), 7.15 (t, J = 7.5 Hz, 1H), 6.80 – 6.69 (m, 2H), 6.22 (d, J = 4.2 Hz, 2H), 5.17 (d, J = 8.1 Hz, 1H), 4.66 (q, J = 6.7 Hz, 1H), 4.28 – 4.13 (m, 2H), 4.08 (t, J = 7.3 Hz, 1H), 3.54 (t, J = 6.3 Hz, 2H), 2.63 (s, 6H) ppm. ^{13}**C NMR** (126 MHz, $CDCl_3$): δ = 175.3, 158.2, 155.9, 143.9, 143.8, 141.5, 141.4, 136.0, 135.2, 135.1, 134.6, 133.0, 130.5, 130.1, 129.7, 129.2, 129.1, 127.9, 127.2, 125.2, 123.2, 120.7, 120.1, 119.9, 119.1, 111.3, 107.6, 67.3, 54.5, 47.2, 29.9, 15.1 ppm. **HRMS** (ESI+) m/z calcd. for $C_{43}H_{34}BF_2N_4O_4Na$ [M+Na]$^+$: 743.2612; found, 743.2615.

H-Trp(dimethyl-BODIPY)-OH (7).

Fmoc-Trp(dimethyl-BODIPY)-OH (**Fmoc-7**, 35 mg, 0.048 mmol) was treated with a 20% piperidine/ACN solution (0.5 mL) for 10 min at r.t. The resulting residue was concentrated *in vacuo* and washed with cold Et_2O (4 x 4 mL) to obtain compound **7** as a dark red solid (17 mg, 71%).

Characterisation data: ^{1}**H NMR** (500 MHz, DMSO-d_6): δ 11.28 (s, 1H), 7.97 (d, J = 7.8 Hz, 1H), 7.80 (s, 1H), 7.71 – 7.61 (m, 2H), 7.56 (d, J = 7.7 Hz, 1H), 7.36 (d, J = 8.0 Hz, 1H), 7.12 (t, J = 7.5 Hz, 1H), 7.03 (t, J = 7.5 Hz, 1H), 6.97 (d, J = 4.2 Hz, 2H), 6.49 (d, J = 4.2 Hz, 2H), 3.59 – 3.50 (m, 2H), 3.08 (m, 1H), 2.57 (s, 6H) ppm. ^{13}**C NMR** (126 MHz, $CDCl_3$): δ 170.2, 157.2, 142.1, 141.2, 136.2, 134.6, 133.7, 133.5, 133.0, 130.9, 130.4, 129.8, 129.3, 128.7, 128.5, 121.7, 120.0, 118.9, 118.8, 111.2, 108.4, 55.2, 27.3, 14.5 ppm. **HRMS** (ESI+) m/z calcd. for $C_{28}H_{24}BF_2N_4O_2Na$ [M+Na]$^+$: 499.2111, found, 499.2132.

H-Phe(*p*MP-BODIPY)-OH (10).

Prepared according to procedure reported elsewhere.[1a]

Characterisation data: ^{1}H NMR (400 MHz, CD_3OD): δ = 7.88 (d, *J* = 9.0 Hz, 4H), 7.60 (d, *J* = 8.1 Hz, 2H), 7.53 (d, *J* = 8.1 Hz, 2H), 6.98 (d, *J* = 9.0 Hz, 4H), 6.94 (d, *J* = 4.4 Hz, 2H), 6.72 (d, *J* = 4.4 Hz, 2H), 3.89 (dd, *J* = 8.2, 4.8 Hz, 1H), 3.86 (s, 6H), 3.43 (dd, *J* = 14.4, 4.8 Hz, 1H), 3.19 (dd, *J* = 14.4, 8.2 Hz, 1H). **^{13}C NMR** (126 MHz, CD_3OD): δ = 162.4, 162.2, 139.6, 137.3, 134.7, 132.2, 132.1, 131.5, 131.4, 130.4, 126.3, 121.4, 114.6, 106.0, 55.8, 38.1, 30.8 ppm. **HRMS** (ESI+) m/z calcd. for $C_{32}H_{29}BF_2N_3O_4$ $[M+H]^+$: 568.2219, found, 568.2215.

Fmoc-Phe(*p*MP-BODIPY)-OH (11).

In a 25 mL round flask, H-Phe(*p*MP-BODIPY)-OH **10** (1.0 eq.) was dissolved in DMF, DIPEA (3.0 eq.) was added at 0 °C, and then Fmoc-OSu (2.0 eq.) was added in portions. The solution was slowly warmed to r.t. and stirred for 16 h, After the reaction, the solution was diluted with DCM, washed with 1N HCl (2 times), H_2O (3 times) and brine, dried over sodium sulfate. The combined organic phase was removed *in vacuo* and the crude product was purified by column chromatography on silica gel (DCM/MeOH 100:1 to 20:1) to yield **11**.

Characterisation data: ^{1}H NMR (400 MHz, DMSO-*d*$_6$): δ = 7.87 (d, *J* = 7.7 Hz, 2H), 7.81 (d, *J* = 8.4 Hz, 4H), 7.67 (t, *J* = 7.8 Hz, 2H), 7.53 – 7.43 (m, 4H), 7.43 – 7.26 (m, 4H), 7.03 (d, *J* = 8.3 Hz, 4H), 6.82 – 6.63 (m, 4H), 4.49 – 4.07 (m, 4H), 3.83 (s, 6H), 3.34 – 3.16 (m, 1H), 3.05 (t, *J* = 11.6 Hz, 1H). **^{13}C NMR** (101 MHz, DMSO): δ = 160.0, 156.7, 155.2, 143.4, 143.2, 141.9, 141.1, 140.2, 140.2, 134.9, 130.9, 130.5, 130.4, 130.4, 130.0, 129.7, 129.0, 127.1, 126.5, 124.8, 124.7, 123.9, 120.3, 119.6, 113.4, 64.9, 55.7, 54.8, 46.2, 36.8 ppm. **HRMS** (ESI+) m/z calcd. for $C_{47}H_{39}BF_2N_3O_6$ $[M+H]^+$: 790.2902, found, 790.2888.

General procedures for SPPS.[2]

Fmoc-Lys(Z)-OH, Fmoc-Trp-OH, Fmoc-Ser(Trt)-OH and Fmoc-Gln-OH were obtained from Sigma-Aldrich and Fmoc-Lys(Mmt)-OH was obtained from Cambridge Bioscience. The remaining Fmoc-amino acids were obtained from Iris Biotech. Coupling reagents COMU, PyOxim and OxymaPure were kindly provided by Luxembourg S3 Biotechnologies. DIPCDI was obtained from Sigma-Aldrich. Sieber Amide and chlorotrityl polystyrene resins were obtained from Merck Novabiochem. Peptides were manually synthesized in 10-mL polystyrene syringes fitted with porous polyethylene discs using common Fmoc-SPPS protocols. Solvents, excess of reagents and soluble by-products were removed by suction. The

Fmoc group was removed with piperidine/DMF (1:4) (1 × 1 min, 2 × 5 min), followed by DMF (x5), and DCM (x5) washes. All syntheses were carried out at room temperature. Peptides bearing fluorescent moieties were always protected from light. Resin loading for 2-chlorotrityl polystyrene resin: the first amino acid was loaded onto the resin using DIPEA (3.0 eq.) in DCM for 10 min followed by additional DIPEA (7.0 eq.) for extra 40 mins. MeOH (0.8 μL/mg resin) was added to cap remaining trityl groups. The resin was then filtered and washed using DCM (5 × 1 min) and DMF (5 x 1 min). The loading of the resin was determined by measuring the absorbance of piperidine-dibenzofulvene adduct at 290 nm using Nanodrop, as an indirect reading of the extent of amino acid coupling. Peptide elongation: After the Fmoc group was removed, the resin was washed with DMF (4 × 1 min), DCM (3 × 1 min), DMF (4 × 1 min). Coupling was carried out using Fmoc-AA-OH (4 eq.), coupling reagent (4 eq.), OxymaPure (4 eq.) and DIPEA (8 eq.) in DMF for 1 h. The resin was then washed with DMF (5 × 1 min), DCM (5 × 1 min) and filtered. The completion of the coupling step was confirmed using Kaiser Test. Before the next coupling cycle, Fmoc group is removed as described above. Cleavage from resin for compounds **12** and **14-17**: The peptide was cleaved from the resin using 2% TFA, 2.5% TIS in DCM (5 × 1 min) (**12**, **15** and **17**) or 2% TFA/DCM (**14** and **16**) and washed with DCM (2 × 1 min). The combined filtrates were collected into a round bottom flask containing DCM (10 mL) and concentrated under reduced pressure. Cleavage from resin for compound **13**: The peptide was cleaved from the resin using 95% TFA, 2.5% TIS in DCM (1h) and washed with DCM (4 × 1 min). The combined filtrates were collected into a round bottom flask and concentrated under reduced pressure.

Purifications were conducted in a semi-Preparative Agilent HPLC consisting of a 1220 Infinity II autosampler and a 1260 Infinity II detector. Kinetex 150 × 21.2 mm (5 μm) C18 column was used, together with H_2O (0.1% HCOOH) and CH_3CN (0.1% HCOOH) as eluents and a flow rate of 8 mL min^{-1}.

H-Phe-Val-Gln-Trp-Phe-Ser-Lys-Phe-Leu-Gly-Lys-Ile-Leu-NH_2 (12).

The synthesis was performed on 22 mg of Sieber Amide resin (0.65 mmol/g). Fmoc-Leu-OH, Fmoc-Ile-OH, Fmoc-Lys(z)-OH, Fmoc-Gly-OH, Fmoc-Phe-OH, Fmoc-Ser(Trt)-OH, Fmoc-Trp-OH, Fmoc-Gln-OH and Fmoc-Val-OH were used as side-chain protected building blocks. After cleavage as described above, the crude peptide was precipitated by adding cold Et_2O (dropwise) and the resulting precipitate was decanted and dried (x2), obtaining 29 mg of benzyloxycarbonyl (Z) lysine-protected peptide. The crude peptide (24 mg, 0.013 mmol) was

deprotected by means of hydrogenation. Peptide was dissolved in HCOOH/DMF/MeOH (0.05:3.3:1) (1.9 mL), followed by addition of 20% $Pd(OH)_2/C$ (6.1 mg, ca. quarter of peptide mass). Then, the reaction flask was flushed with N_2/vacuum cycles (×3) and filled with H_2. The reaction mixture was stirred under H_2 at r.t. for 1 h (monitored by HPLC-MS). The catalyst was removed by filtration under Celite and washed with MeOH. Filtrate was collected in a round bottom flask and solvent was removed under reduced pressure. The residue was dissolved in CH_3CN:H_2O and lyophilised. Purification was conducted by semi-Preparative HPLC using a 0-50% gradient over 25 min, with detection at 220 and 280 nm. Pure fractions were collected and lyophilised to afford pure peptide **12** as a white solid (2.3 mg, 10% yield). **Characterisation data: HPLC-MS**: t_R: 5.7 min (99% purity). **HRMS** (ESI+) m/z calcd. for $C_{83}H_{121}N_{18}O_{15}$ $[M-H]^-$: 1609.9264, found: 1609.9289. **MALDI** (m/z): $[M+H]^+$: 1611.9401, $[M+Na]^+$: 1633.9233.

H-Pro-Phe-Lys-Ile-Ser-Ile-His-Leu-NH_2 (13).

The synthesis was performed on 52 mg of Sieber Amide resin (0.57 mmol/g). Fmoc-Leu-OH, Fmoc-His(Trt)-OH, Fmoc-Ile-OH, Fmoc-Ser(Trt)-OH, Fmoc-Lys(Mmt)-OH, Fmoc-Phe-OH and Fmoc-Pro-OH were used as building blocks, After cleavage as described above, the fully deprotected peptide was precipitated by adding cold Et_2O (dropwise) and the resulting precipitate was decanted and dried (×2). Purification was conducted by semi-Preparative HPLC using a 0-50% gradient over 25 min, with detection at 220 and 260 nm. Pure fractions were collected and lyophilised to afford pure peptide **13** as a white solid (14 mg, 50% yield). **Characterisation data: HPLC-MS**: t_R: 5.2 min (98% purity). **HRMS** (ESI+) m/z calcd. for $C_{47}H_{77}N_{12}O_9$ $[M+H]^+$: 953.5931, found: 953.5934. **MALDI** (m/z): $[M+H]^+$: 953.5941, $[M+Na]^+$: 975.5724.

Cyclo(-Pro-Phe-Lys-Ile-Ser-Ile-His-Leu-) (14).

The synthesis was performed on 50 mg of 2-chlorotrityl polystyrene resin (0.59 mmol/g). Fmoc-Pro-OH, Fmoc-Leu-OH, Fmoc-His(Trt)-OH, Fmoc-Ile-OH, Fmoc-Ser(Trt)-OH, Fmoc-Lys(Boc)-OH and Fmoc-Phe-OH were used as building blocks. After cleavage as described above, the peptide crude was precipitated by adding cold Et_2O (dropwise) and the resulting precipitate was decanted and dried to afford 36 mg of a white solid corresponding to the protected peptide. Then, 16 mg of cleaved peptide (1.0 eq.), PyOxim (1.5 eq.) and OxymaPure (1.5 eq.) were dissolved in DMF:ACN (1:1, 0.001 M). After setting the cocktail at -10 ˚C using a salted ice bath, DIPEA (3.0 eq.) was added, and the mixture stirred overnight at r.t. After

solvent removal under reduced pressure, the crude peptide was dissolved in TFA:TIS:H_2O (95:2.5:2.5) for 1h to remove the side-chain protecting groups. Then, the crude was concentrated under reduced pressure followed by precipitation in cold Et_2O (dropwise). Purification was conducted by semi-Preparative HPLC using a 0-60% gradient over 25 min, with detection at 220 and 260 nm. Pure fractions were collected and lyophilised to afford pure peptide **14** as a white solid (3.0 mg, 26% yield from cyclization step).

Characterisation data: HPLC-MS: t_R: 5.7 min (>99% purity). **HRMS** (ESI+) m/z calcd. for $C_{47}H_{74}N_{11}O_9$ $[M+H]^+$: 936.5665, found: 936.5652. **MALDI** (m/z): $[M+H]^+$: 936.5665, $[M+Na]^+$: 958.5484.

H-Trp-Phe-Lys-Ile-Ile-Lys-Lys-NH_2 (15).

The synthesis was performed on 52 mg of Sieber Amide resin (0.57 mmol/g). Fmoc-Lys(Mmt)-OH, Fmoc-Ile-OH, Fmoc-Phe-OH and Fmoc-Trp-OH were used as building blocks. After cleavage as described above, the fully deprotected peptide was precipitated by adding cold Et_2O (dropwise) and the resulting precipitate was decanted and dried (×2). Purification was conducted by semi-Preparative HPLC using a 0-50% gradient over 25 min, with detection at 220 and 280 nm. Pure fractions were collected and lyophilised to afford pure peptide **15** as a white solid (2.6 mg, 9% yield).

Characterisation data: HPLC-MS: t_R: 4.7 min (95% purity). **HRMS** (ESI+) m/z calcd. for $C_{50}H_{81}N_{12}O_7$ $[M+H]^+$: 961.6346, found: 961.6366. **MALDI** (m/z): $[M+H]^+$: 961.6344, $[M+Na]^+$: 983.6168.

Cyclo(-Trp-Phe-Lys-Ile-Ile-Lys-Lys-) (16).

The synthesis was performed on 50 mg of 2-chlorotrityl polystyrene resin (0.63 mmol/g). Fmoc-Lys(Boc)-OH, Fmoc-Ile-OH, Fmoc-Phe-OH and Fmoc-Trp-OH were used as building blocks. After cleavage as described above, the peptide crude was precipitated by adding cold Et_2O (dropwise) and the resulting precipitate was decanted and dried to afford 36 mg of a white solid corresponding to the protected peptide. Then, 16 mg of cleaved peptide (1.0 eq.), PyOxim (1.5 eq.) and OxymaPure (1.5 eq.) were dissolved in DMF:ACN (1:1, 0.001 M). After setting the cocktail at -10˚C using a salted ice bath, DIPEA (3.0 eq.) was added and the mixture stirred for 2 days at r.t. After solvent removal under reduced pressure, the crude peptide was dissolved in TFA:H_2O:DCM (30:2.5:67.5) for 40 min to remove the side-chain protecting groups. Then, the crude was concentrated under reduced pressure followed by precipitation in cold Et_2O (dropwise). Purification was conducted by semi-Preparative HPLC using a 0-50% gradient

over 25 min, with detection at 220 and 280 nm. Pure fractions were collected and lyophilised to afford pure peptide **16** as a white solid (1.9 mg, 16% yield from cyclization step).

Characterisation data: HPLC-MS: t_R: 6.4 min (96% purity). **HRMS** (ESI+) m/z calcd. for $C_{50}H_{78}N_{11}O_7$ $[M+H]^+$: 944.6080, found: 944.6060. **MALDI** (m/z): $[M+H]^+$: 944.6082.

H-Pro-Phe(*p*MP-BODIPY)-Lys-Ile-Ser-Ile-His-Leu-NH$_2$ (17).

The synthesis was performed on 8.6 mg of Sieber Amide resin (0.57 mmol/g). Fmoc-Leu-OH, Fmoc-His(Mmt)-OH, Fmoc-Ile-OH, Fmoc-Ser(Trt)-OH, Fmoc-Ile-OH, Fmoc-Lys(Mmt)-OH, Fmoc-Phe(*p*MP-BODIPY)-OH (**11**) and Fmoc-Pro-OH were used as building blocks. After cleavage as described above, the fully deprotected peptide was precipitated by adding cold Et_2O (dropwise) and the resulting precipitate was decanted and dried (x2). Purification was conducted by semi-Preparative HPLC using a 20-60% gradient over 25 min, with detection at 220 and 572 nm. Pure fractions were collected and lyophilised to afford pure peptide **17** as a purple solid (1.7 mg, 26% yield).

Characterisation data: HPLC-MS: t_R: 4.75 min (99% purity). **HRMS** (ESI+) m/z calcd. for $C_{70}H_{93}N_{14}BF_2O_{11}$ [M]: 1354.7204, found: 1354.7236. **MALDI** (m/z): $[M+H]^+$: 1355.7290, $[M+Na]^+$: 1377.7183.

H-Pro-Trp(tetramethyl-BODIPY)-Lys-Ile-Ser-Ile-His-Leu-NH$_2$ (18).

The synthesis was performed on 15 mg of Sieber Amide resin (0.57 mmol/g). Fmoc-Leu-OH, Fmoc-His(Mmt)-OH, Fmoc-Ile-OH, Fmoc-Ser(Trt)-OH, Fmoc-Ile-OH, Fmoc-Lys(Mmt)-OH, Fmoc-Trp-BODIPY-OH and Fmoc-Pro-OH were used as building blocks. After cleavage as described above, the fully deprotected peptide was precipitated by adding cold Et_2O (dropwise) and the resulting precipitate was decanted and dried (x2). Purification was conducted by semi-Preparative HPLC using a 5-100% gradient over 15 min, with detection at 220 and 500 nm. Pure fractions were collected and lyophilised to afford pure peptide **18** as an orange solid (3.0 mg, 27% yield).

Characterisation data: HPLC-MS: t_R: 5.65 min (>99% purity). $[M+H]^+$: 1314.8, $[M+Na]^+$: 1337.8.

2. Computational details

DFT and TD-DFT calculations were performed with the M06-2X hybrid exchange-correlation functional[3] and the 6-311+G(2d,p) Pople basis set as implemented in the Gaussian 09[4] package. This choice is supported by previous benchmarks performed on aza-BODIPY and BODIPY dyes,[5] which demonstrate that this level of theory provides good consistency with experimental trends for optical spectra, yet a systematic overshooting of the transition energies (by c.a. 0.4 eV). However, this systematic error is not a concern for the present study as we are not interested in theoretically reproducing the experimental spectra, rather than comparing the different molecules studied (e.g., transition state barriers) on the same footing. Numerical frequency calculations were used to ascertain the nature of the stationary points and the same increased integration grid (i.e., ultrafine (99,590)) respect to the default setting was used in all computations as this is recommended for describing correctly very low frequency modes.

3. Experimental protocols for spectroscopical and biological assays

Fluorescence spectra and intensity acquisition.

Absorbance and emission spectra were determined in the range of 400–700 nm (every 2 nm) at the indicated concentrations on 96 or 384-well plates using a BioTek Cytation 3 spectrophotometer. Environmental sensitivity was measured by comparing the fluorescence emission in MeOH vs glycerol (compounds **4-7**) and in presence of liposome suspensions in PBS or PBS alone (compound **17**).

Measurement of extinction coefficients.

For extinction coefficient measurements, the absorbance of each sample at the maximum excitation wavelength was recorded and the extinction coefficient was then determined by fitting the data to Beer's law.

Measurement of quantum yields.

Quantum yields were determined by measuring the integrated emission area of the fluorescence spectra in PBS or in the presence of phosphatidylcholine: cholesterol liposome suspensions in PBS and comparing it to the area measured for a reference compound rhodamine 101 in MeOH as the reference compound.[6] Different working solutions of compounds and reference ranging from 5 and 100 µM were prepared and the absorbance and fluorescence spectrum of each sample (560-750 nm) was measured with excitation at 530 nm. The quantum yields (QY) were determined using the equation $QY = QY_{ref} \times (m/m_{ref}) \times (n^2/n_{ref}^2)$, where m is the slope of the line obtained from the plot of the area of fluorescence vs. absorbance, n is the refractive index of the solvent and the subscript *ref* refers to the reference of known quantum yield. Values were obtained as means from three independent experiments with n=3.

Stability tests of peptide 17 in urine samples.

To assess photo- and urine stability, probe **17** was added into diluted urine samples (urine: water, 1:6) and incubated in 384-well plates at 37°C. For photostability determination, the fluorescence emission (644 nm) of probe **17** (30 µM) was monitored using a BioTek Cytation 3 spectrophotometer. Values were obtained as means from three independent experiments with n=3. To determine chemical integrity, the absorbance (570 nm) of probe **17** (100 µM) was monitored by HPLC-MS at different timepoints.

Culture of fungal strains.

All strains used in this experiment were grown on SAB agar at 37 °C for 3 days. Cells were harvest using sterile inoculation loop by taking a single colony and resuspending in PBS supplemented with 0.1% tween-20 (PBST). The concentration of cells was then quantified with a haemocytometer. For the determination of the minimum inhibitory concentration, cell density was adjusted to 10^6 cells mL^{-1} with 20% liquid Vogel's medium.

Culture of *E. coli*.

E. coli was grown on Lysogeny Broth (LB) agar at 37 °C for 1 day and harvested using sterile inoculation loop by taking a single colony and resuspending in PBST. The number of cells was quantified using a haemocytometer. For the determination of the minimum inhibitory concentration, cell density was adjusted to 10^6 cells mL^{-1} with 20% liquid LB medium.

***In vitro* measurements of minimum inhibitory concentrations.**

Minimum inhibition concentration (MIC) measurements were performed as described previously with minor changes.[7] Each compound was dissolved in DMSO at concentration of 100 mM, this was used as the stock solution. For testing the MIC, the stock solution was further diluted in water to reach a concentration of 1 mM and was added in to a 96-well plate cell culture plate. A serial dilution was then performed within the 96-well plate, and the compound solutions at different concentrations were then mixed with conidia suspended in 20% Vogel's medium to reach a final volume of 100 µL per well. The final conidia concentration was 5×10^5 cells mL^{-1} in 20% Vogel's medium, the highest tested concentration of each compound was at 50 mg mL^{-1}. After 48 h incubation at 37°C, MIC was determined by brightfield microscopy from three independent experiments (n = 3). For testing the MIC against *E. coli*, the same protocol was followed apart from using liquid LB as the medium at a final concentration of 10%.

Confocal live-cell microscopy.

Probe **17** was mixed with *Candida spp.* to reach a final concentration of 10 µM and a final cell concentration of 5×10^5 cells/mL in PBS. The cells combined with the peptide were dispensed into the wells of Ibidi µ-slide 8 well (Ibidi GmbH, Germany) and incubated for 10 min at r.t. Live cell imaging of the germinated spores was performed using a Leica TCS SP8 confocal laser scanning microscope equipped with photomultiplier tubes, hybrid GaAsP detectors and a 63× water immersion objective and white light laser (575 nm was used for excitation

wavelength and 600-650 nm was used for emission). Images were taken at 15, 30 and 60 min timepoints. Images were processed using Imaris software 8.0 developed by Bitplane (Zurich, Switzerland).

Measurements of the limit of detection.

The limit of detection (LoD) was determined by fluorescence titration of serial dilutions of *C. albicans* cells spiked into human urine samples from healthy donors. For probe **17**, samples were previously incubated with probe **17** for 1 h at 37 °C. The colony forming units (CFU) were determined from the interpolation of a standard curve from the plotting of the CFU counting of a series of *C. albicans* cultures vs. their associated OD_{600} measurements. The LOD was calculated using the equation LoD = (3 x σ)/*k*, were σ is the standard deviation of blank solutions and *k* is the slope of the linear regression fit from the fluorescence emission vs. *Candida* concentration [CFU mL^{-1}] curve. The LoD values were determined from the average of two independent experiments.

4. Supplementary figures and tables

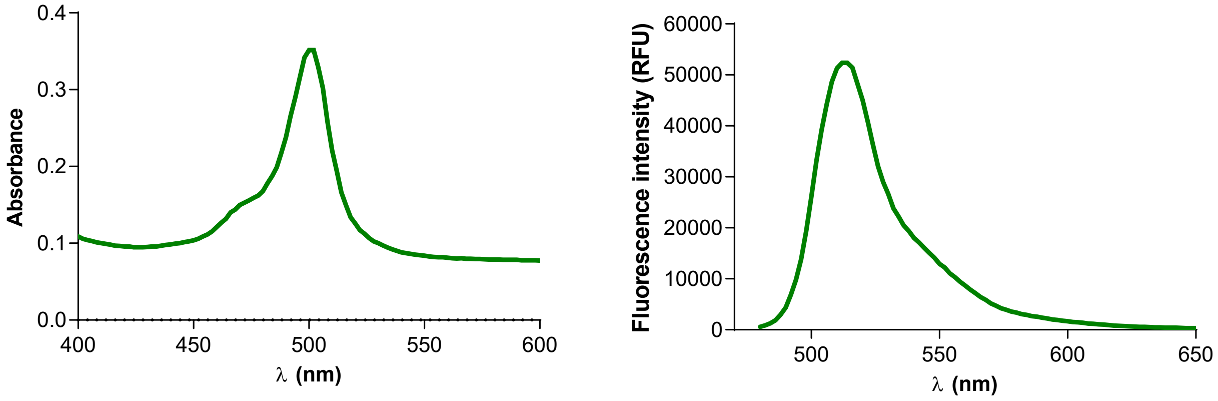

Supplementary Figure S1. Absorbance (left) and emission (right) spectra of amino acid **4** (20 μM) in EtOH. λ_{exc}: 450 nm (n=3).

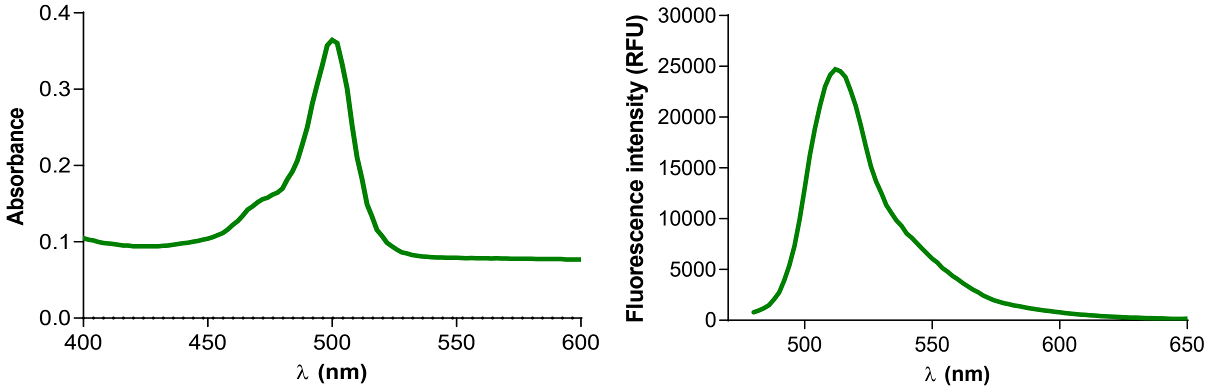

Supplementary Figure S2. Absorbance (left) and emission (right) spectra of amino acid **5** (20 μM) in EtOH. λ_{exc}: 450 nm (n=3).

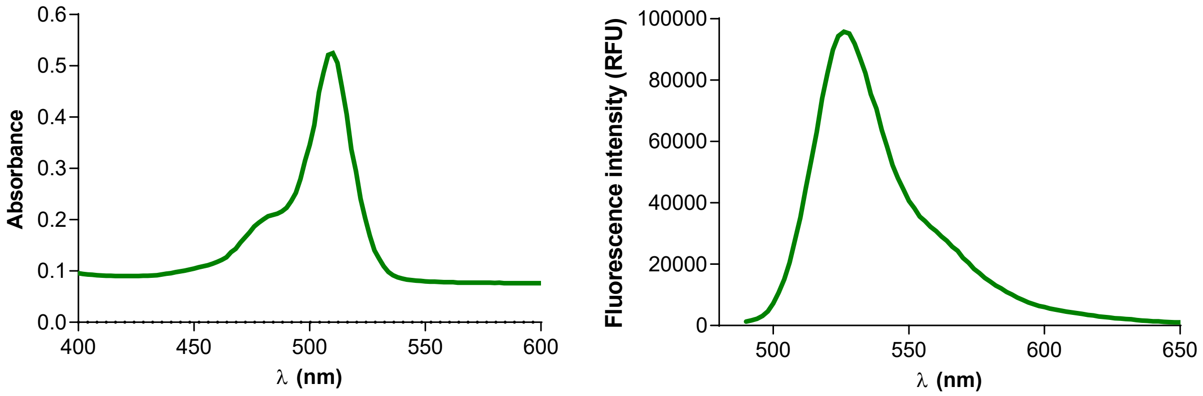

Supplementary Figure S3. Absorbance (left) and emission (right) spectra of amino acid **6** (20 μM) in EtOH. λ_{exc}: 460 nm (n=3).

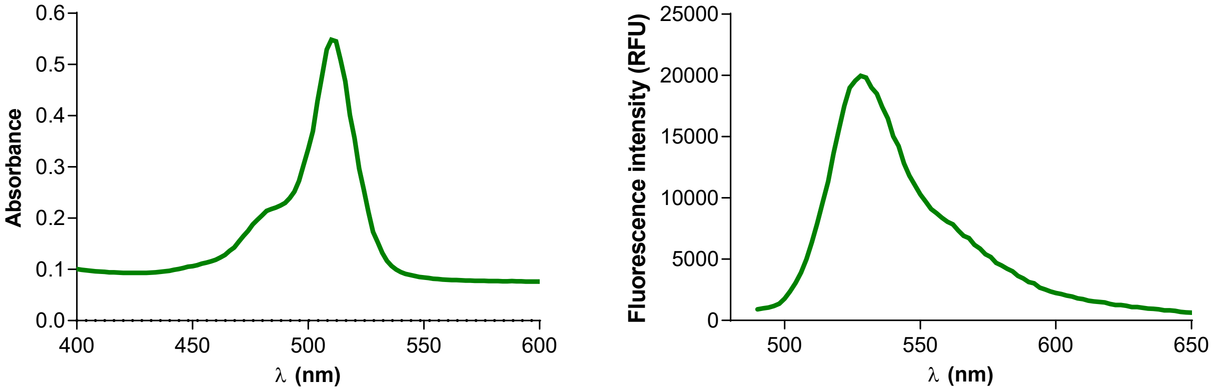

Supplementary Figure S4. Absorbance (left) and emission (right) spectra of amino acid **7** (20 μM) in EtOH. λ_{exc}: 460 nm (n=3).

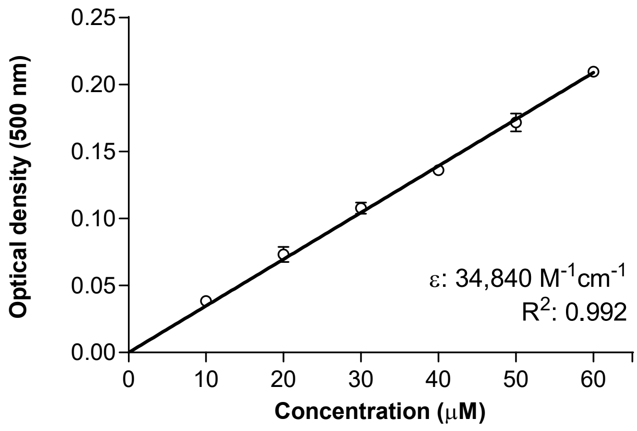

Supplementary Figure S5. Determination of the extinction coefficient of the amino acid 4. Solutions of the amino acid at different concentrations were prepared in ethanol and their optical densities were measured at 500 nm in a NanoDrop 1000 spectrophotometer. Data represented as means±SEM (n=6).

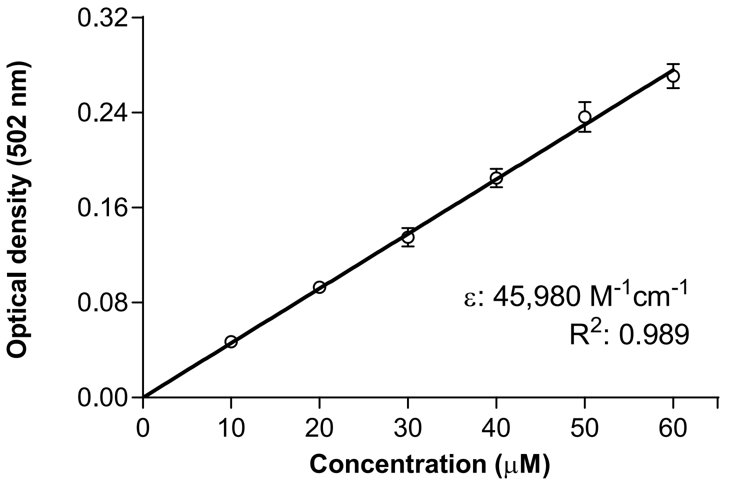

Supplementary Figure S6. Determination of the extinction coefficient of the amino acid 5. Solutions of the amino acid at different concentrations were prepared in ethanol and their optical densities were measured at 502 nm in a NanoDrop 1000 spectrophotometer. Data represented as means±SEM (n=6).

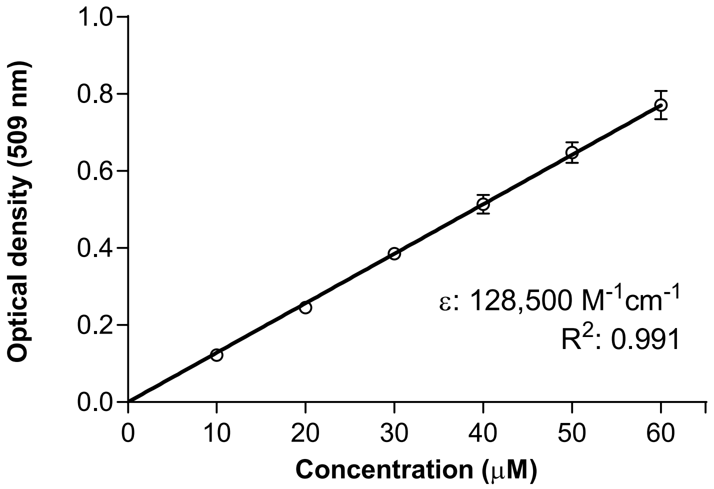

Supplementary Figure S7. Determination of the extinction coefficient of the amino acid 6. Solutions of the amino acid at different concentrations were prepared in ethanol and their optical densities were measured at 509 nm in a NanoDrop 1000 spectrophotometer. Data represented as means±SEM (n=6).

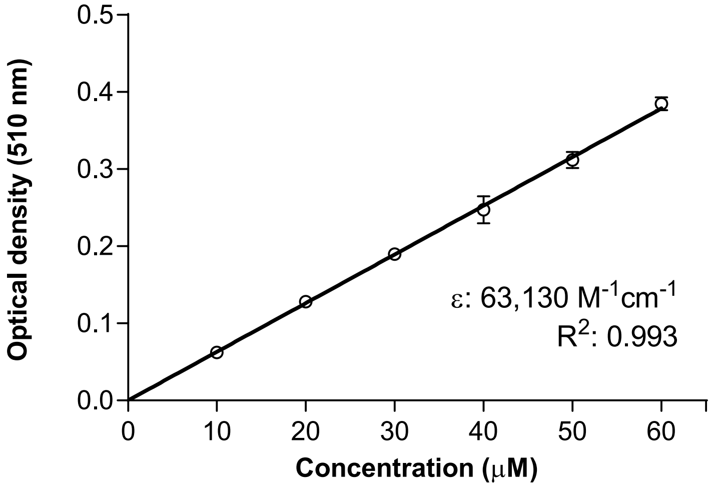

Supplementary Figure S8. Determination of the extinction coefficient of the amino acid 7. Solutions of the amino acid at different concentrations were prepared in ethanol and their optical densities were measured at 510 nm in a NanoDrop 1000 spectrophotometer. Data represented as means±SEM (n=6).

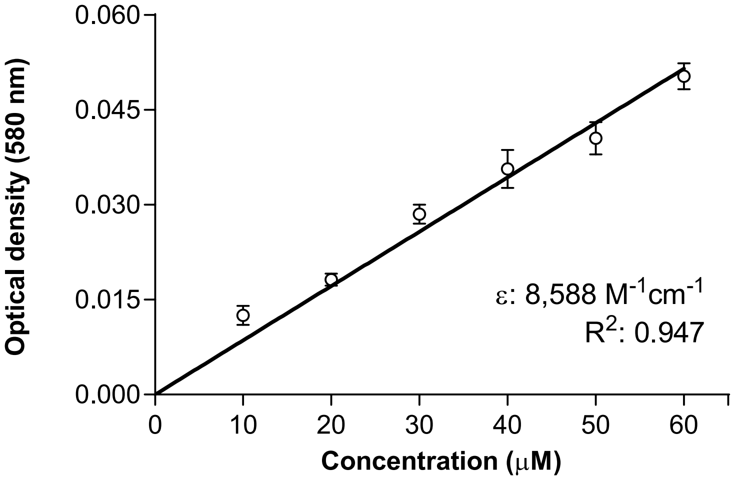

Supplementary Figure S9. Determination of the extinction coefficient of the amino acid 10. Solutions of the amino acid at different concentrations were prepared in ethanol and their optical densities were measured at 580 nm in a NanoDrop 1000 spectrophotometer. Data represented as means±SEM (*n*=6).

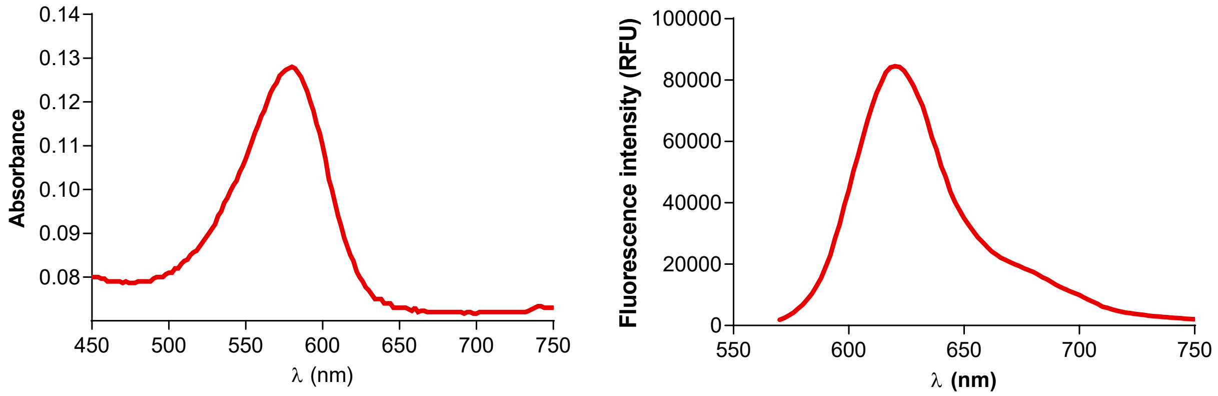

Supplementary Figure S10. Absorbance (left) and emission (right) spectra of amino acid **10** (20 μM) in EtOH. λ_{exc}: 540 nm (n=3).

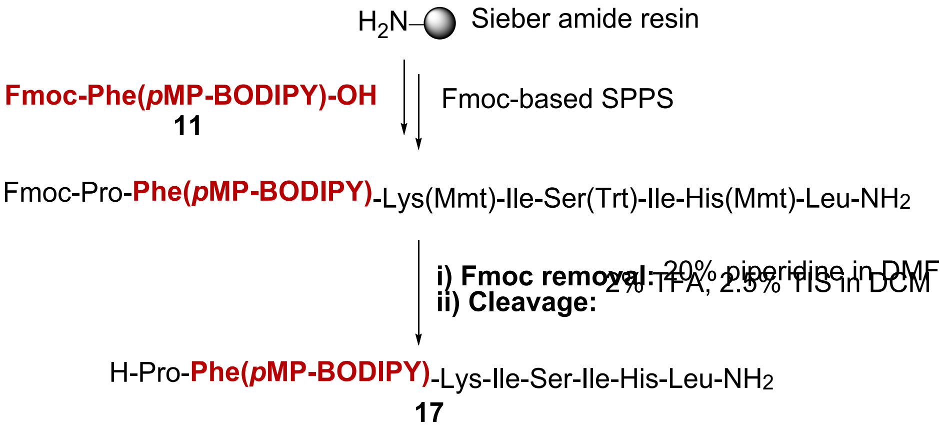

Supplementary Figure S11. Synthetic scheme for the fluorescent peptide **17**.

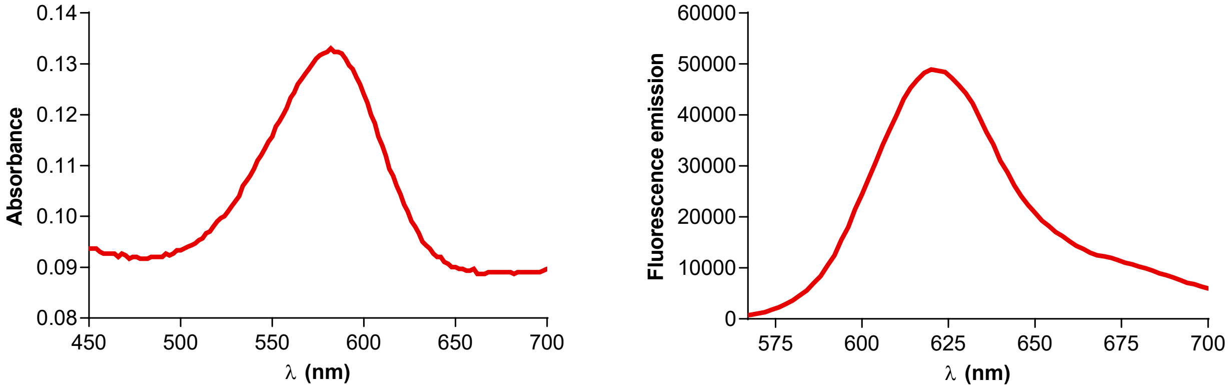

Supplementary Figure S12. Spectral characterization of compound 17. Absorbance (left) and emission (right) spectra of compound **17** (10 μM) in EtOH. λ_{exc}: 530 nm (n=3).

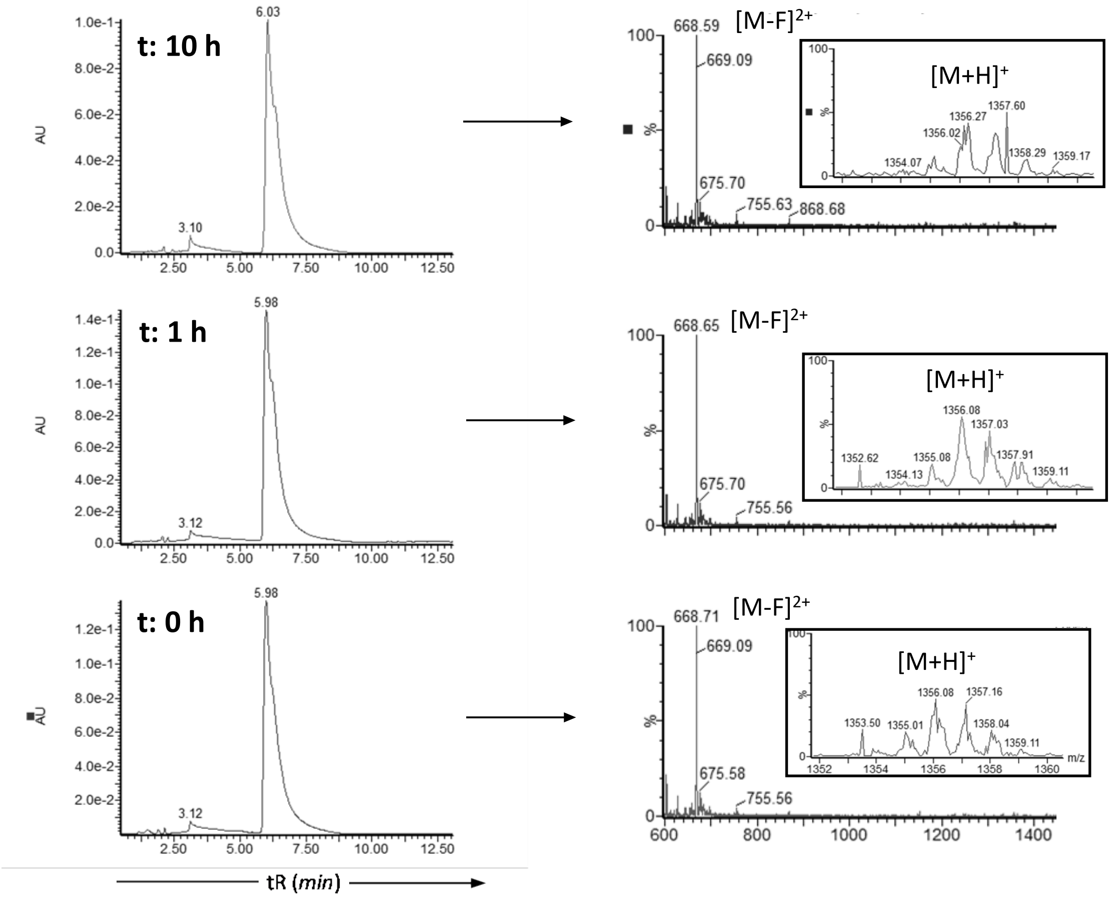

Supplementary Figure S13. HPLC-MS stability analysis of compound 17 in urine samples. HPLC traces of compound **17** (100 μM) monitored at 570 nm after incubation in diluted urine samples (urine: water, 1:6) for 10 h at 37 °C.

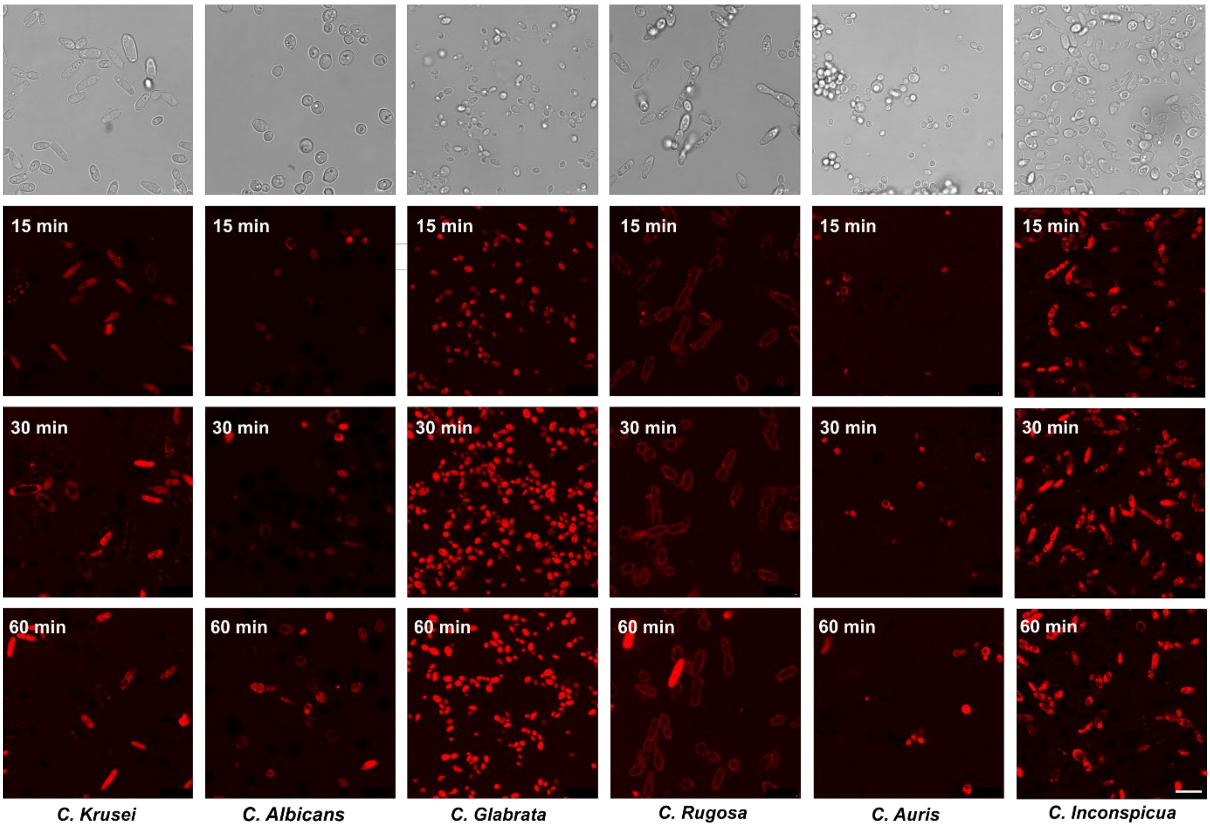

Supplementary Figure S14. Confocal microscopy of the interaction and internalization of peptide 17 by different *Candida* strains. Peptide **17** (10 µM) was added to different *Candida* strains (5×10^5 cells mL^{-1}) in PBS and incubated at 37 °C for 15 min and then imaged over the following 60 min without any washing or processing steps. λ_{exc}: 575 nm, λ_{em}: 600-650 nm. Scale bar: 10 µm.

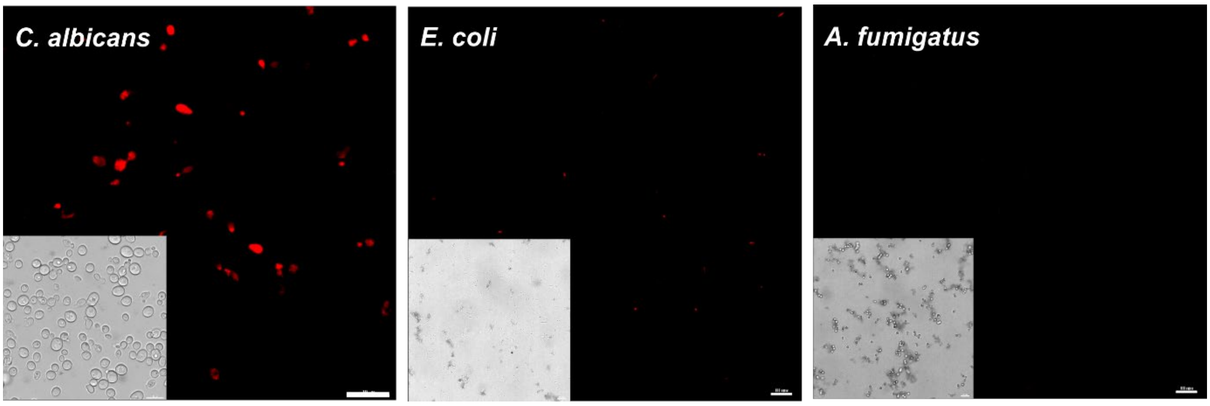

Supplementary Figure S15. Fluorescence live-cell confocal microscopy comparison of *C. albicans* and other bacterial and fungal strains after incubation with peptide 17. Brightfield (insets) and fluorescence images of *C. albicans* (5×10^6 cells mL^{-1}), *E. coli* (5×10^8 cells mL^{-1}) and *A. fumigatus* (5×10^6 cells mL^{-1}) were obtained after 1 h incubation with peptide **17** (10 μM) in PBS at 37 °C without any washings. λ_{exc}: 575 nm, λ_{em}: 600-650 nm. Scale bar: 10 μm.

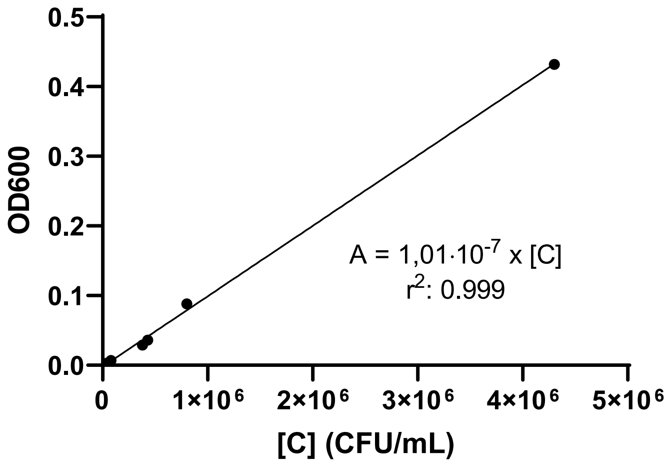

Supplementary Figure S16. Calibration curve for the determination of colony forming units (CFU). Data represented as means±SEM (n=3).

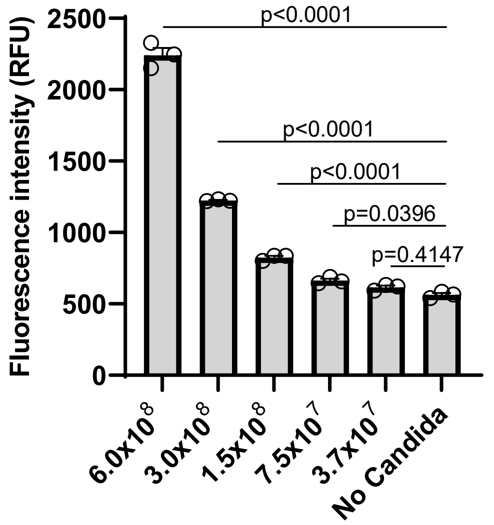

Supplementary Figure S17. Fluorescence intensity signals from GFP-*C. albicans* upon incubation (1 h, 37 °C) in urine samples with cell concentrations ranging from 10^7-10^8 CFU mL^{-1}. Data presented as means±SEM (n=3). P values obtained from ONE-ANOVA tests with multiple comparisons.

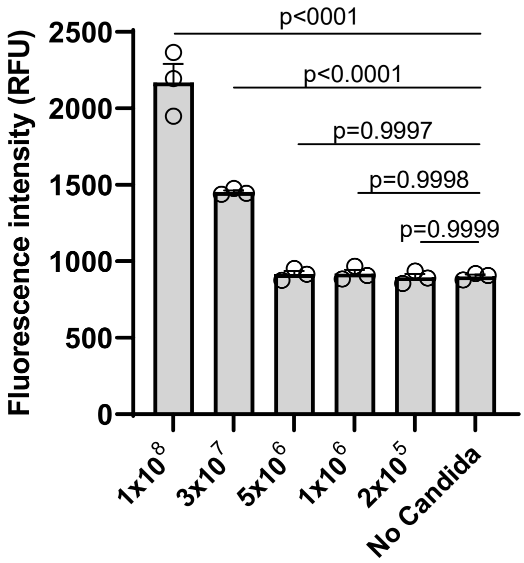

Supplementary Figure S18. Fluorescence intensity signals from peptide **18** upon incubation with *C. albicans* (1 h, 37 °C) in urine samples with cell concentrations ranging from 10^5-10^8 CFU mL^{-1}, with a LOD of ~7×10^6 CFU mL^{-1}. Data presented as means±SEM (n=3). P values obtained from ONE-ANOVA tests with multiple comparisons.

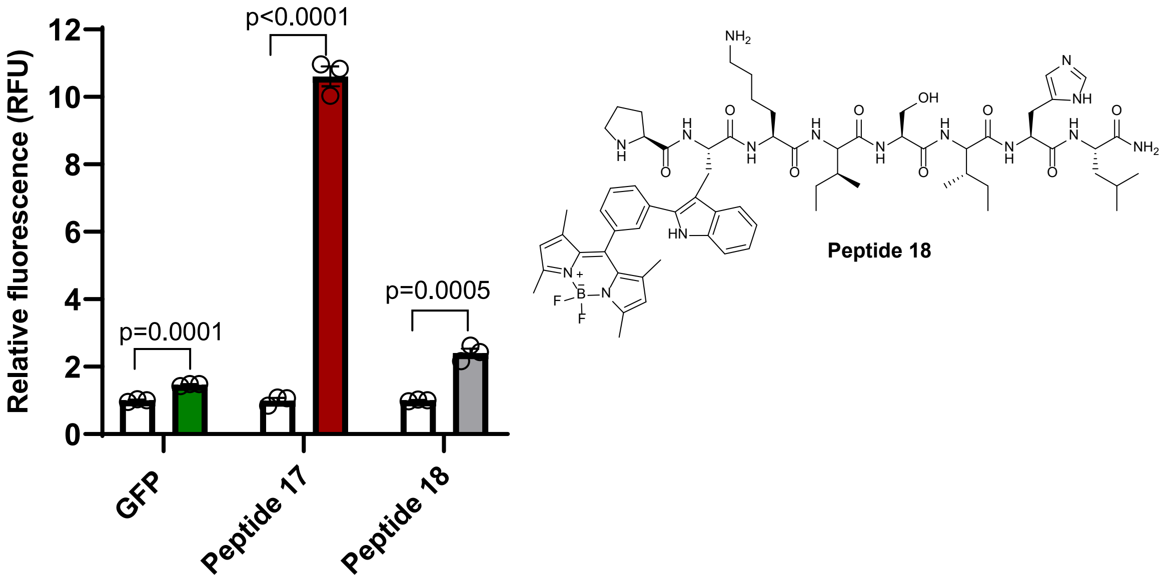

Supplementary Figure S19. Relative fluorescence intensity of GFP (green), peptide **17** (red) and peptide **18** (grey) upon incubation in urine samples with *C. albicans* (10^8 CFU mL^{-1}) or urine alone (white). λ_{exc}: 450 nm (GFP, peptide **18**) and 530 nm (peptide **17**), λ_{em}: 515 nm (GFP, peptide **18**) and 642 nm (peptide **17**). Data presented as means±SEM (n=3). P values were obtained from unpaired two-tailed t tests.

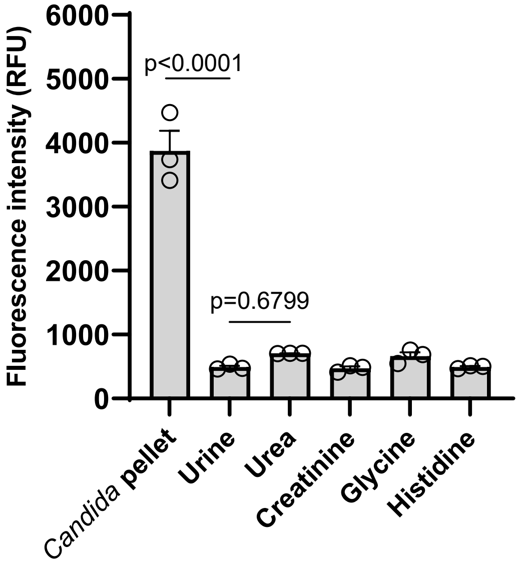

Supplementary Figure S20. Fluorescence intensity signals from peptide **17** upon incubation (1 h, 37 °C) in C. albicans (2 x 10^7 CFU mL^{-1}), urine, urea (3.3 mg mL^{-1}), creatinine (0.33 mg mL^{-1}), glycine (0.33 mg mL^{-1}) and histidine (0.98 mg mL^{-1}). Data presented as means±SEM (n=3). P values obtained from ONE-ANOVA tests with multiple comparisons.

NMR spectra

^{1}H-NMR and ^{13}C-NMR spectra of compound **1a**.

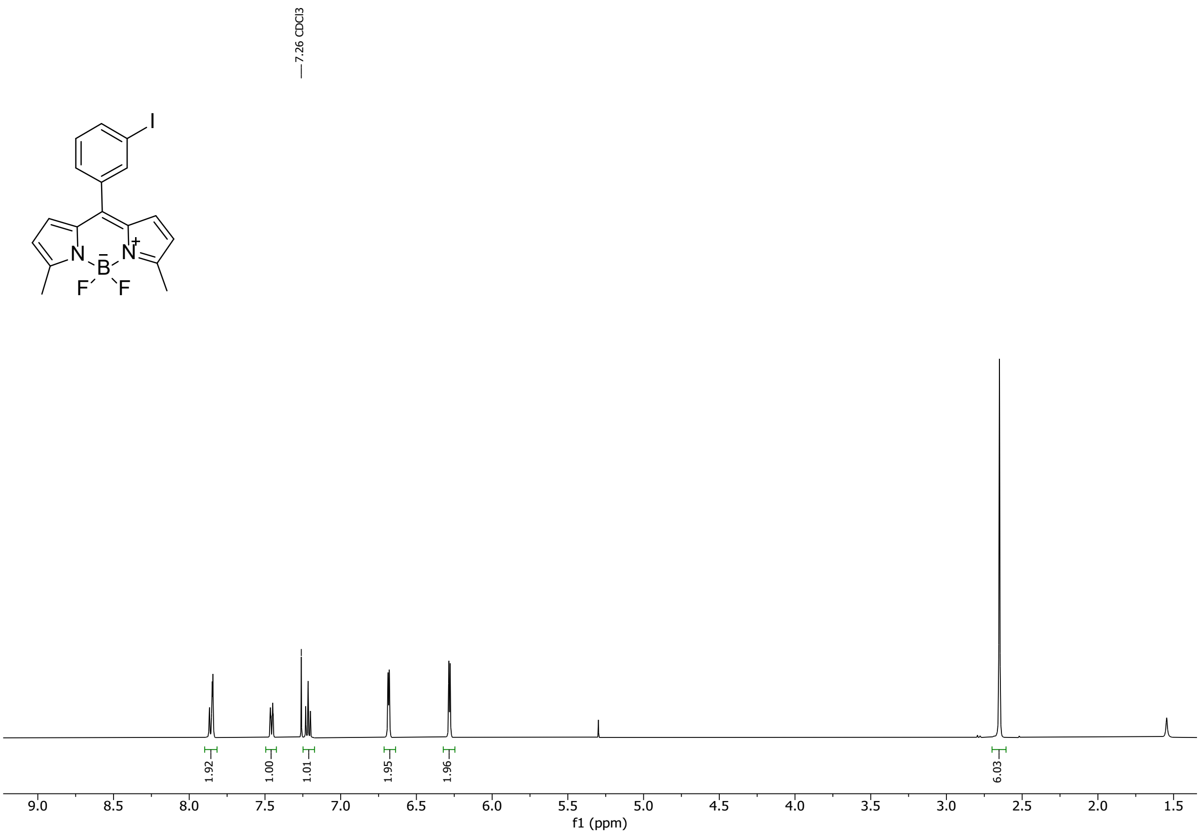

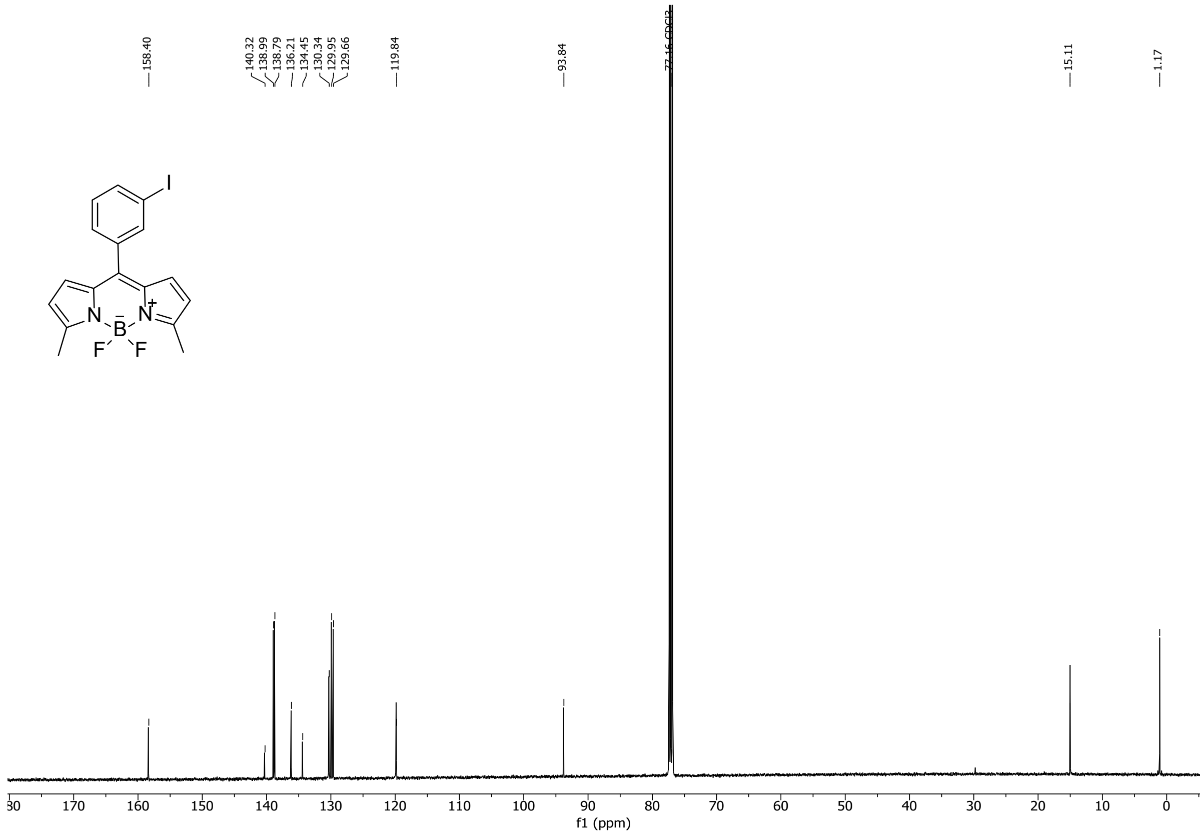

^{1}H-NMR and ^{13}C-NMR spectra of compound **4**.

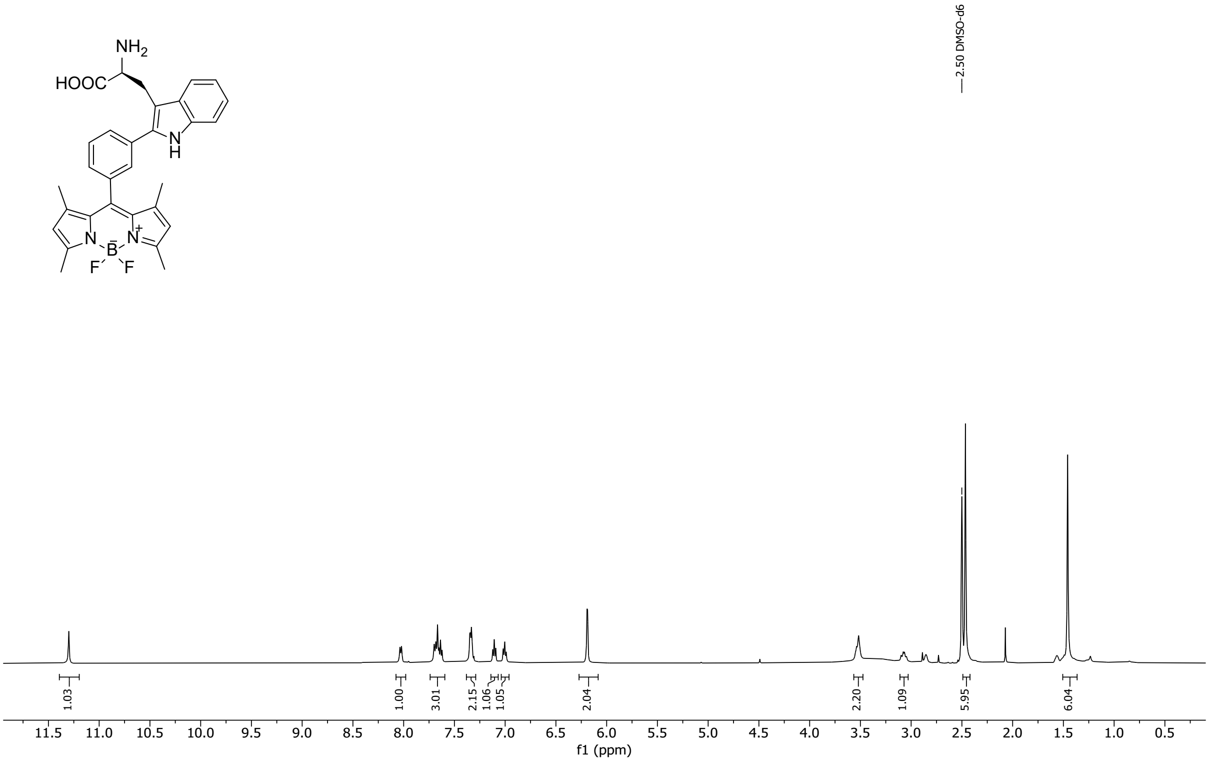

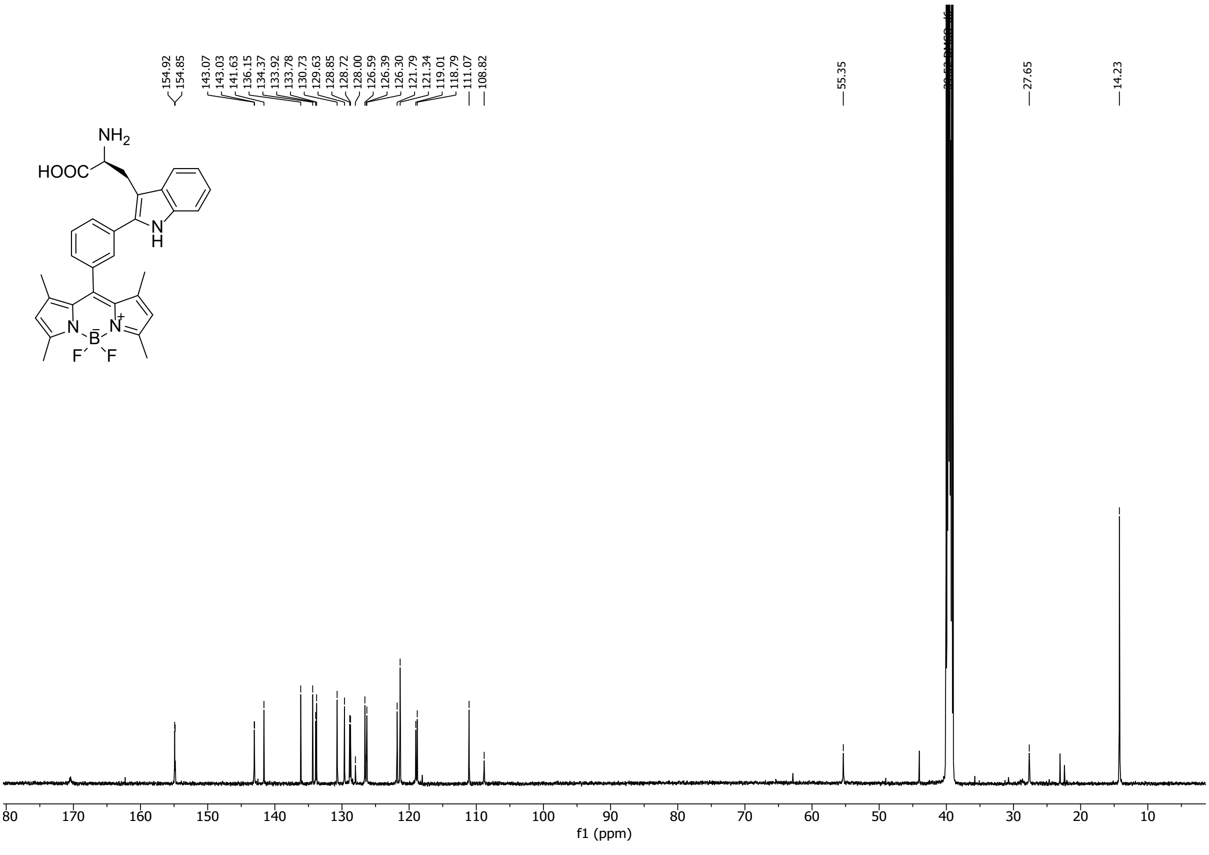

^{1}H-NMR and ^{13}C-NMR spectra of compound **6**.

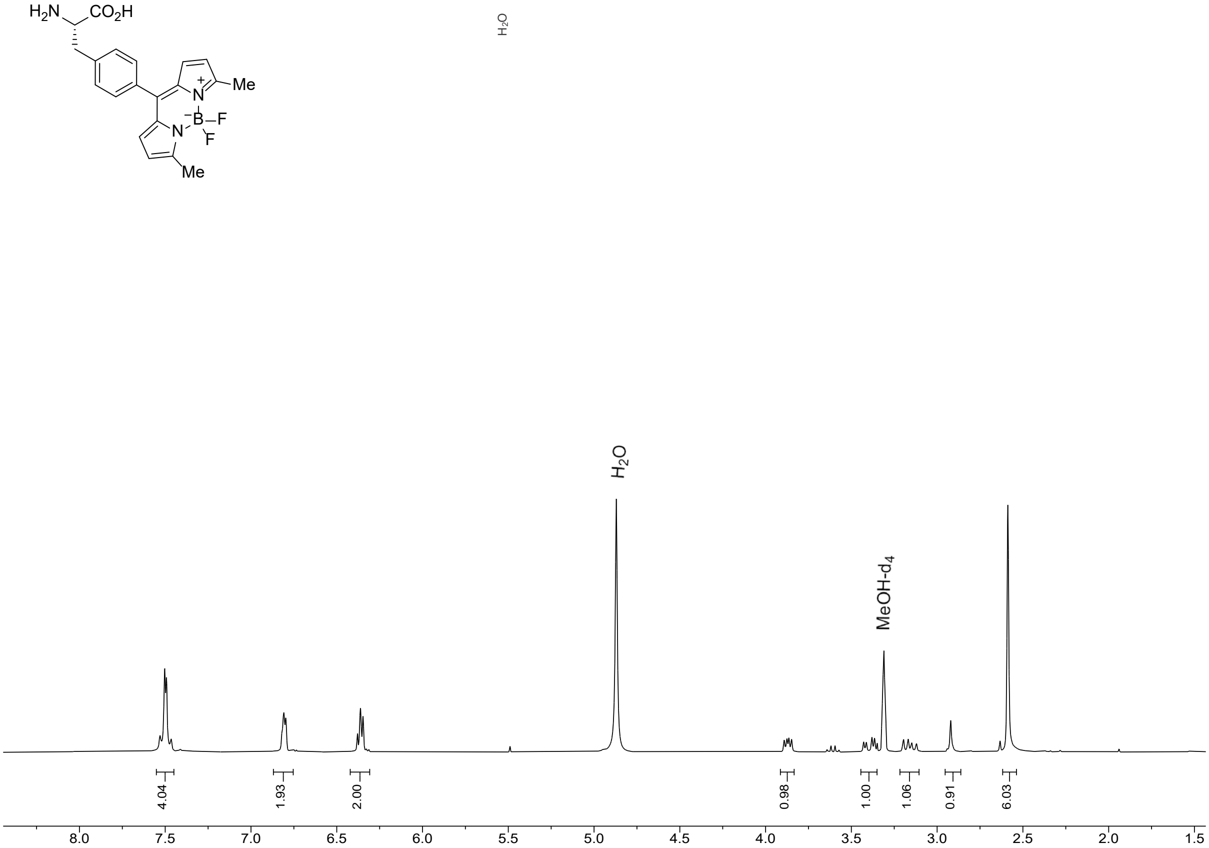

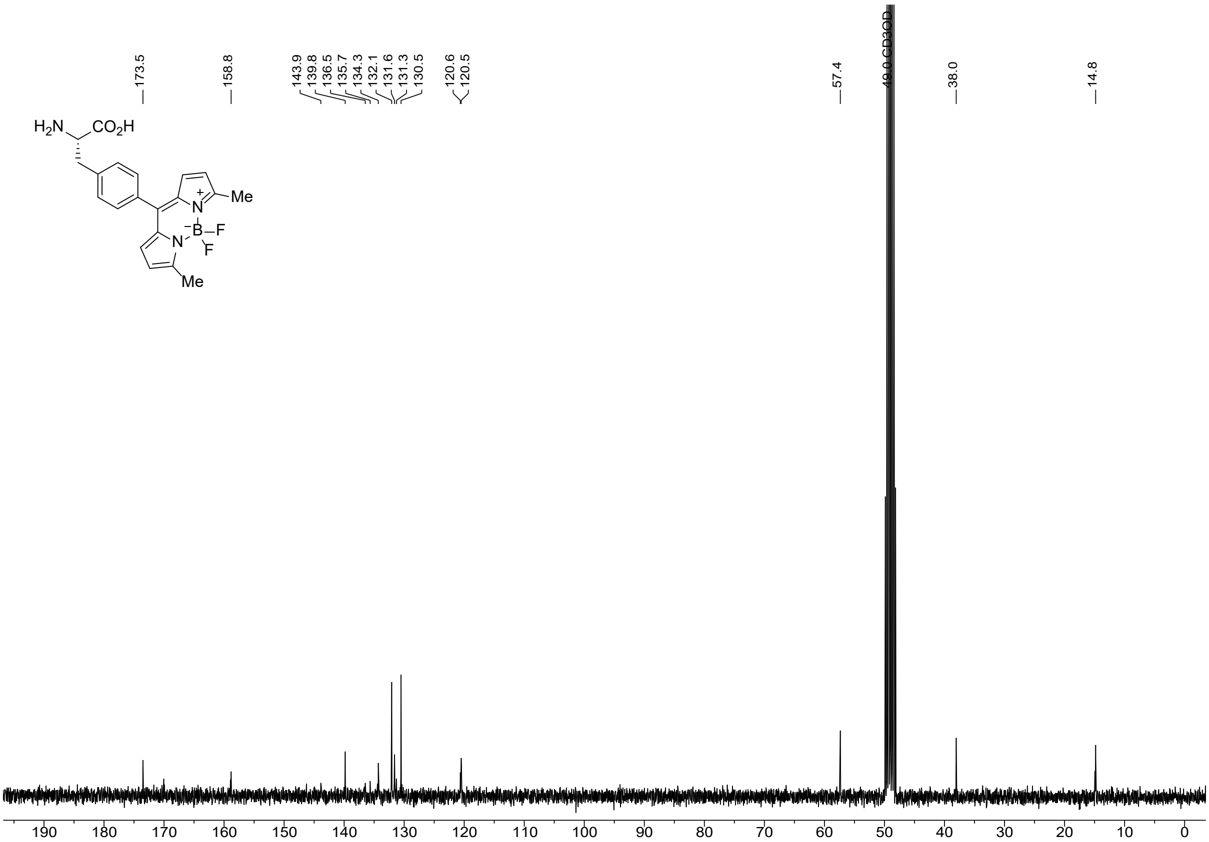

^{1}H-NMR and ^{13}C-NMR spectra of compound **Fmoc-7**.

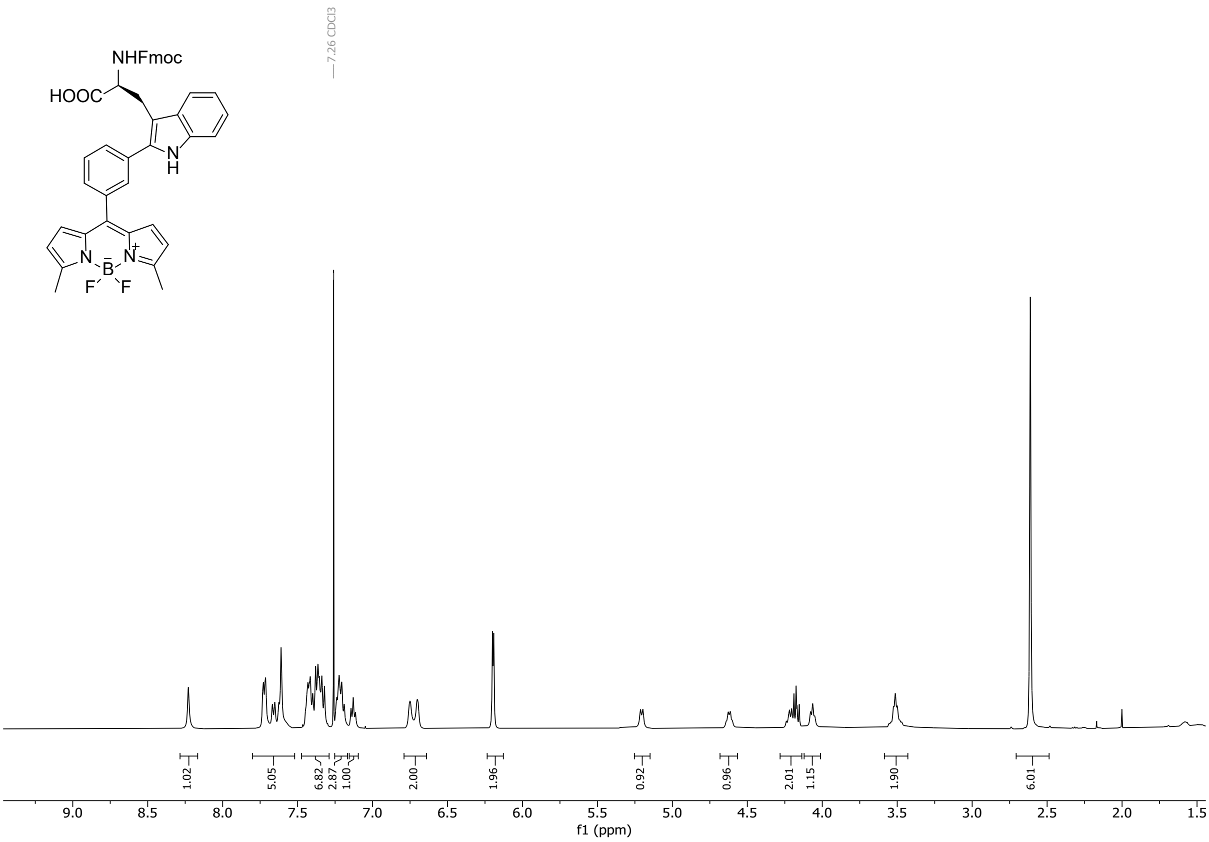

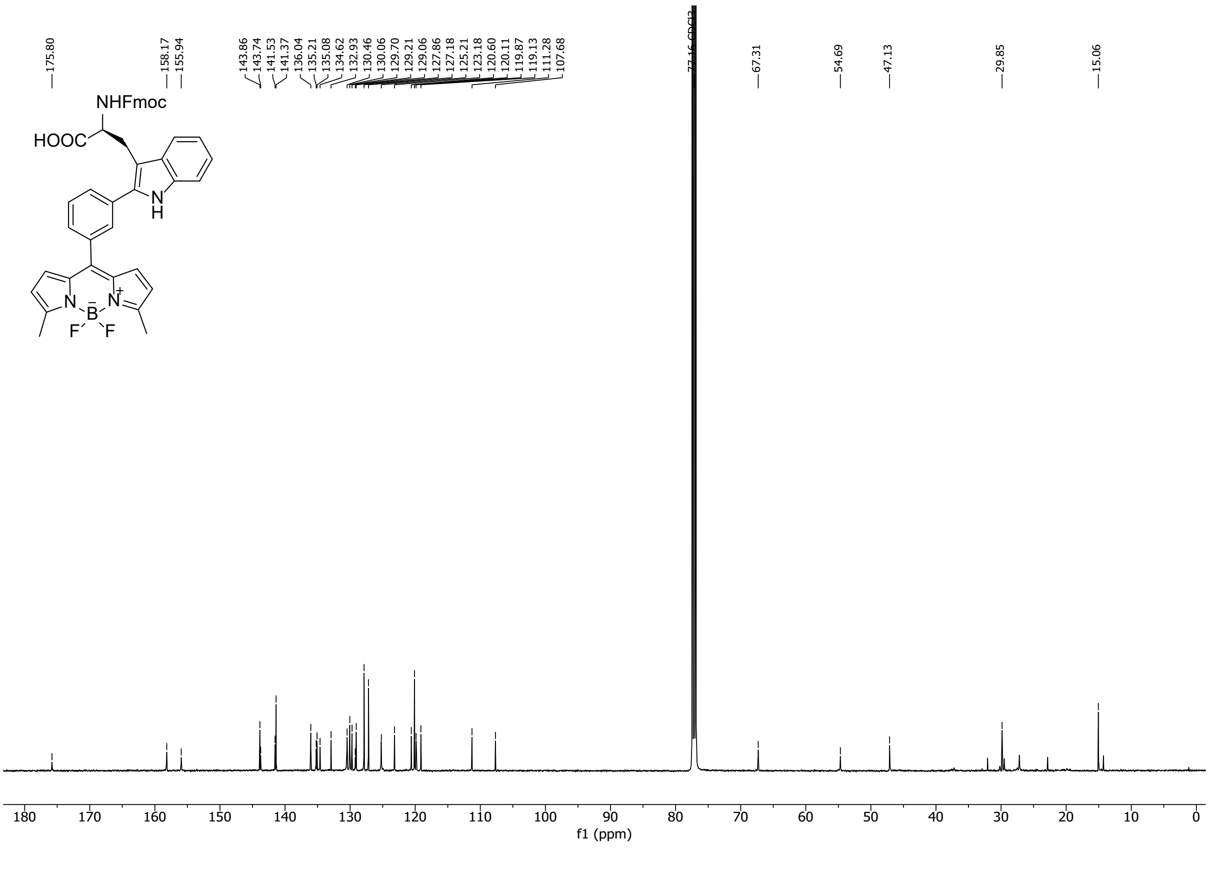

^{1}H-NMR and ^{13}C-NMR spectra of compound **7**.

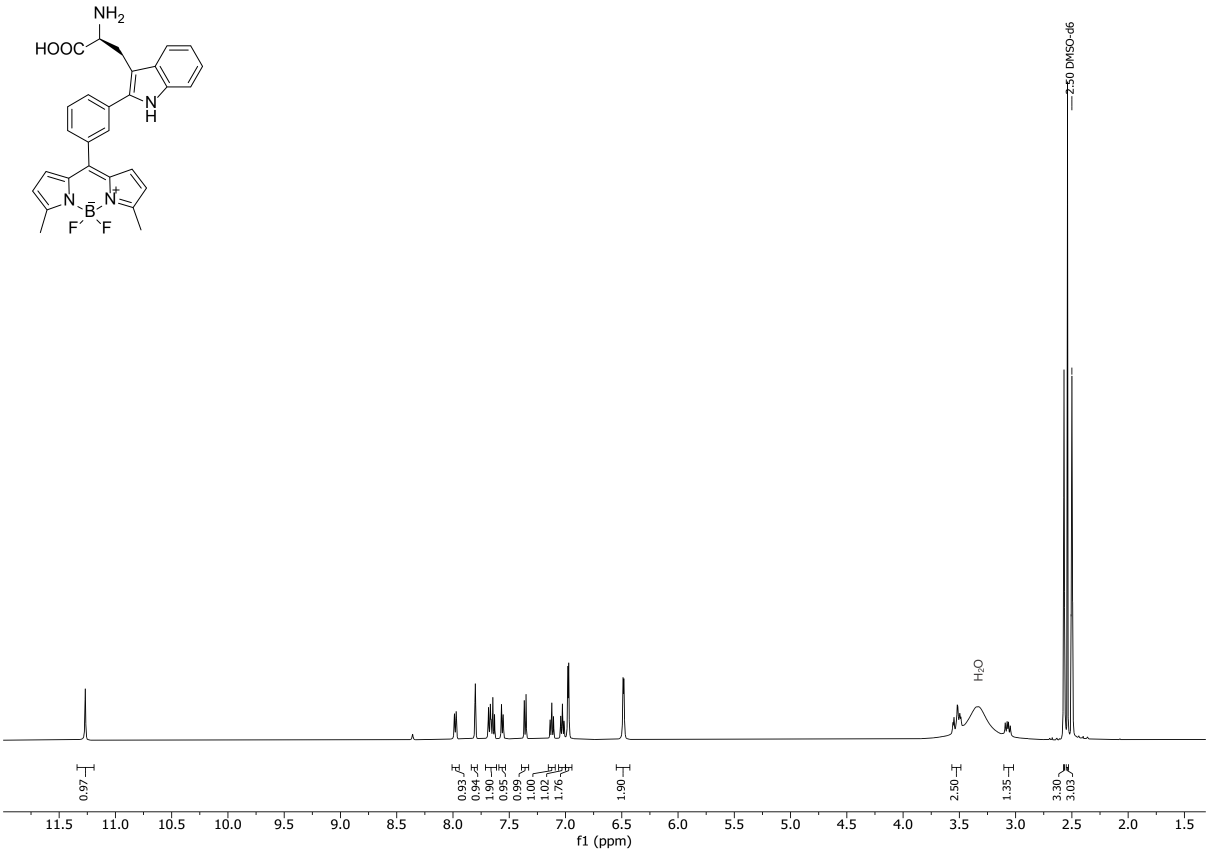

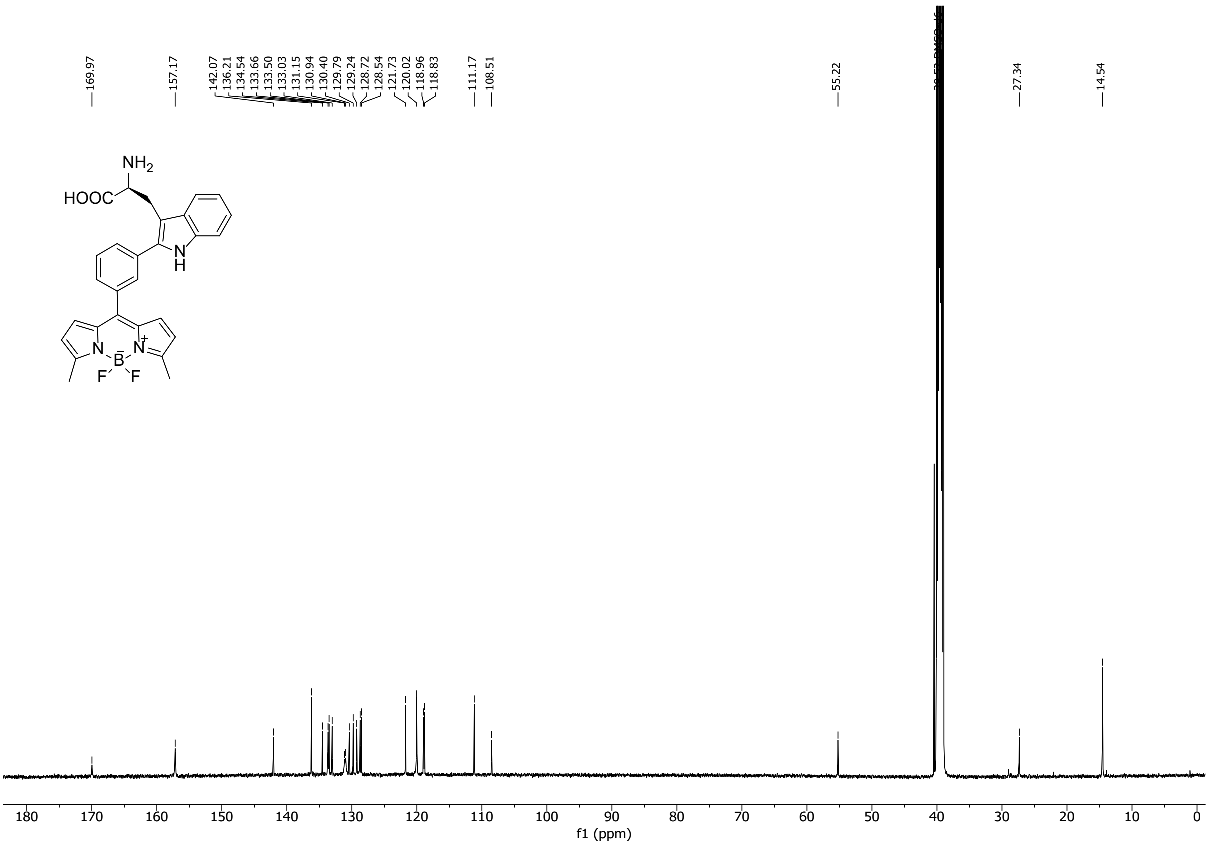

^{1}H-NMR and ^{13}C-NMR spectra of compound **11**.

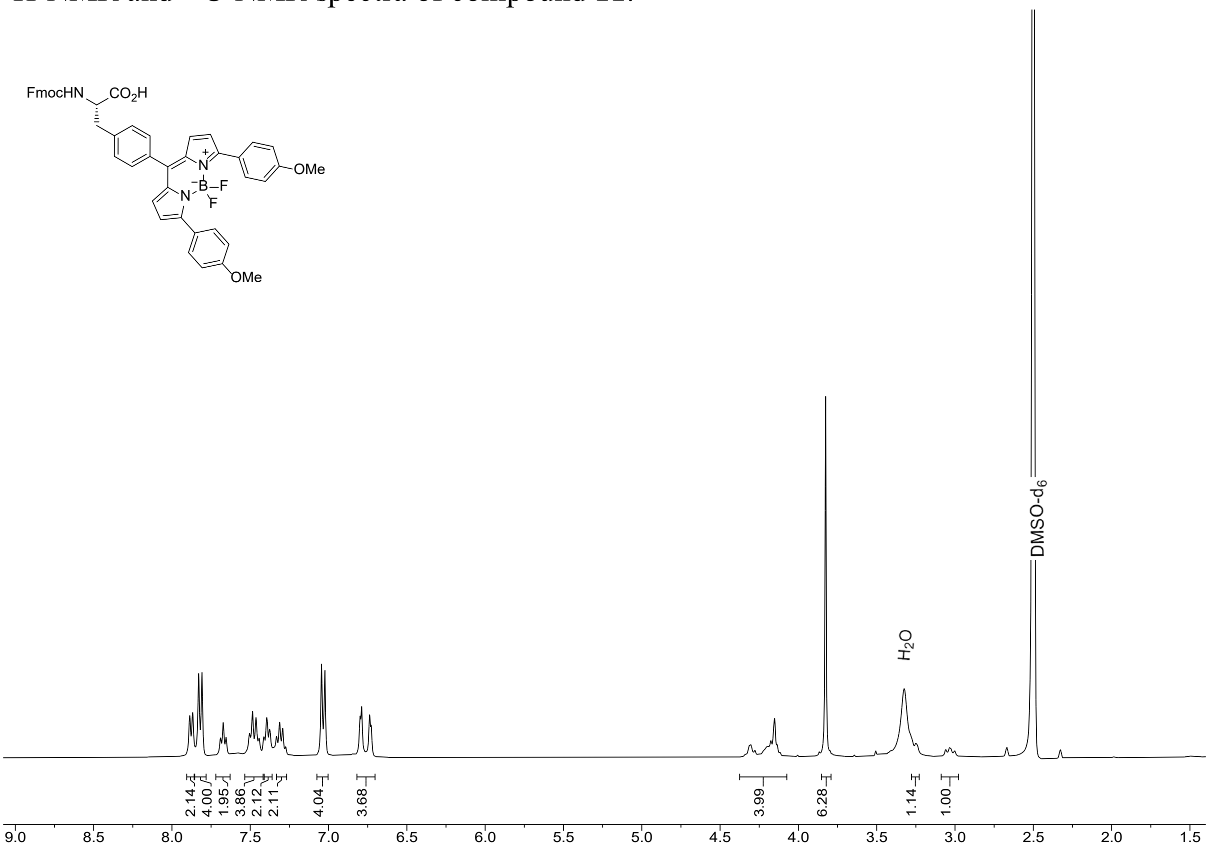

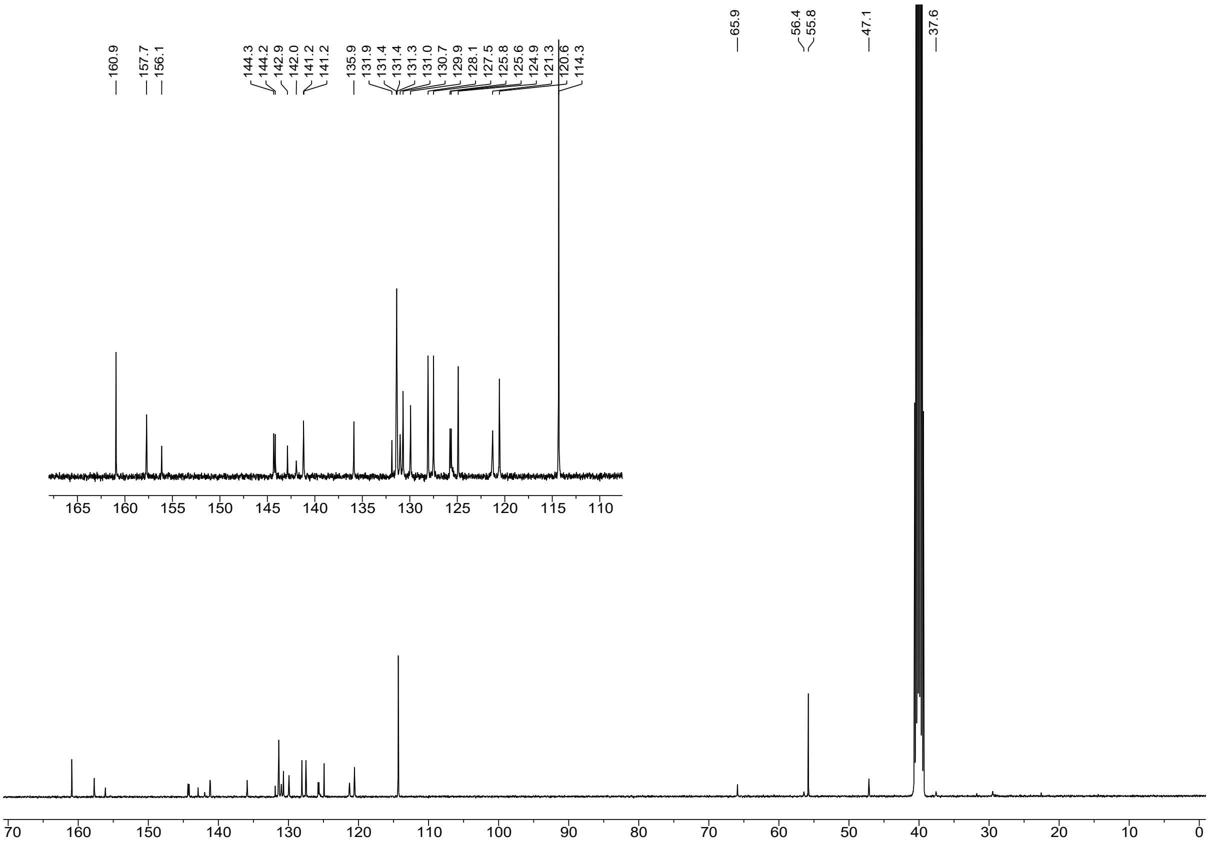

MALDI spectra

Peptide **12**.

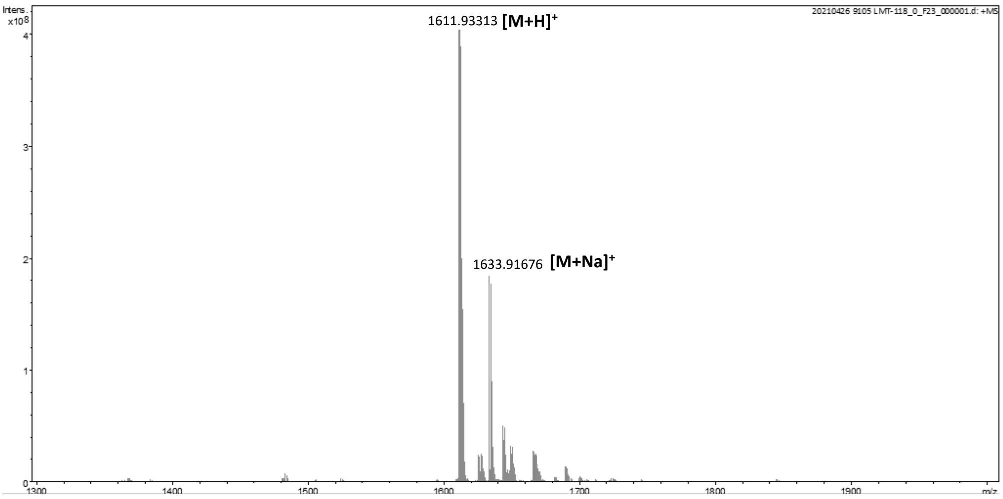

Peptide **13**.

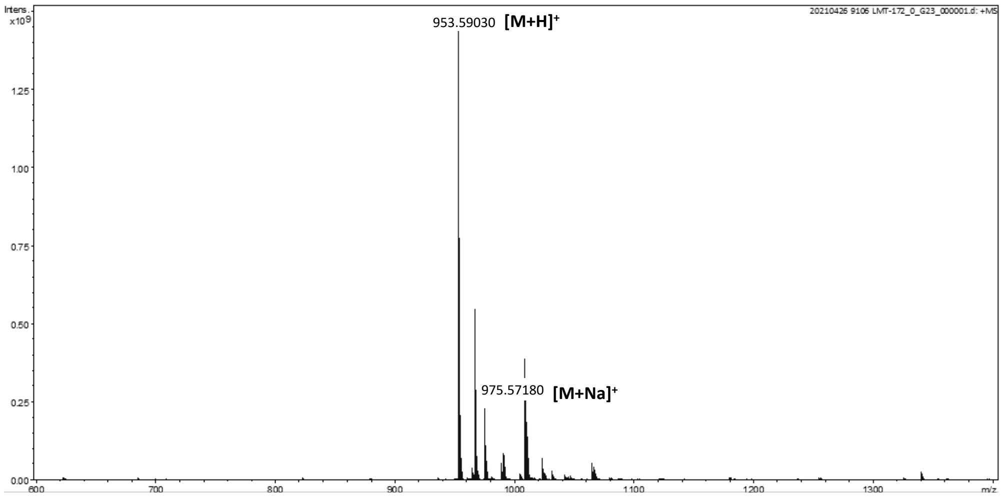

Peptide **14**.

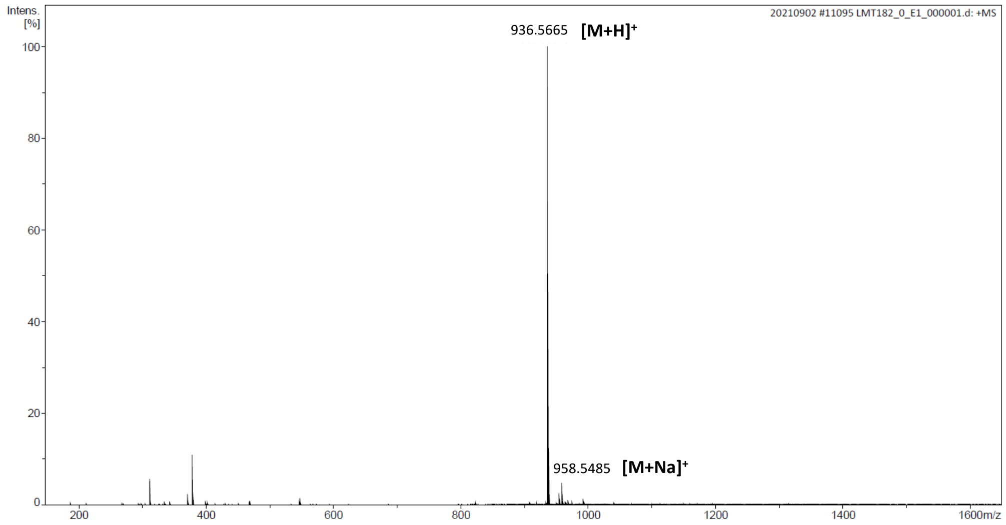

Peptide **15**.

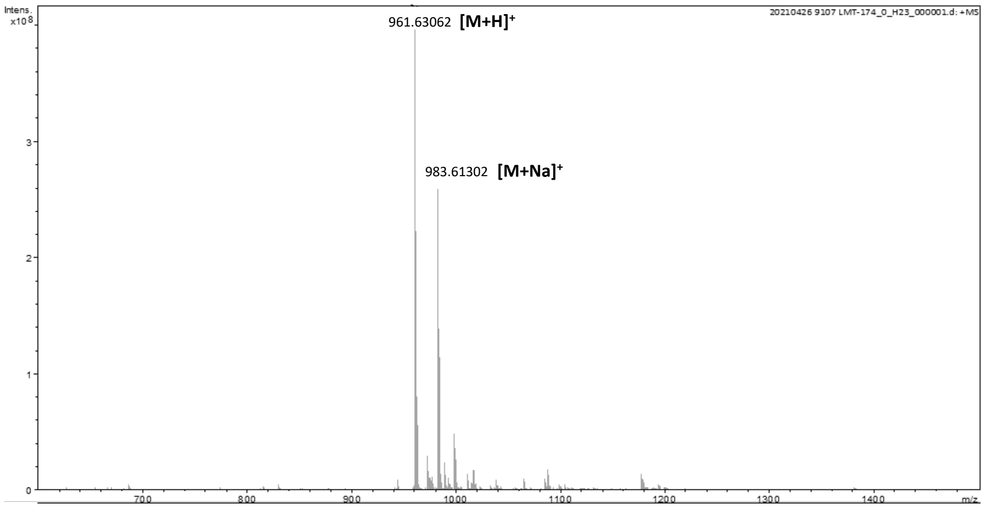

Peptide **16**.

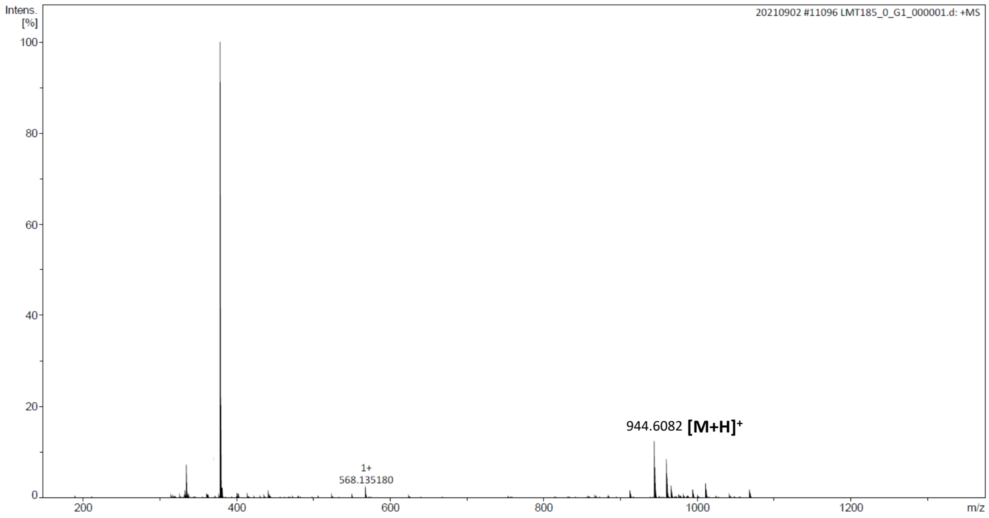

Peptide **17**.

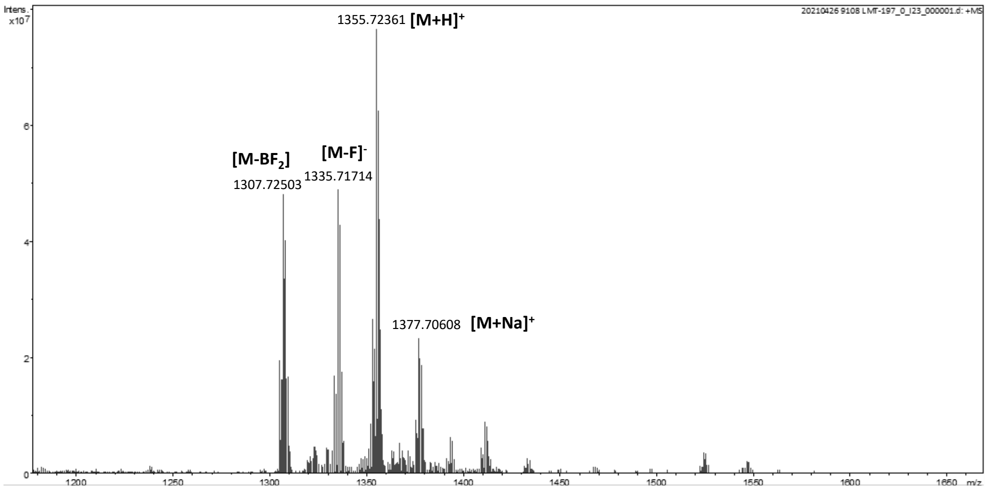

Supplementary Table S1. FC, M*, TS* and R* values for model systems I-II in kcal mol^{-1} or in eV (brackets). For MS-I, the R* conformation is a minimum, while in MS-II this is a transition state (Cs symmetry) whose imaginary normal mode of vibration breaks the symmetry into two lower energy conformations (R^1* and R^2*).

Compound	FC[a]	M*[b]	TS*[c]	R*/R^1*	R^2*	TS*-M*
**MS-I**	70.5 (3.06)	69.0 (2.99)	70.8 (3.07)	68.7 (2.98)	-	1.82 (0.079)
**MS-II**	71.5 (3.10)	70.1 (3.04)	73.6 (3.19)	67.0 (2.91)	62.5 (2.71)	3.57 (0.155)

Supplementary Table S2. Comparison between the TS*-M* energy barriers of model systems I and Phe-BODIPY amino acid 5 for different solvents (Kcal mol^{-1}).

Compound	Water (ε= 78.3)	Ethylene glycol (ε= 40.2)	BuOH (ε=16.8)	n-Octanol (ε= 9.9)
**MS-I**	4.04	4.00	3.87	3.73
**5**	8.25	8.22	8.13	8.02

Supplementary Table S3. Comparison between the TS*-M* energy barriers (kcal mol^{-1}) in gas phase and ethylene glycol of model systems I-II and Phe/Trp-BODIPY amino acids (4-7).

Compound	Gas	Ethylene glycol (ε= 40.2)
**7**	4.39	6.00
**6**	4.53	6.02
**4**	6.36	8.15
**5**	6.59	8.22

Supplementary Table S4. Optimised excited state geometries for FC, M* TS* and R* minima conformations of MS-I and MS-II and Phe/Trp-BODIPY amino acids (**4**-**7**).

	**FC**	**M***	**TS***	**R*/R1***	**R2***
**MS-I**	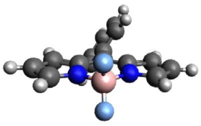	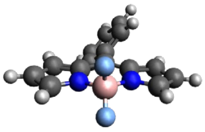	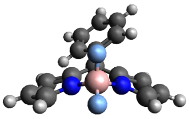	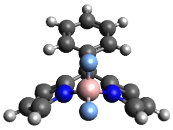	—
**MS-II**	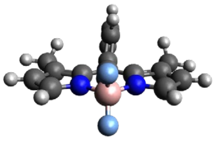	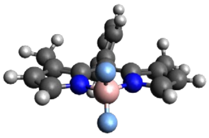	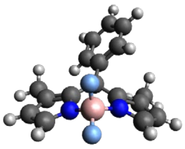	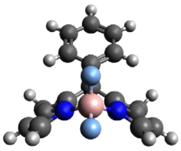	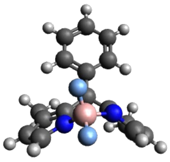
**4**	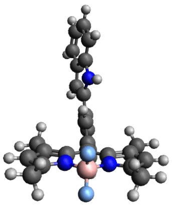	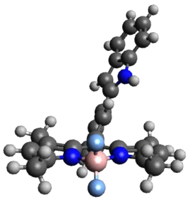	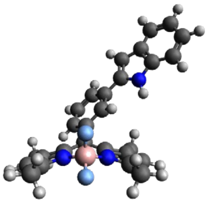	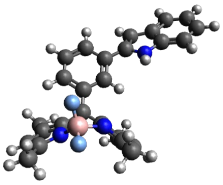	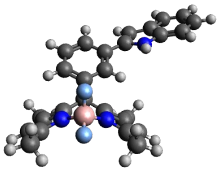
**5**	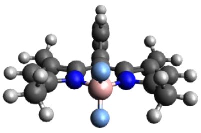	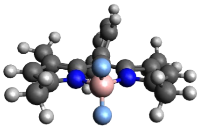	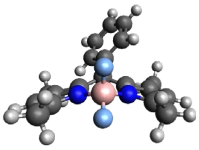	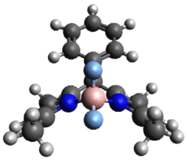	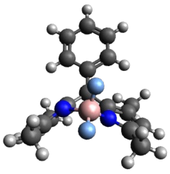
**6**	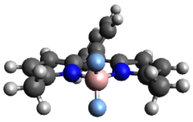	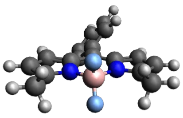	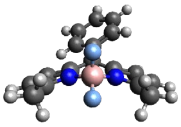	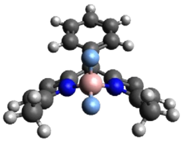	—
**7**	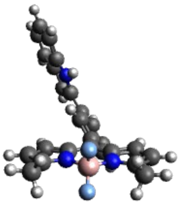	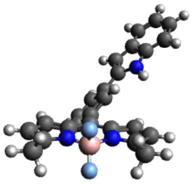	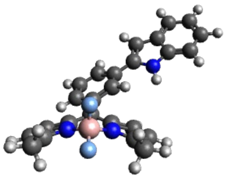	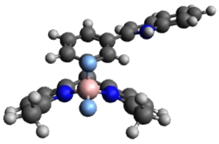	—

Supplementary Table S5. Optimised geometries in Cartesian coordinates.

MS-I

Ground state minimum S_0 (GS)

Atom	X	Y	Z
7	−1.662799	−1.237707	−0.043482
7	−1.662806	1.237704	0.043419
6	−0.277906	1.208962	0.057722
6	0.183068	2.539823	0.186411
6	0.419799	0.000004	−0.000008
6	−0.277899	−1.208957	−0.057758
6	0.183085	−2.539809	−0.186504
6	−0.933223	−3.352505	−0.235674
6	−0.933246	3.352515	0.235526
6	−2.052281	−2.504791	−0.148561
6	−2.052297	2.504790	0.148432
1	−0.961737	4.424792	0.332768
1	−3.102914	2.753288	0.165797
1	1.217288	2.837085	0.250758
1	2.054651	−1.316132	1.686996
6	2.601428	−0.749063	0.943717
6	1.896923	0.000004	0.000005
6	2.601449	0.749068	−0.943693
1	2.054689	1.316137	−1.686985
6	3.988340	0.741674	−0.946186
1	4.526588	1.313663	−1.691147
6	4.683859	0.000000	0.000033
1	5.766455	−0.000002	0.000044
6	3.988319	−0.741672	0.946237
1	4.526550	−1.313664	1.691208
5	−2.612847	−0.000008	0.000054
9	−3.389404	0.034702	−1.135001
1	−3.102896	−2.753294	−0.165952
1	−0.961707	−4.424777	−0.332971
1	1.217308	−2.837060	−0.250855
9	−3.389186	−0.034727	1.135258

Excited state minimum S_1 (M*)

Atom	X	Y	Z
7	−1.667795	−1.228967	−0.077322
7	−1.691117	1.211433	−0.000264
6	−0.284185	1.219069	0.024322
6	0.135029	2.569837	0.091987
6	0.460932	0.014436	−0.007430
6	−0.262653	−1.201245	−0.102550
6	0.188810	−2.526689	−0.309327
6	−0.935552	−3.329531	−0.396312
6	−1.008054	3.350609	0.090671
6	−2.064033	−2.485411	−0.250171
6	−2.115972	2.469994	0.036271
1	−1.066533	4.425876	0.138573
1	−3.172329	2.693085	0.044592
1	1.157075	2.902946	0.153625
1	2.065484	−1.516265	1.517164
6	2.621928	−0.859043	0.859821
6	1.924309	0.015049	0.015950
6	2.660254	0.879073	−0.804912
1	2.137048	1.533975	−1.490727
6	4.045805	0.868507	−0.779509
1	4.597894	1.533086	−1.432154
6	4.725504	0.002011	0.067609
1	5.807685	−0.002166	0.088254
6	4.007044	−0.860726	0.886892
1	4.528300	−1.532358	1.557385
5	−2.622407	−0.023337	0.108046
9	−3.572304	−0.003949	−0.890048
1	−3.114915	−2.731678	−0.274721
1	−0.969314	−4.394821	−0.557296
1	1.219509	−2.822261	−0.411825
9	−3.223304	−0.063344	1.348951

Excited TS S_1 (TS*, 64.69i cm^{-1})

Atom	X	Y	Z
7	−1.601971	−1.260939	−0.081265
7	−1.758249	1.136801	0.071751
6	−0.390984	1.270542	−0.239022
6	−0.195804	2.607182	−0.660746
6	0.456481	0.113057	−0.152740
6	−0.241793	−1.118108	−0.376781
6	0.179635	−2.301890	−1.022528
6	−0.918973	−3.142702	−1.088015
6	−1.420386	3.250105	−0.580661
6	−2.006722	−2.453311	−0.498726
6	−2.362419	2.299762	−0.128790
1	−1.627923	4.283788	−0.804784
1	−3.419059	2.408184	0.062202
1	0.720162	3.051536	−1.001428
1	1.883854	−1.829977	0.961842
6	2.504695	−1.013192	0.616928
6	1.893784	0.106877	0.015717
6	2.728094	1.173184	−0.363930
1	2.316898	2.038595	−0.855962
6	4.096716	1.113704	−0.163772
1	4.715438	1.944256	−0.479524
6	4.678824	0.000401	0.428512
1	5.748672	−0.038212	0.587114
6	3.870143	−1.061421	0.821652
1	4.306546	−1.926826	1.304216
5	−2.407272	−0.154495	0.644361
9	−3.734497	−0.213886	0.297910
1	−3.031741	−2.763938	−0.366029
1	−0.958552	−4.137638	−1.500785
1	1.171830	−2.476254	−1.405406
9	−2.209500	−0.228027	2.002174

Excited rotated minimum S_1 (R*)

Atom	X	Y	Z
7	−0.098233	−1.652728	1.205050
7	−0.098233	−1.652728	−1.205050
6	0.373194	−0.337266	−1.175727
6	1.140068	−0.132149	−2.341231
6	0.160445	0.471870	0.000000
6	0.373194	−0.337266	1.175727
6	1.140068	−0.132149	2.341231
6	1.085827	−1.307951	3.074332
6	1.085827	−1.307951	−3.074332
6	0.319036	−2.229049	2.325412
6	0.319036	−2.229049	−2.325412
1	1.530025	−1.498834	−4.037655
1	0.065103	−3.254982	−2.543207
1	1.676346	0.770627	−2.583019
1	−0.141523	2.072897	2.150692
6	−0.160815	2.604481	1.209156
6	−0.037819	1.882988	0.000000
6	−0.160815	2.604481	−1.209156
1	−0.141523	2.072897	−2.150692
6	−0.345701	3.972423	−1.202422
1	−0.442013	4.502307	−2.141685
6	−0.424987	4.670136	0.000000
1	−0.568750	5.742761	0.000000
6	−0.345701	3.972423	1.202422
1	−0.442013	4.502307	2.141685
5	−0.871462	−2.274011	0.000000
9	−0.735936	−3.638089	0.000000
1	0.065103	−3.254982	2.543207
1	1.530025	−1.498834	4.037655
1	1.676346	0.770627	2.583019
9	−2.175141	−1.852396	0.000000

MS-II

Ground state minimum S_0 (GS)

Atom	X	Y	Z
7	−1.849654	−1.236100	0.000038
7	−1.849757	1.235982	−0.000089
6	−0.453674	1.215485	0.002239
6	0.006403	2.566809	0.029206
6	0.230675	0.000029	0.000026
6	−0.453577	−1.215474	−0.002239
6	0.006639	−2.566738	−0.029305
6	−1.130046	−3.357534	−0.040215
6	−1.130363	3.357486	0.040173
6	−2.242078	−2.501029	−0.022031
6	−2.242306	2.500871	0.021920
1	−1.161222	4.434952	0.063329
1	−3.294928	2.741946	0.027317
1	1.862416	−0.262898	2.121427
6	2.412942	−0.147950	1.194797
6	1.715937	0.000048	0.000027
6	2.412952	0.148029	−1.194739
1	1.862435	0.262960	−2.121376
6	3.801133	0.144405	−1.193949
1	4.340003	0.255738	−2.126375
6	4.496549	0.000075	0.000042
1	5.579092	0.000091	0.000048
6	3.801123	−0.144285	1.194024
1	4.339984	−0.255606	2.126455
5	−2.790213	−0.000101	0.000038
9	−3.571234	−0.001681	−1.134942
1	−3.294676	−2.742208	−0.027440
1	−1.160793	−4.435004	−0.063363
9	−3.571075	0.001410	1.135130
6	1.410417	3.084177	0.043061
1	2.007176	2.625712	0.832177
1	1.919975	2.882915	−0.900614
1	1.397747	4.162267	0.199688
6	1.410717	−3.083924	−0.043209
1	2.007383	−2.625349	−0.832336
1	1.920285	−2.882615	0.900449
1	1.398189	−4.162011	−0.199870

Excited state minimum S_1 (M*)

Atom	X	Y	Z
7	−1.851203	−1.224451	0.032367
7	−1.856236	1.217180	−0.050920
6	−0.451875	1.216516	−0.002616
6	−0.014558	2.582344	0.062274
6	0.272464	0.001585	0.001274
6	−0.446924	−1.216609	−0.008382
6	−0.003021	−2.579123	−0.095018
6	−1.150276	−3.353591	−0.084176
6	−1.165129	3.351318	0.028965
6	−2.263492	−2.487030	−0.009908
6	−2.274074	2.478573	−0.034935
1	−1.216720	4.428224	0.069429
1	−3.329711	2.706213	−0.045229
1	1.901333	−0.736667	2.013408
6	2.452419	−0.416489	1.136499
6	1.749284	0.003161	0.006745
6	2.460357	0.422053	−1.118395
1	1.915404	0.741669	−1.999345
6	3.847582	0.418157	−1.114459
1	4.389218	0.737071	−1.996174
6	4.540645	0.002200	0.016124
1	5.623170	0.001849	0.019757
6	3.839725	−0.413391	1.141904
1	4.375173	−0.732901	2.027171
5	−2.802098	−0.004724	0.020950
9	−3.628246	−0.047748	−1.084549
1	−3.318128	−2.719407	−0.007240
1	−1.196777	−4.429742	−0.145331
9	−3.556013	0.037145	1.177420
6	1.377830	3.112334	0.182753
1	1.961214	2.559601	0.920843
1	1.923638	3.052374	−0.761791
1	1.339863	4.159213	0.483861
6	1.392606	−3.100305	−0.216315
1	1.975650	−2.536942	−0.946696
1	1.934638	−3.046593	0.730756
1	1.361562	−4.144224	−0.528208

Excited TS S_1 (TS*, 48.31i cm^{-1})

Atom	X	Y	Z
7	−1.563305	−1.460947	0.108784
7	−1.969421	0.922982	0.089037
6	−0.620906	1.234706	−0.201653
6	−0.589544	2.626234	−0.563010
6	0.310586	0.171336	−0.092397
6	−0.297780	−1.131700	−0.342449
6	0.198051	−2.215466	−1.122711
6	−0.783104	−3.196350	−1.075461
6	−1.890028	3.084727	−0.464871
6	−1.865273	−2.683047	−0.330134
6	−2.706305	2.011129	−0.057147
1	−2.223484	4.092098	−0.659787
1	−3.764280	1.987003	0.157011
1	1.504921	−1.569684	1.459852
6	2.205205	−0.836690	1.075943
6	1.729635	0.184301	0.229226
6	2.661448	1.113115	−0.254909
1	2.353218	1.862325	−0.964143
6	3.996851	1.038429	0.108736
1	4.697522	1.759426	−0.293744
6	4.442524	0.042069	0.967118
1	5.485642	−0.009931	1.250700
6	3.534834	−0.897126	1.447673
1	3.866272	−1.679584	2.118749
5	−2.450591	−0.390627	0.785498
9	−3.777968	−0.607557	0.507381
1	−2.831110	−3.117804	−0.122709
1	−0.742572	−4.172201	−1.533222
9	−2.195186	−0.322784	2.134775
6	0.548968	3.485437	−1.009217
1	1.316446	3.580278	−0.240328
1	0.173129	4.483352	−1.232632
1	1.018173	3.099867	−1.916866
6	1.511253	−2.264609	−1.828315
1	1.498314	−3.036840	−2.596928
1	2.331770	−2.479865	−1.137952
1	1.737964	−1.305448	−2.298163

Excited TS S_1 (R1*, 114.89i cm^{-1})

Atom	X	Y	Z
7	−0.330318	−1.723296	1.209463
7	−0.330318	−1.723296	−1.209463
6	0.245824	−0.455921	−1.159549
6	1.125822	−0.319912	−2.263327
6	0.003687	0.373176	0.000000
6	0.245824	−0.455921	1.159549
6	1.125822	−0.319912	2.263327
6	1.007765	−1.498712	2.991135
6	1.007765	−1.498712	−2.991135
6	0.116888	−2.341475	2.297488
6	0.116888	−2.341475	−2.297488
1	1.511989	−1.740230	−3.914024
1	−0.198499	−3.348356	−2.524707
1	−0.429925	1.920419	2.149511
6	−0.511899	2.455395	1.212234
6	−0.318229	1.752925	0.000000
6	−0.511899	2.455395	−1.212234
1	−0.429925	1.920419	−2.149511
6	−0.834507	3.796517	−1.205051
1	−0.983763	4.316754	−2.142734
6	−0.984507	4.480466	0.000000
1	−1.236558	5.532949	0.000000
6	−0.834507	3.796517	1.205051
1	−0.983763	4.316754	2.142734
5	−1.155199	−2.278806	0.000000
9	−1.132412	−3.649485	0.000000
1	−0.198499	−3.348356	2.524707
1	1.511389	−1.740230	3.914024
9	−2.418591	−1.748375	0.000000
6	2.020840	0.843375	−2.544453
1	1.596714	1.510936	−3.298671
1	2.190269	1.433063	−1.642605
1	2.984572	0.496104	−2.917809
6	2.020840	0.843375	2.544453
1	2.190269	1.433063	1.642605
1	1.596714	1.510936	3.298671
1	2.984572	0.496104	2.917809

Excited rotated minimum S_1 (R2*)

Atom	X	Y	Z
7	1.494474	−1.272382	−0.689808
7	1.581469	1.165857	−0.244786
6	0.379697	1.122589	0.467646
6	0.225250	2.334993	1.156362
6	−0.397564	−0.066170	0.294919
6	0.567848	−1.163962	0.314199
6	0.892752	−2.100511	1.360861
6	1.970766	−2.817946	0.901512
6	1.343911	3.111192	0.834183
6	2.367421	−2.221588	−0.325036
6	2.140996	2.365707	−0.036437
1	1.564965	4.109315	1.195478
1	3.061452	2.636320	−0.529834
1	−1.808440	−2.331457	−0.020277
6	−2.365252	−1.436671	−0.271452
6	−1.743789	−0.175943	−0.151625
6	−2.503037	0.970369	−0.473141
1	−2.026353	1.941997	−0.432510
6	−3.817326	0.852920	−0.872800
1	−4.382185	1.742932	−1.120701
6	−4.416273	−0.401974	−0.987831
1	−5.446070	−0.486722	−1.309039
6	−3.677562	−1.542578	−0.695030
1	−4.133176	−2.520386	−0.788466
5	1.900183	0.131536	−1.374621
9	3.245774	0.097129	−1.636712
1	3.271135	−2.378899	−0.893726
1	2.459745	−3.650074	1.381498
9	1.127615	0.348824	−2.476710
6	−0.901154	2.673496	2.079162
1	−1.540373	3.452301	1.656362
1	−1.523957	1.797208	2.264126
1	−0.527172	3.040691	3.036106
6	0.098379	−2.245437	2.607911
1	−0.110135	−1.259556	3.029996
1	−0.868156	−2.713025	2.403790
1	0.625309	−2.851142	3.342836

Compound 4

Ground state minimum S_0 (GS)

Atom	X	Y	Z
7	−3.348942	1.082065	−0.501116
7	−3.136288	−1.376415	−0.274559
6	−1.932333	−1.185610	0.399015
6	−1.431913	−2.468192	0.774415
6	−1.442317	0.100366	0.614699
6	−2.138179	1.224436	0.171094
6	−1.841195	2.616788	0.248798
6	−2.889516	3.264848	−0.379961
6	−2.355022	−3.386657	0.309483
6	−3.801879	2.292530	−0.831565
6	−3.393403	−2.683544	−0.331813
1	−2.303733	−4.459287	0.415939
1	1.004110	−0.142540	−0.417294
6	1.043665	0.131597	0.631496
6	−0.150499	0.277688	1.326695
6	−0.131634	0.575855	2.683871
1	−1.066544	0.686071	3.220322
6	1.085929	0.727462	3.336396
1	1.104951	0.962205	4.393161
6	2.276039	0.595531	2.640211
1	3.221534	0.744012	3.146368
6	2.270839	0.292747	1.275900
5	−4.043934	−0.259532	−0.855765
9	−5.300700	−0.316009	−0.277539
1	−3.000059	4.330236	−0.511491
9	−4.144703	−0.393672	−2.231338
6	−0.178667	−2.813847	1.515767
1	0.710733	−2.610723	0.916653
1	−0.185352	−3.874978	1.762866
1	−0.079164	−2.249292	2.439670
6	−0.664021	3.299540	0.871929
1	−0.676845	4.356660	0.608148
1	0.282790	2.870974	0.541902
1	−0.686657	3.220809	1.960130
6	−4.617271	−3.226393	−0.982089
1	−5.507361	−2.893915	−0.445078
1	−4.700141	−2.854668	−2.004313
1	−4.586860	−4.313970	−0.990961
6	−5.080937	2.489653	−1.567029
1	−5.053101	1.961070	−2.521205
1	−5.913085	2.076732	−0.994608
1	−5.253157	3.549321	−1.743755
6	3.531298	0.138464	0.549361
6	4.769124	−0.219612	1.011572
6	4.893317	0.122674	−1.241618
1	2.870234	0.734744	−1.975419
6	5.656457	−0.239402	−0.110892
1	5.006928	−0.480704	2.029997
6	5.449368	0.223587	−2.515830
6	7.018127	−0.526935	−0.275089
6	6.794646	−0.056985	−2.644254
1	4.849997	0.506731	−3.372197
6	7.572161	−0.432161	−1.534105
1	7.623993	−0.815117	0.575143
1	7.260406	0.009846	−3.619052
1	8.623182	−0.648340	−1.675820
7	3.600715	0.323827	−0.818487

Excited state minimum S_1 (M*)

Atom	X	Y	Z
7	−2.962239	1.494921	0.131842
7	−3.537238	−0.753735	−0.670250
6	−2.306306	−1.223431	−0.199498
6	−2.180359	−2.601226	−0.592895
6	−1.423703	−0.371751	0.501642
6	−1.781638	0.984540	0.680154
6	−1.134041	2.048855	1.399601
6	−1.940102	3.159340	1.232290
6	−3.349748	−2.908247	−1.262352
6	−3.062793	2.792637	0.453193
6	−4.167297	−1.754091	−1.302506
1	−3.604003	−3.858143	−1.708477
1	1.030147	0.173599	−0.396301
6	1.061069	−0.481867	0.467503
6	−0.145876	−0.883647	1.037249
6	−0.129133	−1.771462	2.111930
1	−1.065770	−2.082010	2.559707
6	1.081207	−2.236742	2.608794
1	1.091690	−2.914332	3.453922
6	2.277706	−1.823059	2.045423
1	3.219419	−2.165376	2.455285
6	2.281681	−0.941337	0.960545
5	−4.054291	0.697896	−0.611787
9	−5.255907	0.758550	0.081700
1	−1.771056	4.143687	1.642848
9	−4.234068	1.202831	−1.893191
6	−1.030593	−3.531364	−0.380872
1	−0.074621	−3.055272	−0.605789
1	−1.145741	−4.400430	−1.028640
1	−0.971883	−3.884927	0.651559
6	0.119728	2.002145	2.211242
1	0.173717	2.888040	2.844020
1	1.018003	1.980311	1.587907
1	0.161156	1.116901	2.847925
6	−5.494813	−1.576457	−1.937435
1	−6.222776	−1.219033	−1.203851
1	−5.440954	−0.812155	−2.718265
1	−5.837919	−2.514975	−2.367558
6	−4.218825	3.622525	0.038629
1	−4.339152	3.591554	−1.047769
1	−5.143562	3.221433	0.469883
1	−4.083355	4.651621	0.364503
6	3.547471	−0.519739	0.359285
6	4.766810	−1.142315	0.358716
6	4.936598	0.811372	−0.809209
1	2.930435	1.359936	−0.408052
6	5.670571	−0.315754	−0.381125
1	4.981454	−2.101400	0.801216
6	5.514823	1.840811	−1.550119
6	7.025016	−0.414098	−0.726670
6	6.852561	1.719334	−1.867954
1	4.937603	2.700333	−1.867790
6	7.601006	0.600076	−1.462306
1	7.608657	−1.272353	−0.417127
1	7.334780	2.499169	−2.443280
1	8.646843	0.538788	−1.734310
7	3.645476	0.653504	−0.364754

Excited TS S_1 (TS*, 52.35i cm^{-1})

Atom	X	Y	Z
7	2.119606	−1.649730	0.473046
7	3.524459	−0.061753	−0.746652
6	2.871450	1.007777	−0.119108
6	3.712223	2.158898	−0.255882
6	1.576475	0.752356	0.463383
6	1.437309	−0.562792	0.999659
6	0.660656	−1.022267	2.112143
6	0.867538	−2.388539	2.177028
6	4.789115	1.757322	−1.026904
6	1.773569	−2.750719	1.152799
6	4.653113	0.384809	−1.312738
1	5.612346	2.378078	−1.347370
1	−0.820073	−0.073939	−0.088564
6	−0.835171	0.979157	0.167753
6	0.392229	1.605472	0.453177
6	0.374672	2.984774	0.695019
1	1.279992	3.500638	0.961444
6	−0.820727	3.689281	0.669072
1	−0.814626	4.750856	0.882808
6	−2.020894	3.051661	0.406764
1	−2.950800	3.605233	0.421686
6	−2.035325	1.676687	0.145745
5	3.041295	−1.540412	−0.764198
9	4.123296	−2.389452	−0.612126
1	0.440814	−3.074926	2.893025
9	2.350681	−1.824067	−1.924378
6	3.584483	3.528294	0.330358
1	2.870808	4.149549	−0.213668
1	4.553895	4.024054	0.282937
1	3.282659	3.491540	1.378570
6	−0.152460	−0.184656	3.042540
1	−0.214615	−0.663243	4.019864
1	−1.171338	−0.032171	2.674784
1	0.286995	0.806935	3.162828
6	5.569291	−0.479747	−2.099941
1	6.120816	−1.155651	−1.442045
1	5.008834	−1.106463	−2.796394
1	6.274746	0.138487	−2.652135
6	2.298402	−4.099205	0.819616
1	2.195577	−4.299598	−0.249174
1	3.365069	−4.161269	1.048035
1	1.762905	−4.858835	1.385786
6	−3.296480	0.994247	−0.148330
6	−4.481206	1.503048	−0.609038
6	−5.398350	0.411907	−0.731536
1	−4.663660	2.533388	−0.867224
6	−4.707929	−0.750206	−0.324082
1	−2.738228	−0.973195	0.425560
6	−6.731716	0.297619	−1.146823
6	−5.309695	−2.007577	−0.306674
6	−7.330790	−0.944443	−1.133654
1	−7.281532	1.172716	−1.470918
6	−6.625866	−2.086895	−0.715015
1	−4.765386	−2.888718	0.009920
1	−8.360678	−1.047177	−1.450403
1	−7.124602	−3.047619	−0.716101
7	−3.430593	−0.371735	0.011359

Excited TS S_1 (R1*, 102.76i cm^{-1})

Atom	X	Y	Z
7	−2.322719	1.693303	−0.018083
7	−3.759784	−0.237207	−0.442733
6	−2.794707	−1.023035	0.177086
6	−3.438259	−2.130480	0.789698
6	−1.426891	−0.568625	0.194272
6	−1.421163	0.821131	0.580210
6	−0.709993	1.526145	1.588125
6	−1.157448	2.837693	1.513156
6	−4.779417	−2.018636	0.450927
6	−2.159847	2.908928	0.521339
6	−4.950061	−0.835876	−0.300468
1	−5.573959	−2.698962	0.718250
1	1.111263	0.258021	0.198791
6	1.014269	−0.791494	−0.044877
6	−0.276498	−1.361233	−0.077238
6	−0.388886	−2.719297	−0.448031
1	−1.370254	−3.161308	−0.553758
6	0.741809	−3.467533	−0.712339
1	0.639726	−4.505201	−1.004091
6	2.007364	−2.898793	−0.636415
1	2.886712	−3.485540	−0.868738
6	2.147664	−1.542872	−0.314484
5	−3.395535	1.167143	−1.029972
9	−4.501897	1.987698	−1.011864
1	−0.822780	3.671121	2.112371
9	−2.853130	1.042244	−2.285626
6	−2.800658	−3.157248	1.668314
1	−1.832216	−2.813875	2.033679
1	−2.638639	−4.101943	1.143066
1	−3.441055	−3.365825	2.526049
6	0.266429	0.947810	2.560910
1	1.302538	1.150695	2.276168
1	0.153371	−0.134608	2.632254
1	0.108971	1.379213	3.549909
6	−6.211820	−0.287787	−0.865111
1	−6.505831	0.622175	−0.338024
1	−6.083039	−0.016216	−1.914005
1	−7.006796	−1.026060	−0.777362
6	−2.944738	4.099901	0.100924
1	−2.947106	4.200940	−0.985518
1	−3.987420	4.001434	0.409822
1	−2.521402	4.997745	0.547455
6	3.477600	−0.931108	−0.268459
6	4.690292	−1.492933	0.022560
6	5.672205	−0.452922	−0.048675
1	4.853556	−2.522004	0.298974
6	4.988023	0.731676	−0.396415
1	2.951625	1.017491	−0.907031
6	7.058577	−0.402953	0.145458
6	5.645650	1.947108	−0.576255
6	7.713674	0.798803	−0.026333
1	7.605981	−1.295876	0.421449
6	7.013404	1.962849	−0.387998
1	5.105743	2.845052	−0.850349
1	8.785279	0.851264	0.116794
1	7.557032	2.889716	−0.518764
7	3.651387	0.421339	−0.496242

Excited rotated minimum S_1 (R2*)

Atom	X	Y	Z
7	−1.933462	1.699221	−0.268212
7	−3.673196	−0.101447	−0.246908
6	−2.806765	−0.972740	0.430447
6	−3.542667	−2.082806	0.869990
6	−1.433483	−0.592610	0.443851
6	−1.430966	0.850058	0.677907
6	−1.125587	1.588262	1.874060
6	−1.351985	2.906250	1.561275
6	−4.850754	−1.877956	0.427227
6	−1.928219	2.946105	0.259614
6	−4.895806	−0.664422	−0.270974
1	−5.697969	−2.527338	0.589399
1	1.132674	0.170001	0.519942
6	0.990211	−0.805376	0.068739
6	−0.310634	−1.339093	−0.016966
6	−0.458882	−2.638901	−0.544848
1	−1.454445	−3.046906	−0.665679
6	0.647933	−3.362779	−0.938938
1	0.517537	−4.356348	−1.349437
6	1.926272	−2.819205	−0.855017
1	2.786072	−3.378942	−1.199511
6	2.100440	−1.525734	−0.358328
5	−3.111979	1.041163	−1.158209
9	−4.072980	2.002472	−1.370294
1	−1.160537	3.772128	2.175740
9	−2.591667	0.539667	−2.316970
6	−3.006290	−3.213457	1.687221
1	−1.986832	−3.003185	2.013058
1	−2.988081	−4.143783	1.114406
1	−3.623982	−3.385195	2.570168
6	−0.570275	0.968773	3.105261
1	0.477289	0.689619	2.965358
1	−1.114556	0.050080	3.335118
1	−0.632876	1.650399	3.951616
6	−6.060154	−0.058191	−0.978792
1	−6.316030	0.913442	−0.555355
1	−5.835428	0.100818	−2.034895
1	−6.920973	−0.719833	−0.898106
6	−2.509909	4.121846	−0.429846
1	−2.370734	4.056383	−1.501377
1	−3.588127	4.152845	−0.244106
1	−2.064196	5.037075	−0.037511
6	3.449756	−0.946349	−0.272774
6	4.650340	−1.551759	−0.055328
6	5.650318	−0.525916	−0.055138
1	4.797408	−2.603961	0.126872
6	4.982289	0.696333	−0.285002
1	2.941609	1.053829	−0.735844
6	7.040501	−0.516059	0.114979
6	5.659536	1.910974	−0.371904
6	7.715337	0.684813	0.034857
1	7.576245	−1.438820	0.301194
6	7.030995	1.887496	−0.210662
1	5.131875	2.838541	−0.556963
1	8.790293	0.705765	0.160147
1	7.589418	2.812739	−0.272785
7	3.638479	0.417970	−0.384381

Compound 5

Ground state minimum S_0 (GS)

Atom	X	Y	Z
7	−1.480471	−1.298738	−0.001269
7	−1.480503	1.298709	0.001611
6	−0.088282	1.214312	−0.000508
6	0.373484	2.563328	−0.021283
6	0.595890	0.000013	−0.000041
6	−0.088251	−1.214299	0.000642
6	0.373553	−2.563298	0.022031
6	−0.761645	−3.353778	0.030216
6	−0.761739	3.353785	−0.028857
6	−1.887724	−2.508624	0.015841
6	−1.887790	2.508599	−0.014746
1	−0.793723	4.432317	−0.046159
1	2.229313	0.234410	2.123857
6	2.780024	0.131825	1.195874
6	2.081736	0.000019	−0.000117
6	2.779892	−0.131793	−1.196182
1	2.229081	−0.234380	−2.124105
6	4.168159	−0.128887	−1.195824
1	4.706939	−0.228422	−2.129702
6	4.863956	0.000012	−0.000266
1	5.946539	0.000010	−0.000325
6	4.168288	0.128914	1.195368
1	4.707170	0.228446	2.129188
5	−2.415021	−0.000019	−0.001005
9	−3.207076	0.001946	−1.137688
1	−0.793600	−4.432302	0.048101
9	−3.210116	−0.002003	1.133501
6	1.777868	3.080019	−0.031274
1	2.290210	2.865083	0.907865
1	1.765509	4.160298	−0.172964
1	2.372509	2.632314	−0.828181
6	1.777954	−3.079929	0.032078
1	1.765641	−4.160194	0.173876
1	2.372567	−2.632119	0.828952
1	2.290300	−2.865045	−0.907072
6	−3.328885	2.881339	−0.020807
1	−3.820601	2.471065	−0.904400
1	−3.831434	2.460658	0.851594
1	−3.437053	3.963998	−0.015556
6	−3.328805	−2.881424	0.022816
1	−3.818667	−2.475864	0.909572
1	−3.839213	−2.456263	−0.846238
1	−3.436943	−3.963987	0.012262

Excited state minimum S_1 (M*)

Atom	X	Y	Z
7	1.484421	−1.226754	−0.039663
7	1.484371	1.226792	0.039666
6	0.086279	1.216741	−0.008094
6	−0.361366	2.581281	−0.088813
6	−0.633398	−0.000025	0.000002
6	0.086328	−1.216765	0.008119
6	−0.361249	−2.581330	0.088884
6	0.784501	−3.354116	0.063159
6	0.784344	3.354125	−0.063061
6	1.908536	−2.498282	−0.010715
6	1.908424	2.498343	0.010737
1	0.833473	4.431641	−0.113790
1	−2.270989	−0.747861	−2.002665
6	−2.819081	−0.424004	−1.125206
6	−2.110819	−0.000038	−0.000010
6	−2.819105	0.423942	1.125165
1	−2.271032	0.747809	2.002632
6	−4.206387	0.420934	1.126056
1	−4.744844	0.744280	2.008182
6	−4.903809	−0.000035	−0.000041
1	−5.986368	−0.000035	−0.000053
6	−4.206363	−0.420998	−1.126126
1	−4.744801	−0.744339	−2.008265
5	2.417664	0.000040	−0.000038
9	3.220793	−0.036382	1.132975
1	0.833686	−4.431627	0.113932
9	3.220694	0.036495	−1.133122
6	−1.756391	3.100977	−0.215459
1	−2.335576	2.539018	−0.950180
1	−1.725031	4.146156	−0.523576
1	−2.304630	3.043458	0.728225
6	−1.756240	−3.101106	0.215572
1	−1.724809	−4.146273	0.529722
1	−2.304500	−3.043653	−0.728103
1	−2.335447	−2.539161	0.950286
6	3.347726	2.852616	0.028345
1	3.835796	2.418572	0.905366
1	3.851565	2.431450	−0.846617
1	3.471889	3.933449	0.036264
6	3.347856	−2.852484	−0.028331
1	3.835924	−2.418329	−0.905298
1	3.851654	−2.431383	0.846686
1	3.472070	−3.933311	−0.036351

Excited TS S_1 (TS*, 60.06i cm^{-1})

Atom	X	Y	Z
7	−1.267996	−1.319544	−0.081865
7	−1.547859	1.097642	0.160648
6	−0.235718	1.296558	−0.287450
6	−0.134004	2.659022	−0.714601
6	0.679862	0.180971	−0.200861
6	0.062122	−1.085560	−0.406710
6	0.598421	−2.286788	−0.972800
6	−0.423299	−3.216994	−0.920786
6	−1.356524	3.242835	−0.428198
6	−1.564635	−2.594505	−0.363780
6	−2.212337	2.259688	0.104114
1	−1.627088	4.274302	−0.597516
1	1.781247	−1.498380	1.484653
6	2.507589	−0.787212	1.109216
6	2.085051	0.199091	0.196260
6	3.049041	1.121184	−0.247293
1	2.778494	1.857902	−0.985432
6	4.354402	1.059894	0.198452
1	5.075591	1.776310	−0.175067
6	4.750205	0.084524	1.103602
1	5.774220	0.043118	1.451567
6	3.814206	−0.839621	1.557374
1	4.102475	−1.598409	2.274223
5	−2.163725	−0.261307	0.600262
9	−3.457498	−0.367309	0.118532
1	−0.382873	−4.242507	−1.255983
9	−2.139203	−0.397217	1.973844
6	0.981153	3.381312	−1.400481
1	1.776887	3.671392	−0.712174
1	0.586721	4.292684	−1.849652
1	1.418333	2.781008	−2.200406
6	1.963431	−2.472142	−1.546185
1	1.949636	−3.260160	−2.299176
1	2.690698	−2.747623	−0.777752
1	2.324548	−1.550669	−2.006066
6	−3.622503	2.415288	0.544619
1	−4.303765	1.951546	−0.172845
1	−3.787131	1.916962	1.501958
1	−3.864501	3.472566	0.637260
6	−2.899792	−3.186765	−0.095580
1	−3.214094	−2.978091	0.929588
1	−3.659780	−2.749134	−0.750046
1	−2.869731	−4.262905	−0.254784

Excited TS S_1 (R1*, 105.19i cm^{-1})

Atom	X	Y	Z
7	−0.219185	−1.374604	1.221775
7	−0.219185	−1.374604	−1.221775
6	0.311112	−0.090886	−1.165151
6	1.130777	0.110932	−2.305917
6	0.059946	0.721742	0.000000
6	0.311112	−0.090886	1.165151
6	1.130777	0.110932	2.305917
6	1.007308	−1.044630	3.065192
6	1.007308	−1.044630	−3.065192
6	0.177739	−1.947563	2.366190
6	0.177739	−1.947563	−2.366190
1	1.468250	−1.244493	−4.020816
1	−0.449878	2.256847	2.148441
6	−0.546123	2.787051	1.210294
6	−0.316381	2.092723	0.000000
6	−0.546123	2.787051	−1.210294
1	−0.449878	2.256847	−2.148441
6	−0.926915	4.113300	−1.203571
1	−1.102058	4.624006	−2.142156
6	−1.104463	4.792395	0.000000
1	−1.401608	5.833017	0.000000
6	−0.926915	4.113300	1.203571
1	−1.102058	4.624006	2.142156
5	−0.991540	−1.976575	0.000000
9	−0.875617	−3.349622	0.000000
1	1.468250	−1.244493	4.020816
9	−2.299507	−1.559126	0.000000
6	1.987814	1.302536	−2.585600
1	2.153999	1.886465	−1.679611
1	1.535944	1.966901	−3.326595
1	2.955147	0.985411	−2.976809
6	1.987814	1.302536	2.585600
1	1.535944	1.966901	3.326595
1	2.153999	1.886465	1.679611
1	2.955147	0.985411	2.976809
6	−0.223801	−3.320353	−2.773696
1	0.247390	−4.066053	−2.130181
1	−1.301972	−3.455713	−2.675074
1	0.070405	−3.499750	−3.806206
6	−0.223801	−3.320353	2.773696
1	−1.301972	−3.455713	2.675074
1	0.247390	−4.066053	2.130181
1	0.070405	−3.499750	3.806206

Excited rotated minimum S_1 (R2*)

Atom	X	Y	Z
7	−1.262740	1.164825	−0.053667
7	−1.188703	−1.306557	−0.459773
6	−0.200977	−1.156898	0.472668
6	−0.413202	−2.084094	1.552165
6	0.756343	−0.059471	0.354565
6	−0.003201	1.130999	0.562120
6	0.226040	2.366567	1.180284
6	−0.899495	3.149333	0.907828
6	−1.478807	−2.859240	1.167859
6	−1.785879	2.392306	0.134024
6	−1.994521	−2.310506	−0.041857
1	−1.887795	−3.718341	1.676427
1	2.309490	1.927570	−0.579520
6	2.782027	0.954697	−0.635854
6	2.056597	−0.180881	−0.214288
6	2.668810	−1.443770	−0.356397
1	2.140412	−2.331590	−0.030095
6	3.937759	−1.561784	−0.894407
1	4.384696	−2.542371	−1.001286
6	4.645555	−0.430741	−1.284250
1	5.642536	−0.524552	−1.694186
6	4.055418	0.826187	−1.149549
1	4.592816	1.709648	−1.471488
5	−1.667995	0.092544	−1.118140
9	−3.036643	0.046668	−1.257597
1	−1.076253	4.163712	1.232821
9	−1.017904	0.295859	−2.301421
6	0.472708	−2.178908	2.741233
1	0.687057	−1.178275	3.129205
1	1.433093	−2.629260	2.477885
1	0.015553	−2.778179	3.526613
6	1.428245	2.721735	1.994269
1	2.031166	3.486638	1.498872
1	2.061195	1.846626	2.147112
1	1.138670	3.114791	2.970256
6	−3.245835	−2.691491	−0.732144
1	−4.059394	−2.040861	−0.399471
1	−3.159966	−2.559481	−1.808536
1	−3.503490	−3.722728	−0.495666
6	−3.089736	2.821243	−0.449991
1	−3.088084	2.707282	−1.535405
1	−3.912052	2.217543	−0.064950
1	−3.271748	3.866786	−0.206677

Compound 6

Ground state minimum S_0 (GS)

Atom	X	Y	Z
7	−1.281255	−1.240346	−0.025055
7	−1.281255	1.240346	0.025051
6	0.105631	1.208052	0.040455
6	0.568445	2.538849	0.143279
6	0.802932	0.000000	0.000002
6	0.105632	−1.208053	−0.040454
6	0.568446	−2.538848	−0.143293
6	−0.546400	−3.352130	−0.176087
6	−0.546400	3.352131	0.176079
6	−1.679568	−2.513986	−0.105382
6	−1.679568	2.513987	0.105375
1	−0.576210	4.427080	0.251068
1	1.603693	2.834562	0.199900
1	2.439750	−1.266371	1.723638
6	2.986748	−0.720461	0.964812
6	2.281356	0.000000	0.000001
6	2.986748	0.720461	−0.964810
1	2.439750	1.266371	−1.723636
6	4.373857	0.714312	−0.966622
1	4.911932	1.265658	−1.727191
6	5.069850	0.000000	0.000001
1	6.152459	0.000000	0.000001
6	4.373857	−0.714312	0.966624
1	4.911932	−1.265658	1.727193
5	−2.219988	0.000000	0.000006
9	−3.009147	0.019506	−1.135726
1	−0.576209	−4.427079	−0.251076
1	1.603693	−2.834561	−0.199919
9	−3.009125	−0.019507	1.135754
6	−3.118608	2.893453	0.126305
1	−3.634155	2.472900	−0.738316
1	−3.601222	2.488895	1.017774
1	−3.222090	3.976521	0.120398
6	−3.118608	−2.893452	−0.126316
1	−3.634174	−2.472843	0.738266
1	−3.601202	−2.488952	−1.017823
1	−3.222092	−3.976521	−0.120344

Excited state minimum S_1 (M*)

Atom	X	Y	Z
7	−1.288573	−1.227259	−0.022142
7	−1.288428	1.227308	0.022178
6	0.111299	1.211029	0.048113
6	0.555897	2.554437	0.163965
6	0.842935	−0.000098	0.000030
6	0.111160	−1.211151	−0.048084
6	0.555572	−2.554611	−0.164097
6	−0.573670	−3.349231	−0.191327
6	−0.573246	3.349196	0.191266
6	−1.706380	−2.497588	−0.104840
6	−1.706069	2.497697	0.104797
1	−0.619105	4.423711	0.275639
1	1.584590	2.864028	0.240964
1	2.485163	−1.493522	1.534888
6	3.025493	−0.850636	0.850598
6	2.308048	−0.000130	−0.000003
6	3.025449	0.850447	−0.850566
1	2.485077	1.493371	−1.534791
6	4.411260	0.846704	−0.851149
1	4.947777	1.501322	−1.526550
6	5.110939	−0.000075	−0.000020
1	6.193353	−0.000054	−0.000028
6	4.411305	−0.846855	0.851144
1	4.947858	−1.501430	1.526558
5	−2.225679	0.000080	0.000053
9	−3.025275	0.020311	−1.133420
1	−0.619670	−4.423736	−0.275756
1	1.584214	−2.864335	−0.241210
9	−3.025191	−0.020065	1.133588
6	−3.144135	2.858698	0.117170
1	−3.645298	2.454293	−0.766545
1	−3.638600	2.414010	0.985694
1	−3.263471	3.939739	0.144022
6	−3.144494	−2.858395	−0.117245
1	−3.645615	−2.453946	0.766472
1	−3.638886	−2.413616	−0.985766
1	−3.263976	−3.939419	−0.144130

Excited TS S_1 (TS*, 64.53i cm^{-1})

Atom	X	Y	Z
7	1.208605	1.238464	−0.072286
7	1.327106	−1.199276	0.054678
6	−0.012574	−1.273536	−0.340130
6	−0.238800	−2.594196	−0.805042
6	−0.853122	−0.106325	−0.220816
6	−0.141814	1.117390	−0.392311
6	−0.566253	2.357266	−0.935368
6	0.517871	3.209181	−0.888146
6	0.935728	−3.298028	−0.621069
6	1.610098	2.485419	−0.345157
6	1.893741	−2.404987	−0.088159
1	1.114002	−4.340551	−0.832754
1	−1.140540	−2.979750	−1.241935
1	−2.277223	1.860992	0.880622
6	−2.895308	1.028713	0.571329
6	−2.284417	−0.103855	−0.010664
6	−3.119221	−1.197380	−0.309854
1	−2.712014	−2.092168	−0.747531
6	−4.482493	−1.143910	−0.073954
1	−5.096869	−1.998893	−0.327107
6	−5.064342	−0.010916	0.478880
1	−6.130146	0.024045	0.663262
6	−4.254828	1.072077	0.809344
1	−4.685759	1.950169	1.273825
5	2.054880	0.089676	0.530442
9	3.331586	0.126279	−0.001971
1	0.558550	4.241371	−1.198763
1	−1.553183	2.556035	−1.319822
9	2.091515	0.162882	1.906010
6	3.304234	−2.689388	0.279367
1	3.988700	−2.238070	−0.443144
1	3.545524	−2.257942	1.252903
1	3.469055	−3.764993	0.305916
6	2.991680	2.965406	−0.086598
1	3.314484	2.689466	0.919482
1	3.695670	2.500547	−0.780948
1	3.036377	4.047008	−0.199227

Excited rotated minimum S_1 (R*)

Atom	X	Y	Z
7	−0.044844	−1.261330	1.219964
7	−0.044844	−1.261330	−1.219964
6	0.403414	0.057521	−1.180536
6	1.094187	0.314816	−2.386794
6	0.201491	0.855770	0.000000
6	0.403414	0.057521	1.180536
6	1.094187	0.314816	2.386794
6	0.997862	−0.830495	3.155127
6	0.997862	−0.830495	−3.155127
6	0.287656	−1.796266	2.401233
6	0.287656	−1.796266	−2.401233
1	1.378082	−0.986766	−4.152401
1	1.607809	1.230623	−2.627571
1	−0.146244	2.463379	2.149624
6	−0.165224	2.990983	1.206624
6	−0.021594	2.269200	0.000000
6	−0.165224	2.990983	−1.206624
1	−0.146244	2.463379	−2.149624
6	−0.377380	4.355471	−1.200475
1	−0.487862	4.880662	−2.140949
6	−0.467442	5.054034	0.000000
1	−0.630940	6.123789	0.000000
6	−0.377380	4.355471	1.200475
1	−0.487862	4.880662	2.140949
5	−0.744240	−1.937293	0.000000
9	−0.480094	−3.291043	0.000000
1	1.378082	−0.986766	4.152401
1	1.607809	1.230623	2.627571
9	−2.092365	−1.673503	0.000000
6	−0.065984	−3.183588	−2.801017
1	0.514323	−3.910051	−2.227929
1	−1.118011	−3.391309	−2.598440
1	0.133401	−3.323092	−3.861968
6	−0.065984	−3.183588	2.801017
1	−1.118011	−3.391309	2.598440
1	0.514323	−3.910051	2.227929
1	0.133401	−3.323092	3.861968

Compound 7

Ground state minimum S_0 (GS)

Atom	X	Y	Z
7	−3.657888	−0.642242	0.629200
7	−2.862002	1.436722	−0.468009
6	−1.688129	0.774781	−0.798373
6	−0.895987	1.656296	−1.568726
6	−1.471164	−0.548654	−0.414862
6	−2.450798	−1.240624	0.298384
6	−2.453160	−2.547096	0.836054
6	−3.666299	−2.719150	1.471557
6	−1.599993	2.837768	−1.679724
6	−4.389735	−1.515871	1.328361
6	−2.819015	2.667136	−0.988429
1	−1.303620	3.735063	−2.198399
1	1.005286	0.293721	0.142985
6	1.015297	−0.649946	−0.390594
6	−0.200333	−1.220273	−0.763752
6	−0.205921	−2.418229	−1.476475
1	−1.149027	−2.852786	−1.782852
6	0.996040	−3.028767	−1.809168
1	0.990275	−3.955406	−2.368791
6	2.200749	−2.466418	−1.422056
1	3.133915	−2.963219	−1.655899
6	2.225151	−1.267086	−0.706181
5	−4.056967	0.830343	0.923212
9	−5.203578	0.866084	−0.447752
1	−4.018178	−3.593527	1.994696
9	−4.246763	1.523606	1.505487
6	−3.938634	3.634735	−0.829635
1	−4.817686	3.284629	−1.373931
1	−4.221657	3.720019	0.220310
1	−3.647470	4.611825	−1.209606
6	−5.745134	−1.180343	1.843449
1	−5.693738	−0.314146	2.505116
1	−6.408907	−0.916914	1.018400
1	−6.161100	−2.026628	2.386096
6	3.501007	−0.674086	−0.303660
6	4.737375	−0.786225	−0.879854
6	4.892810	0.579411	0.942467
1	2.862889	0.290789	1.472503
6	5.643881	0.006204	−0.105952
1	4.962564	−1.335903	−1.779381
6	5.468286	1.403053	1.908612
6	7.013292	0.289950	−0.193357
6	6.820800	1.656564	1.801463
1	4.877847	1.830735	2.709409
6	7.586577	1.107701	0.757476
1	7.610358	−0.130145	−0.993403
1	7.301476	2.292950	2.533314
1	8.643794	1.333432	0.704457
7	3.588903	0.171772	0.785770
1	0.067109	1.420168	−1.992244
1	−1.641082	−3.252324	0.761269

Excited state minimum S_1 (M*)

Atom	X	Y	Z
7	2.783972	1.472029	−0.340015
7	3.743057	−0.619982	0.519176
6	2.539492	−1.273083	0.227845
6	2.656310	−2.614921	0.677089
6	1.454497	−0.595923	−0.376588
6	1.614836	0.779486	−0.671021
6	0.749320	1.687968	−1.337210
6	1.401364	2.904708	−1.379257
6	3.921505	−2.748256	1.213968
6	2.665928	2.740962	−0.754377
6	4.574271	−1.491745	1.105969
1	4.358735	−3.629881	1.655936
1	−1.019287	0.195866	0.245741
6	−1.024951	−0.726384	−0.323851
6	0.202221	−1.289903	−0.688249
6	0.204320	−2.524436	−1.345476
1	1.144939	−2.961232	−1.657173
6	−0.993404	−3.162005	−1.635438
1	−0.982303	−4.107050	−2.163750
6	−2.203354	−2.588414	−1.280410
1	−3.134785	−3.077354	−1.535546
6	−2.230634	−1.361104	−0.611746
5	4.056143	0.877749	0.306715
9	5.144007	1.030397	−0.539628
1	1.048543	3.826097	−1.815653
9	4.316929	1.496823	1.519641
6	5.933574	−1.108512	1.557010
1	6.511537	−0.703444	0.721816
1	5.873559	−0.315016	2.307556
1	6.450353	−1.968827	1.976903
6	3.746010	3.738757	−0.565400
1	4.017455	3.809552	0.491507
1	4.648562	3.424477	−1.097534
1	3.429510	4.714057	−0.928918
6	−3.507839	−0.768331	−0.212865
6	−4.717568	−1.367158	0.015723
6	−4.926472	0.877009	0.376227
1	−2.923848	1.271809	−0.190311
6	−5.639856	−0.340935	0.394640
1	−4.914120	−2.425389	−0.040565
6	−5.526194	2.097505	0.682146
6	−6.995438	−0.327217	0.749084
6	−6.864573	2.079721	1.019747
1	−4.964484	3.023208	0.660056
6	−7.592734	0.877345	1.055708
1	−7.563627	−1.248828	0.778451
1	−7.362985	3.008835	1.264727
1	−8.639717	0.903328	1.328961
7	−3.629896	0.587773	0.029750
1	1.879499	−3.358885	0.620762
1	−0.210408	1.435407	−1.756650

Excited TS S_1 (TS*, 45.87i cm^{-1})

Atom	X	Y	Z
7	−2.430023	−1.517435	−0.293242
7	−3.811513	0.379421	0.394075
6	−2.875324	1.249035	−0.172660
6	−3.525050	2.493457	−0.373221
6	−1.539176	0.774879	−0.440710
6	−1.493224	−0.609254	−0.778085
6	−0.624164	−1.314789	−1.650771
6	−1.025039	−2.634917	−1.636472
6	−4.804747	2.369891	0.132381
6	−2.151982	−2.732306	−0.780033
6	−4.957035	1.043713	0.599833
1	−5.566707	3.131903	0.178738
1	0.907629	−0.114526	−0.001553
6	0.906339	0.941493	−0.238507
6	−0.335426	1.575893	−0.448766
6	−0.325773	2.973685	−0.603141
1	−1.242379	3.518125	−0.749170
6	0.869080	3.676133	−0.595971
1	0.852085	4.749554	−0.736823
6	2.080555	3.027268	−0.429705
1	3.009061	3.582654	−0.455153
6	2.104072	1.641402	−0.231129
5	−3.570989	−1.129668	0.684173
9	−4.709306	−1.850816	0.374064
1	−0.586899	−3.463243	−2.170917
9	−3.194458	−1.332278	1.992840
6	−6.153145	0.427587	1.228154
1	−6.639210	−0.271064	0.538474
1	−5.873364	−0.146907	2.113460
1	−6.864796	1.202788	1.506159
6	−2.935712	−3.944340	−0.429817
1	−3.084437	−4.009370	0.649983
1	−3.929448	−3.902683	−0.881954
1	−2.418355	−4.835468	−0.780114
6	3.374414	0.952623	0.001657
6	4.569371	1.447187	0.451211
6	5.491359	0.354721	0.510265
1	4.756099	2.467377	0.744248
6	4.793122	−0.793358	0.077364
1	2.810390	−0.989097	−0.643959
6	6.835265	0.228989	0.886429
6	5.397130	−2.046936	−0.004191
6	7.436660	−1.009678	0.810035
1	7.391439	1.092866	1.229191
6	6.723881	−2.137475	0.366055
1	4.847037	−2.917137	−0.340492
1	8.474756	−1.120921	1.095539
1	7.224997	−3.095672	0.316566
7	3.506105	−0.406693	−0.209075
1	−3.116935	3.364222	−0.851146
1	0.167181	−0.863667	−2.227614

Excited rotated minimum S_1 (R*)

Atom	X	Y	Z
7	2.520653	1.554676	−0.105837
7	3.883374	−0.451129	0.161994
6	2.811853	−1.200043	−0.322069
6	3.333918	−2.393461	−0.872971
6	1.479296	−0.663383	−0.279990
6	1.495662	0.743819	−0.587703
6	0.673176	1.537035	−1.421172
6	1.181688	2.821927	−1.378444
6	4.699016	−2.369047	−0.659403
6	2.335033	2.801629	−0.556414
6	5.012891	−1.146489	−0.016258
1	5.418388	−3.129797	−0.918776
1	−1.057492	0.229810	−0.254259
6	−0.983382	−0.838154	−0.105127
6	0.291864	−1.441854	−0.094760
6	0.351311	−2.830289	0.154118
1	1.310206	−3.318391	0.251561
6	−0.806222	−3.566051	0.324971
1	−0.736493	−4.628158	0.523415
6	−2.053084	−2.958136	0.276909
1	−2.953547	−3.536199	0.439186
6	−2.144480	−1.576193	0.076966
5	3.696069	0.982993	0.746939
9	4.835588	1.728945	0.533949
1	0.790369	3.699734	−1.868240
9	3.352460	0.928033	2.075885
6	6.349644	−0.664434	0.419797
1	6.674596	0.178674	−0.193752
1	6.318034	−0.308893	1.451160
1	7.076837	−1.469959	0.336401
6	3.227069	3.939109	−0.210725
1	3.417314	3.973336	0.863294
1	4.198020	3.829004	−0.698764
1	2.770883	4.874810	−0.528662
6	−3.454712	−0.921620	0.069713
6	−4.679125	−1.418138	−0.285422
6	−5.632161	−0.361380	−0.127061
1	−4.867504	−2.411748	−0.658606
6	−4.919511	0.765001	0.337521
1	−2.880820	0.946589	0.885246
6	−7.014092	−0.252713	−0.329853
6	−5.544824	1.977202	0.623485
6	−7.637059	0.946390	−0.053004
1	−7.582998	−1.099383	−0.694004
6	−6.908879	2.050655	0.423202
1	−4.983242	2.829382	0.985877
1	−8.704788	1.043429	−0.201676
1	−7.427736	2.976824	0.634634
7	−3.594302	0.407135	0.423367
1	2.758480	−3.149331	−1.380942
1	−0.168718	1.179446	−1.990793

5. Supplementary references

[1] a) W. Wang, M. M. Lorion, O. Martinazzoli, L. Ackermann, *Angew. Chem. Int. Ed.* **2018**, *57*, 10554-10558; b) H. H. Al Mamari, E. Diers, L. Ackermann, *Chem. Eur. J.* **2014**, *20*, 9739-9743; c) L. Mendive-Tapia, C. Zhao, A. R. Akram, S. Preciado, F. Albericio, M. Lee, A. Serrels, N. Kielland, N. D. Read, R. Lavilla, M. Vendrell, *Nat. Commun.* **2016**, *7*, 10940; d) N. Kaewchangwat, R. Sukato, V. Vchirawongkwin, T. Vilaivan, M. Sukwattanasinitt, S. Wacharasindhu, *Green Chem.* **2015**, *17*, 460-465.

[2] W. C. Chan, P. D. White, *Fmoc solid phase peptide synthesis: a practical approach*, Oxford University Press, Oxford, UK, **2000**.

[3] Y. Zhao, D. G. Truhlar, *Theor. Chem. Acc.* **2008**, *120*, 215-241.

[4] M. J. Frisch, G. W. Trucks, H. B. Schlegel, G. E. Scuseria, M. A. Robb, J. R. Cheeseman, G. Scalmani, V. Barone, B. Mennucci, G. A. Petersson, H. Nakatsuji, M. Caricato, X. Li, H. P. Hratchian, A. F. Izmaylov, J. Bloino, G. Zheng, J. L. Sonnenberg, M. Hada, M. Ehara, K. Toyota, R. Fukuda, J. Hasegawa, M. Ishida, T. Nakajima, Y. Honda, O. Kitao, H. Nakai, T. Vreven, J. A. Montgomery, Jr., J. E. Peralta, F. Ogliaro, M. Bearpark, J. J. Heyd, E. Brothers, K. N. Kudin, V. N. Staroverov, R. Kobayashi, J. Normand, K. Raghavachari, A. Rendell, J. C. Burant, S. S. Iyengar, J. Tomasi, M. Cossi, N. Rega, J. M. Millam, M. Klene, J. E. Knox, J. B. Cross, V. Bakken, C. Adamo, J. Jaramillo, R. Gomperts, R. E. Stratmann, O. Yazyev, A. J. Austin, R. Cammi, C. Pomelli, J. W. Ochterski, R. L. Martin, K. Morokuma, V. G. Zakrzewski, G. A. Voth, P. Salvador, J. J. Dannenberg, S. Dapprich, A. D. Daniels, Ö. Farkas, J. B. Foresman, J. V. Ortiz, J. Cioslowski, D. J. Fox, Gaussian 09, revision D.01; Gaussian, Inc.: Wallingford, CT, **2009**.

[5] a) S. Chibani, A. D. Laurent, B. Le Guennic, D. Jacquemin, *J. Chem. Theory Comput.* **2014**, *10*, 4574-4582; b) S. Chibani, B. Le Guennic, A. Charaf-Eddin, A. D. Laurent, D. Jacquemin, *Chem. Sci.* **2013**, *4*; c) S. Chibani, B. Le Guennic, A. Charaf-Eddin, O. Maury, C. Andraud, D. Jacquemin, *J. Chem. Theory. Comput.* **2012**, *8*, 3303-3313.

[6] K. H. Drexhage, *J. Res. Natl. Bur. Stand. A, Phys. Chem.* **1976**, *80*, 421-428.

[7] L. Mendive-Tapia, R. Subiros-Funosas, C. Zhao, F. Albericio, N. D. Read, R. Lavilla, M. Vendrell, *Nat. Protoc.* **2017**, *12*, 1588-1619.
